# MAZ-mediated up-regulation of BCKDK reprograms glucose metabolism and promotes growth by regulating glucose-6-phosphate dehydrogenase stability in triple-negative breast cancer

**DOI:** 10.1038/s41419-024-06835-y

**Published:** 2024-07-18

**Authors:** Yan Li, Yuxiang Lin, Yali Tang, Meichen Jiang, Xiaobin Chen, Hanxi Chen, Qian Nie, Jinqiao Wu, Xin Tong, Jing Li, Liuwen Yu, Jialin Hou, Wenhui Guo, Lili Chen, Minyan Chen, Jie Zhang, Shuhai Lin, Fangmeng Fu, Chuan Wang

**Affiliations:** 1 Department of Breast Surgery, Fujian Medical University Union Hospital, Fuzhou, Fujian Province 350001 China; 2 Department of General Surgery, Fujian Medical University Union Hospital, Fuzhou, Fujian Province 350001 China; 3 Breast Cancer Institute, Fujian Medical University, Fuzhou, Fujian Province China; 4 School of Life Sciences, Xiamen University, Xiamen, Fujian Province China; 5 Department of Pathology, Fujian Medical University Union Hospital, Fuzhou, Fujian Province 350001 China

**Keywords:** Cancer metabolism, Prognostic markers

## Abstract

Tumour metabolic reprogramming is pivotal for tumour survival and proliferation. Investigating potential molecular mechanisms within the heterogeneous and clinically aggressive triple-negative breast cancer (TNBC) subtype is essential to identifying novel therapeutic targets. Accordingly, we investigated the role of branched-chain α-keto acid dehydrogenase kinase (BCKDK) in promoting tumorigenesis in TNBC. We analysed The Cancer Genome Atlas dataset and immunohistochemically stained surgical specimens to investigate BCKDK expression and its prognostic implications in TNBC. The effects of BCKDK on tumorigenesis were assessed using cell viability, colony formation, apoptosis, and cell cycle assays, and subsequently validated in vivo. Metabolomic screening was performed via isotope tracer studies. The downstream target was confirmed using mass spectrometry and a co-immunoprecipitation experiment coupled with immunofluorescence analysis. Upstream transcription factors were also examined using chromatin immunoprecipitation and luciferase assays. BCKDK was upregulated in TNBC tumour tissues and associated with poor prognosis. BCKDK depletion led to reduced cell proliferation both in vitro and vivo. MYC-associated zinc finger protein (MAZ) was confirmed as the major transcription factor directly regulating BCKDK expression in TNBC. Mechanistically, BCKDK interacted with glucose-6-phosphate dehydrogenase (G6PD), leading to increased flux in the pentose phosphate pathway for macromolecule synthesis and detoxification of reactive oxygen species. Forced expression of G6PD rescued the growth defect in BCKDK-deficient cells. Notably, the small-molecule inhibitor of BCKDK, 3,6-dichlorobenzo(b)thiophene-2-carboxylic acid, exhibited anti-tumour effects in a patient-derived tumour xenograft model. Our findings hold significant promise for developing targeted therapies aimed at disrupting the MAZ/BCKDK/G6PD signalling pathway, offering potential advancements in treating TNBC through metabolic reprogramming.

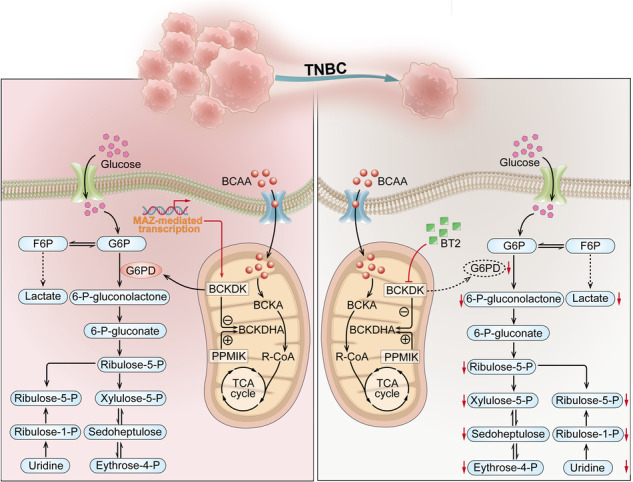

## Introduction

Triple-negative breast cancer (TNBC) represents a highly heterogeneous and clinically aggressive subtype with an unfavourable prognosis, compounded by the absence of targeted therapies [1]. Cellular metabolic reprogramming is a crucial driver of tumorigenesis and metastasis, facilitating the supply of energy, nutrients, and essential building blocks to tumour cells [2]. The Warburg effect, a characteristic metabolic shift in cancer cells, generates intermediate metabolites crucial for nucleic acid and fatty acid synthesis, thereby supporting an elevated proliferation rate [3].

In recent years, several enzymes implicated in glucose and lipid metabolism, such as hexokinase-2 (HK-2) [4], malic enzyme 2 (ME2) [5], and glucose-6-phosphate dehydrogenase (G6PD) [6], have garnered research attention. The role of branched-chain amino acids (BCAAs), closely associated with glucose utilisation and lipid metabolism, remains poorly understood [7]. Branched-chain α-keto acid dehydrogenase kinase (BCKDK), a key enzyme located in mitochondria involved in branched-chain amino acid metabolism [8], was highly expressed in TNBC based on proteomics studies (log2FC = 2.76, *P* = 5.69E-15), correlating with poor prognosis (HR = 1.46, 95% CI = 1.02–2.07, *P* = 0.036) in our unpublished data. Although previous reports have associated elevated BCKDK expression with high-grade ovarian cancer [9] and chemotherapy-resistant disease [10], the regulatory mechanisms of BCKDK expression and its role in metabolic reprogramming within TNBC remain unclear.

Notably, the metabolic reprogramming of tumour cells plays a crucial role in enabling robust biosynthesis and antioxidant defense [11]. The pentose phosphate pathway (PPP), a pivotal component of glucose metabolism utilising glucose-6-phosphate (G6P) as its primary substrate, represents a crucial step [12]. In breast cancer cells, to meet the demands of rapid cancer cell growth, the PPP is frequently upregulated, providing biosynthetic building blocks and reducing equivalents, including nicotinamide adenine dinucleotide phosphate (NADPH) nucleotide precursors. The modulation occurs through G6PD and evidence indicates a significant association between elevated G6PD expression and poor prognosis. Furthermore, G6PD has become targets for cancer therapy [13], with recent studies revealing their involvement in cisplatin resistance [14].

Despite these insights, the precise molecular mechanisms governing metabolic reprogramming and its impact on the proliferation of TNBC cells remain elusive. A comprehensive understanding of these mechanisms is crucial for developing targeted therapies against this disease. Accordingly, we aimed to elucidate the expression pattern and role of BCKDK in TNBC, as well as its correlation with prognoses in patients with TNBC. Notably, we aimed to obtain insights into BCKDK in TNBC development and putative metabolic reprogramming. Collectively, we believe that our findings would help highlight the role of critical pathways in the development of TNBC and help identify potential therapeutic targets against TNBC.

## Methods

### Human tissues and public datasets

The mRNA microarray dataset for patients with TNBC was extracted from The Cancer Genome Atlas (TCGA) database (https://portal.gdc.cancer.gov/). Kaplan–Meier Plotter (http://www.kmplot.com) [15] and the Gene Expression Omnibus (GEO) database (http://www.ncbi.nlm.nih.gov/geo/), including GSE21653 [16] and GSE31448 [17], were also employed. Additionally, 71 paired paraffin-embedded TNBC specimens and corresponding adjacent tissues were procured from Fujian Medical University Union Hospital. Clinical variables including age, tumor size, lymph nodes metastasis, grade, and follow-up information for each patient was collected retrospectively. Disease-free survival (DFS) was defined as the time from the date of diagnosis to the date of relapse/metastasis, and overall survival (OS) was defined as the time from the date of diagnosis until death from any cause.

The potential upstream transcription factors were predicted using JASPAR (http://jaspar.genereg.net/) [18], GeneCards (https://www.genecards.org/) [19], and PROMO databases (http://alggen.lsi.upc.es/cgibin/promo_v3/promo/promoinit.cgi?dirDB=TF_8.3) [20, 21]. Tumor Immune Estimation Resource (TIMER) (https://cistrome.shinyapps.io/timer/) [22] acts as a web server for exploring correlation between BCKDK and transcription factors in Breast Invasive Carcinoma and BRCA-Basal. The expression levels is log2 RSEM.

### Immunohistochemistry (IHC) staining

The expression of BCKDK in TNBC tissues and corresponding non-tumour tissues was measured by IHC staining analysis according to the standard staining procedure. IHC staining scores were calculated as the product of the proportion and intensity of stained tumour cells. The proportion of stained positive cells ranged from 1 to 4: 1, 0–25%; 2, 26–50%; 3, 51–75%; and 4, 75–100%, while staining intensity scores ranged from 0 to 3: 0, no staining; 1, weak staining; 2, moderate staining; and 3, strong staining. A score of 8–12 and 0–7 indicated high and low expression, respectively. Two independent pathologists, blinded to each patient, evaluated the IHC staining scores.

### Cell culture and stable transfection using lentiviral infection

Human breast cancer cell lines (HS578T, MDA-MB-231, HCC1806, and BT-549), an immortalised normal breast epithelial cell line (MCF10A), and HEK293T cell lines were purchased from the Cell Bank of Type Culture Collection of The Chinese Academy of Sciences. MDA-MB-231, HS578T, BT-549, and HEK293T were cultured in Dulbecco’s modified Eagle medium (DMEM, HyClone), and HCC1806 cells were cultured in RPMI 1640 (Gibco) medium with 10% fetal bovine serum (Gibco) in an incubator with standard conditions (5% CO_2_, 37 °C). BCAA-deficient culture media were customised from Procell. Leucine (L8912), isoleucine (I7403), and valine (V0030000) from Sigma-Aldrich were individually added. To inhibit the mTOR signalling pathway, 50 nM everolimus (SC0177, Beyotime Biotechnology) was used. Additionally, 50 μg/mL cycloheximide (Selleck, S7418) and 20 μM MG132 (Selleck, S2619, USA) were used to inhibit protein synthesis and the proteasome, respectively.

Short hairpin RNA (shRNA) targeting BCKDK was subcloned into the GV493 lentiviral shRNA vector (Genechem, China). For overexpressing BCKDK, MYC-associated zinc finger protein (MAZ), and G6PD, constructs were generated by subcloning PCR-amplified full-length human cDNA. These lentiviral vectors were packaged into viruses to infect cells as previously described [23]. The sense sequences of shRNAs are listed in Table S1.

### RNA extraction and quantitative reverse transcription-polymerase chain reaction (qRT-PCR)

Total RNAs were extracted from cells using Trizol solution (Invitrogen, USA) and quantified using a micro-spectrophotometer (Hangzhou Allsheng Instruments Co., Ltd. China). The high-capacity cDNA reverse transcription kit was used to synthesise cDNA with 1 µg RNA as a template using All-In-One 5X RT MasterMix (ABM, G592). The PCR assay was performed using SYBR Green qPCR Mix (ABM, G891). Primer sequences are presented in Table S2. Results were computed via the 2^−ΔΔCt^ method with β-actin as the endogenous control [24].

Differentially expressed genes using RNA-seq analysis between the knockdown and control groups in BT-549 were obtained for Kyoto Encyclopedia of Genes and Genomes (KEGG) pathway enrichment analysis as previously described [25].

### Immunoprecipitation (IP), mass spectrometry (MS) sample preparation, and data analysis

Total proteins were isolated using radio-immunoprecipitation assay (RIPA) lysis buffer containing protease and phosphatase inhibitors. Western blotting was performed, as described previously [23]. Antibodies are listed in Table S3.

BT-549 cells transfected with 3×Flag-BCKDK were lysed in a buffer containing 50 mM Tris-HCl (pH 7.4), 150 mM NaCl, 1 mM ethylenediaminetetraacetic acid (EDTA), and 1% Triton X-100 for 40 min. Following centrifugation at 14,000 × *g* for 20 min, the supernatants were collected and incubated with anti-Flag M2 affinity gel (Sigma-Aldrich, A2220) at 4 °C overnight with rotation. Samples were washed four times with lysis buffer and then incubated with 3×Flag peptides (ApexBio, A6001) for 15 min with vigorous agitation. Proteins were resuspended in 5× SDS sample loading buffer, boiled, and subjected to sodium dodecyl sulphate-polyacrylamide gel electrophoresis (SDS-PAGE) followed by western blotting for co-immunoprecipitation (co-IP) analysis.

After IP, elutes were initially reduced in 20 mM dithiothreitol (Sigma) at 95 °C for 5 min and subsequently alkylated in 50 mM iodoacetamide (Sigma) for 30 min in the dark at 25 °C. Following alkylation, samples were transferred to a 10-kDa centrifugal spin filter (Millipore) and sequentially washed thrice with 200 μL of 8 M urea and twice with 200 μL of 50 mM ammonium bicarbonate, followed by centrifugation at 14,000 × *g*. Subsequently, trypsin digestion was performed by adding trypsin (Promega) at 1:50 (enzyme/substrate, m/m) in 200 μL of 50 mM ammonium bicarbonate at 37 °C for 16 h. Peptides were recovered by transferring the filter to a new collection tube and spinning at 14,000 × *g*. Peptides were desalted using StageTips. MS experiments were performed on a nanoscale EASY-nLC 1200 UHPLC system (Thermo Fisher Scientific) connected to an Orbitrap Fusion Lumos equipped with a nanoelectrospray source (Thermo Fisher Scientific). Mobile phase A contained 0.1% formic acid (v/v) in water; mobile phase B contained 0.1% formic acid in 80% acetonitrile. The peptides were dissolved in 0.1% formic acid with 2% acetonitrile and separated on an RP-HPLC analytical column (75 μm × 25 cm) packed with 2-μm C18 beads (Thermo Fisher Scientific) using a linear gradient ranging from 9–26% acetonitrile in 90 min and followed by a linear increase to 42% B in 20 min at a flow rate of 300 nL/min. The Orbitrap Fusion Lumos acquired data in a data-dependent manner, alternating between full-scan MS and MS2 scans. The spray voltage was set at 2.2 kV, and the temperature of the ion transfer capillary was 300 °C. The MS spectra (350−1500 m/z) were collected with 120,000 resolutions, AGC of 4 × 10^5^, and 50 ms maximal injection time. Selected ions were sequentially fragmented in a 3 s cycle using HCD with 30% normalised collision energy, specified isolated windows 1.6 m/z, 30,000 resolutions. AGC of 5 × 10^4^ and 80 ms maximal injection time were used, and dynamic exclusion was set to 30 s. Unassigned ions or those with a charge of 1+ and >7+ were rejected for MS/MS. Raw data were processed using Proteome Discoverer (PD, version 2.1), and MS/MS spectra were searched against the reviewed Swiss-Prot human proteome database. All searches were performed with a precursor mass tolerance of 20 ppm, fragment mass tolerance of 0.02 Da, oxidation (Met) (+15.9949 Da), and acetylation (protein N-terminus) (+42.0106 Da) as variable modifications, carbamidomethylation (Cys) (+57.0215 Da) as a fixed modification, and three trypsin missed cleavages allowed. Only peptides with at least six amino acids in length were considered. The peptide and protein identifications were filtered based on PD to control the false discovery rate <1%. At least one unique peptide was required for protein identification.

### Cell Counting Kit-8 (CCK-8) and colony formation assay

Following transfection and/or treatment, 2 × 10^4^ cells were seeded into a 96-well plate for 24, 48, 72, and 96 h. Subsequently, 10 μL CCK-8 solution (Dojindo Laboratories, Japan) was replenished into the culture medium for 2 h. After shaking, the absorbance of each well was measured at 450 nm using multimode reader (SpectraMax i3X, Molecular Device, American).

For the colony formation assay, cells were cultivated in a 6-well plate with 500 cells/well for 2 weeks. Subsequently, cells were rinsed using phosphate-buffered saline (PBS), fixed with 4% paraformaldehyde solution (Beyotime Biotechnology), and dyed using Giemsa solution (Solarbio, China). The colony number of each group was counted under a microscope.

### Apoptosis assay

An Annexin V-APC Apoptosis Detection Kit (KeyGEN BioTECH, KGA1026) was used to evaluate cell apoptosis. For apoptosis analysis, 2 × 10^5^ cells/dish were seeded into 6-well plates in complete media overnight and then treated with 100 µM of 3,6-dichlorobenzo(b)thiophene-2-carboxylic acid (BT2) or dimethyl sulfoxide (DMSO). After 24 h, the cells were harvested and washed followed by 4 min of centrifugation at 1000 rpm. The cells were resuspended with 500 μL of binding buffer and transferred to a 5 mL format flow tube. Each tube was mixed with 5 µL of Annexin V-APC and 5 µL of 7-AAD. The tube wall was flicked by hand to mix the cells, which were then incubated in the dark for 15 min at 25 °C. Finally, the apoptosis rate was determined using a flow cytometer (BD LSRFortessa™ X-20).

### Cell cycle assay

The Cell Cycle Analysis Kit (Beyotime, C1052) was used for the cell cycle assay. Cells were seeded (2 × 10^5^/well) into 6-well plates and treated with BT2 (ZZBIO, ZC-26488, 100 µM) or DMSO for 24 h. Cells were collected, washed, and fixed in 70% ice ethanol overnight at 4 °C. The fixed cells were then centrifuged and washed with PBS twice. After washing, resuspended cells were incubated with 500 µL of staining buffer, 10 µL of RNase (50×), and 25 µL of propidium iodide (20×) for 30 min at 25 °C in the dark. The DNA contents were detected using a flow cytometer (BD LSRFortessa™ X-20).

### Bioenergetic analysis

The oxygen consumption rate (OCR) and extracellular acidification rate (ECAR) were measured using a Seahorse XFe24 bioanalyzer (Agilent Technologies, Santa Clara, California, USA) with the Seahorse XF Mito Stress test kit and Glycolytic Rate Assay Kit (Agilent Technologies). To analyse mitochondrial respiration, Oligomycin A (1 μM), fluorocarbonyl cyanide phenylhydrazone (1.5 μM), and rotenone/antimycin A (0.5 μM) were added to the injection ports. For ECAR, Antimycin/rotenone (0.5 μM) and 2-deoxy glucose (2-DG, 50 mM) were added. Initial data analysis was performed using Wave software (Agilent Technologies).

### Electron microscopic imaging

Cells were seeded into 10-cm plates and collected into a 1.5 mL tube. Subsequently, the cells were fixed with 2.5% glutaraldehyde overnight at 4 °C, rinsed with PBS, and postfixed with 1% osmium tetroxide. Next, the samples were rinsed, dehydrated in ethanol and acetone, and embedded in Spurr resin. Finally, 70-nm ultrathin sections were stained with 2% uranyl acetate and lead citrate, followed by visualisation using a Tecnai Spirit transmission electron microscope.

### Mitochondrial membrane potential (Δψm) assay

For the Δψm assay, cells were seeded (3 × 10^5^/well) into 6-well plates for 24 h. Following a single PBS wash, cells were incubated with 1 mL of staining buffer and 1 µL of TMRE (1000×) at 37 °C for 30 min in the dark. Subsequently, the cells were collected, washed once with PBS, and Δψm was determined using a flow cytometer (BD LSRFortessa™ X-20).

### Reactive oxygen species (ROS) measurement, NADPH, and ATP production

The Reactive Oxygen Species Assay Kit (Solarbio, CA1420) was used for the cell ROS assay. Cells (3 × 10^5^/well) were seeded into 6-well plates for 24 h and washed once with PBS. After washing, cells were incubated with 1 mL of staining buffer and 1 µL of tetramethylrhodamine ethyl ester (5 mM) at 37 °C for 30 min in the dark. Subsequently, the cells were collected and washed once with PBS. The cytosolic ROS was determined using a flow cytometer (BD LSRFortessa™ X-20).

NADPH levels were measured using the NADP^+ ^/NADPH assay (Beyotime Biotechnology, S0179). NADP^+^ /NADPH extraction solution was added to 2 × 10^6^ cells and the samples were heated at 60 °C for 30 min to destroy NADP^+^. The chromogenic agent was added to the samples for 30–45 min, followed by measuring absorbance at 450 nm. ATP was measured using the Enhanced ATP Assay Kit (Beyotime Biotechnology, S0027).

### [U-^13^C6] incubation

BT-549 cells were cultured in 15-cm plates. Upon reaching full confluence, they were washed thrice with PBS. For [U-^13^C6] glucose metabolic flux, the culture medium was replaced with 4.5 g/L of [C1,C2-^13^C6] glucose medium supplemented with 10% dialysed FBS for 24 h. For [U-^13^C6] leucine metabolic flux, [U-^13^C6] leucine tracer was added to leucin deficient DMEM medium.

### Extraction of metabolites

Cells (5 × 10^6^) were extracted with 0.8 mL 80% methanol solution at −80 °C after washing three times with ice-cold PBS. Samples were collected into 2 mL tubes, vortexed for 5 min, and centrifuged at 16,600 × *g* and 4 °C for 15 min. The supernatants were evaporated to dryness in a vacuum centrifuge (Labconco Corporation) and subsequently stored at −80 °C until the following analysis.

### Derivatisation of metabolites

Metabolites were dissolved in 15 µL of 50:50 (w/w) acetonitrile:water and then mixed with 5 μL of 175 mM 3-NPH in a 75% methanol aqueous solution, 5 μL of 100 mM EDAC in methanol, and 25% pyridine. After the reaction at 30 °C for 1 h, 25 µL of 0.2 mg/mL BHT was added to the mixtures. The mixtures were vortexed and centrifuged at 16,600 × *g* for 15 min at 4 °C. The supernatant (20 µL) was then prepared for LC–MS.

### LC–MS detection

For labelled and unlabelled LC–MS analysis of intracellular metabolites, each sample (5 µL) was injected and analysed with a QTRAP (Sciex, QTRAP 5500) interfaced with a UPLC system (AB Sciex, ExionLC AD system). The sample was loaded onto a ZIC-pHILIC column (SeQuant, 5 µm, 100 × 2.1 mm, Merck). Buffer A was 15 mM ammonium carbonate, pH 9.6, and buffer B was 90% acetonitrile. The gradient programme was 0 min, 5% A, 95% B; 1 min, 5% A, 95% B; 15 min, 55% A, 80% B; 17 min, 55% A, 80% B; 17.5 min, 5% A, 75% B; 22 min, 5% A, 95% B; and the flow rate was 0.2 mL/min, with a column temperature of 40 °C. Data were acquired using MultiQuant.

For the derivatisation of metabolites, chromatographic separation was performed on a QTRAP (Sciex, QTRAP 6500 plus), with an ACQUITY UPLC BEH C18 column (2.1 mm × 100 mm, 1.7 µm, Waters) and the following gradient: 0 min, 95% A, 5% B; 1 min, 95% A, 5% B; 5 min, 70% A, 30% B; 9 min, 50% A, 50% B; 11 min, 22% A, 78% B; 13.5 min, 5% A, 95% B; 14 min, 100% B; 16 min, 100% B; 16.1 min, 95% A, 5% B; and 18 min, 95% A, 5% B. Mobile phase A was 0.1% formic acid in water. Mobile phase B was 0.1% formic acid in acetonitrile. The flow rate was 0.4 mL/min, and the column temperature was kept at 40 °C. The autosampler was kept at 4 °C, and the injection volume was 5 µL. Mass data acquisition for derivatised endogenous metabolites was performed using a QTRAP 6500+ mass spectrometer equipped with an electrospray ion source in multiple reaction monitoring mode. Metabolite peak review and peak area integration were performed using MultiQuant (Sciex, Framingham, MA), and labelled data were manually corrected for natural isotope abundance.

### Chromatin immunoprecipitation (ChIP) assay

The ChIP assay was used to investigate the interaction between MAZ and BCKDK according to the protocol of SimpleChIP^®^ Plus Enzymatic Chromatin IP Kit (Magnetic Beads) (#9003, Cell Signalling Technology). Micrococcal Nuclease (0.2 µL/4 × 10^6^ cells) and 1 µg antibody are used. The primer pairs are presented in Table S2.

### Dual-luciferase activity assay

The dual-luciferase activity assay was performed to further elucidate the connection between MAZ and BCKDK using the Dual-Luciferase^®^ Reporter Assay System (Promega, E1910). HEK 293 T cells were cotransfected into MAZ and BCKDK promoter full-length or P1 or P2 or P3 or mutant. After 48 h, the total proteins in the cells were extracted. The cell lysis supernatant (15 μL) was incubated with 40 μL Firefly luciferase reaction buffer and 40 μL Renilla detection kit. The relative luciferase activity was monitored using a microplate reader.

### Protein structure and interaction analysis

The Online SWISS-MODEL (https://swissmodel.expasy.org/interactive, accessed on 18 August 2022) software was used to predict the secondary structures of BCKDK and G6PD. The models were visualised in PyMol, and images were generated using PyMol.

### Immunofluorescence co-localisation analysis

Cells (8 × 10^4^/well) seeded onto a coverslip (Thermo Fisher Scientific, USA) were fixed with 4% paraformaldehyde for 30 min, permeabilised in 0.5% Triton X-100 for 30 min, and blocked in Blocking Solution for 60 min. Subsequently, primary antibodies (G6PD and BCKDK, both diluted 1:50) were added to the cells and incubated overnight at 4 °C. Alexa Fluor 488 (green for BCKDK) or Alexa Fluor 555 (red for G6PD) secondary antibodies were used in the dark for 2 h. The nuclei were stained using 4’,6-diamidino-2-phenylindole, and images were captured using LSM780 confocal microscopy (Zeiss, Oberkochen, Germany). Antibodies are listed in Table S3.

### Xenograft tumour models

BALB/c nude mice (4-6 weeks old, female) were weighed and randomly divided into the shBCKDK and shCtrl groups (12 animals). BT-549 cells (5 × 10^6^ cells in 100 µL PBS) harbouring empty vector and stable knockdown of BCKDK were subcutaneously injected on the right flank. Body weight and tumour volumes were measured twice a week. After 22 days, all mice were executed by cervical dislocation.

Patient-derived tumour xenograft (PDX) models were generated from fresh tumour samples of patients with TNBC, which were subcutaneously implanted on the right flank (F0). After reaching the appropriate volume, the tumours were excised and divided into equal pieces for implanting the next generation. When the tumours became palpable, the tumour-bearing mice were randomised to two groups (12 animals). BT2 (MedChemExpress, HY-114855, 20 mg/kg/day) or PBS was intraperitoneally injected daily for 1 week. Tumour size was evaluated using calliper measurements every 2–3 days by the following formula:

V = 0.5 × D × W^2^ (V, volume; D, the larger diameter; and W, width).

The mice were sacrificed after 17 days, and the tumour tissues were excised from mice, weighed, and fixed in a 10% formalin solution for further analysis.

### Statistical analyses

Statistical analysis was conducted using GraphPad Prism 8.0.1 software (GraphPad Software Inc., La Jolla, CA, USA). All experiments were repeated at least thrice (*n* ≥ 3). Assumptions, including normality of data and homogeneity of variances were assessed. Common tests used to assess normality include the Shapiro-Wilk test and the Kolmogorov-Smirnov test. For testing homogeneity of variances, Levene’s test and the Bartlett test are employed. When dealing with non-normally distributed data, non-parametric tests such as the Kruskal-Wallis Test (for comparing multiple groups) and the Mann–Whitney *U* Test (for comparing two groups) are used as alternatives to one-way ANOVA and t-tests, respectively. Statistical significance was set at *P* < 0.05.

## Results

### BCKDK up-regulation in TNBC and its association with poor prognosis

**Fig. 1:**
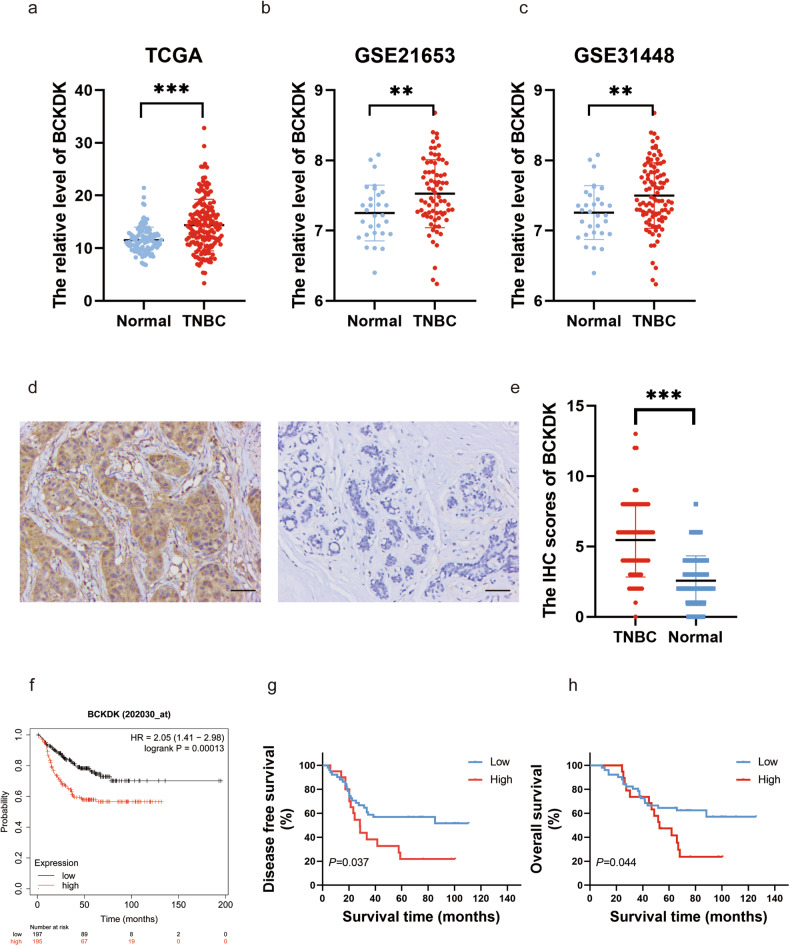
BCKDK expression in TNBC and its prognostic significance. **a**–**c** Evaluation of *BCKDK* mRNA expression in TNBC and normal breast tissues using TCGA and GEO databases (GSE21653 and GSE31448). **d**, **e** Representative immunohistochemistry (IHC) images (×200, Scale bar: 50 μm) and scores of BCKDK in tumour and adjacent normal tissues for 71 patients with TNBC. **f**–**h** Kaplan–Meier analysis in Kaplan–Meier plotter for relapse-free survival and our data for disease-free survival (DFS) and overall survival (OS) with varying BCKDK expression in TNBC. **P < 0.01, ***P < 0.001.

Analyses of the TCGA database revealed a significant up-regulation of BCKDK in TNBC. Consistent findings were observed in the GEO databases GSE21653 and GSE31448, further confirming elevated BCKDK expression in TNBC tissues compared to normal. Additionally, IHC staining assay conducted on 71 pairs of TNBC tissues from our hospital validated the marked increase in BCKDK levels within TNBC tissues (Fig. 1a–e).

Kaplan–Meier Plotter using public databases demonstrated a statistically significant association between increased BCKDK expression and worse relapse-free survival in patients with TNBC (Fig. 1f). Univariate and multivariate analyses in our data indicated that BCKDK serves as an independent prognostic factor for both DFS and OS (HR = 2.193, 95% CI = 1.133–4.245, *P* = 0.020, and HR = 2.088, 95% CI = 1.048–4.159, *P* = 0.036, respectively; Table 1 and Table 2; Fig. 1g, h).

**Table 1:** Univariate and multivariate cox proportional hazard model for disease-free survival (DFS) for TNBC patients.

Characteristics	Univariate analysis		Multivariate analysis	
	Hazard ratio (95% CI)	*P* value	Hazard ratio (95% CI)	*P*^*a*^ value
*Age (years)*				
≤50	Reference			
>50	0.798 (0.422−1.511)	0.489		
Tumor size				
≤2 cm	Reference			
>2 cm	1.184 (0.575−2.438)	0.647		
Lymph nodes metastasis				
No	Reference		Reference	
Yes	3.320 (1.667−6.609)	**<0.001**	3.511 (1.758−7.015)	**<0.001**
Grade				
I + II	Reference			
III	0.655 (0.338−1.269)	0.210		
BCKDK expression				
Low	Reference		Reference	
High	1.985 (1.031−3.822)	**0.040**	2.193 (1.133−4.245)	**0.020**

*HR* hazard ratio, *CI* confidence interval.

^a^The *P* value was adjusted by the univariate Cox proportional hazard regression model.

*P* value less than 0.05 were bolded.

**Table 2:** Univariate and multivariate cox proportional hazard model for overall survival (OS) for TNBC patients.

Characteristics	Univariate analysis		Multivariate analysis	
	Hazard ratio (95% CI)	*P* value	Hazard ratio (95% CI)	*P*^*a*^ value
Age (years)				
≤50	Reference			
>50	0.950 (0.484−1.864)	0.881		
Tumor size				
≤2 cm	Reference			
>2 cm	1.605 (0.699−3.687)	0.265		
Lymph nodes metastasis				
No	Reference		Reference	
Yes	3.298 (1.573−6.915)	**0.002**	3.382 (1.611−7.100)	**0.001**
Grade				
I + II	Reference			
III	0.655 (0.324−1.326)	0.240		
BCKDK expression				
Low	Reference		Reference	
High	1.995 (1.005−3.962)	**0.048**	2.088 (1.048−4.159)	**0.036**

*HR* hazard ratio, *CI* confidence interval.

^a^The *P* value was adjusted by the univariate Cox proportional hazard regression model.

*P* value less than 0.05 were bolded.

### Contribution of BCKDK to breast cancer (BC) growth

**Fig. 2:**
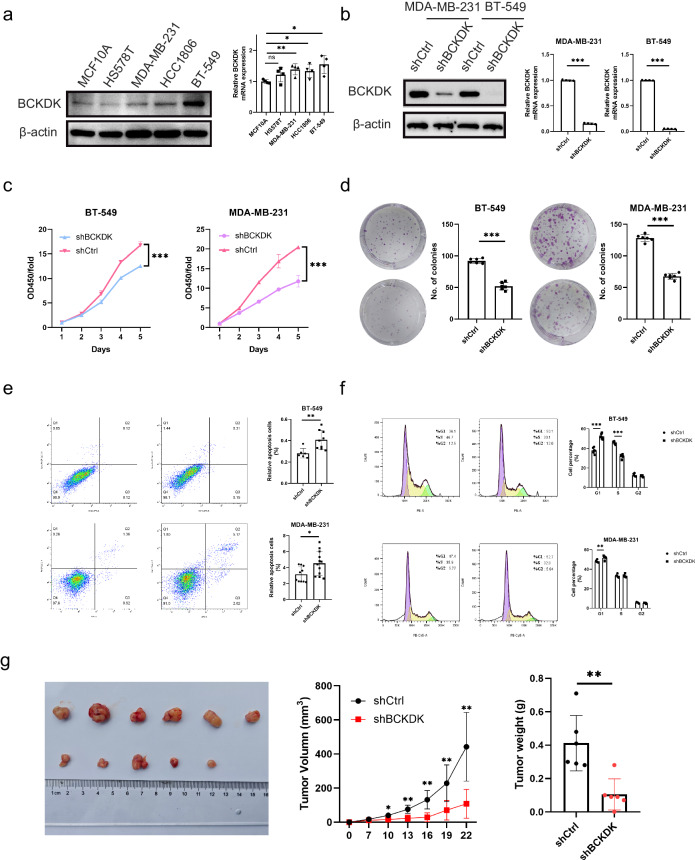
Suppression of tumorigenesis by BCKDK knockdown. **a** BCKDK expression in different TNBC cell lines and MCF10A. **b** Efficiency of shBCKDK assessed using western blotting and qRT-PCR in MDA-MB-231 and BT-549 cells. **c** Growth curves, **d** Colony formation assay, **e** Cell apoptosis, **f** Cell cycle assays of shBCKDK and shCtrl cells. **g** BCKDK knockdown diminishes tumorigenic properties of BT-549 cells in vivo. *P < 0.05, **P < 0.01, ***P < 0.001.

BCKDK expression was evaluated across various TNBC cell lines, revealing elevated levels compared with that in the normal breast epithelial cell line (MCF10A) (Fig. 2a). MDA-MB-231 and BT549 cell lines were selected for further investigation, with shBCKDK-1 demonstrating superior interference efficiency among the constructed shRNAs. This selection was confirmed using qRT-PCR and western blotting (Fig. 2b). Subsequent CCK-8 and colony formation assays revealed that BCKDK knockdown significantly hindered cell proliferation and colony formation in both MDA-MB-231 and BT549 cell lines. Flow cytometry analysis further indicated an increase in cell apoptosis, a decreased proportion of S-phase cells, and an increased proportion of G1 phase cells following BCKDK knockdown (Fig. 2c–f).

To assess the role of BCKDK in promoting TNBC growth in vivo, a preclinical xenograft model was established by subcutaneously implanting BT-549 TNBC cells with or without BCKDK knockdown into BALB/c nude mice. Consistent with in vitro findings, BCKDK depletion led to attenuated tumour growth in vivo (Fig. 2g). Furthermore, the overexpression model demonstrated that BCKDK overexpression significantly increased the proliferative capacity of TNBC cells (Fig. S1).

Collectively, the up-regulation of BCKDK expression contributes to TNBC growth by promoting cell cycle progression and reducing apoptosis.

### Influence of BCKDK on BC cell functions through metabolic reprogramming

While BCKDK plays a crucial role in BCAA degradation, direct BCAA supplementation at the cellular level failed to enhance TNBC cell proliferation, both in normal and BCKDK-knockdown cells (Fig. S2a, b). This suggests that BCKDK exerts its function in BCAAs-independent way in TNBC cells.

Previous research results suggest that BCKDK is localised in mitochondria, which are important regulators of cellular energy and metabolism and play a crucial role in cell survival and proliferation. Therefore, the morphology of mitochondria was observed by transmission electron microscopy and changes in mitochondrial membrane potential were detected to assess mitochondrial function. Electron microscopy revealed no significant alterations in the mitochondrial morphology between knockdown and control groups (Fig. S2c, d). Nevertheless, an increase in mitochondrial membrane potential and ROS were observed following BCKDK knockdown (Fig. S2e, f).

**Fig. 3:**
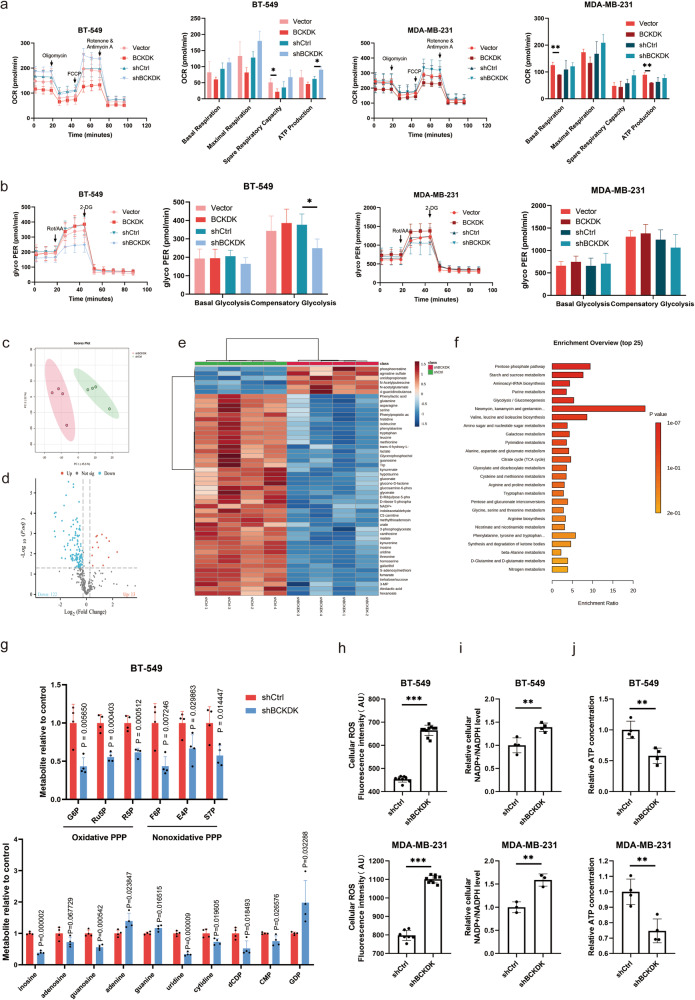
Metabolic alterations induced by BCKDK. **a**, **b** Oxygen consumption rate (OCR) and extracellular acidification rate (ECAR) as a measure of BCKDK-induced metabolic changes in MDA-MB-231 and BT-549 cells. **c** Clustering of shBCKDK samples separately from shCtrl groups. **d** Volcano plots, **e** Heat map of differentially expressed metabolites between shBCKDK and shCtrl groups in BT-549 cells. **f** Metabolite enrichment analysis of differential metabolites between shBCKDK and shCtrl BT-549 cells. **g** Metabolite changes of oxidative PPP, nonoxidative PPP, and nucleotides in shBCKDK and shCtrl BT-549 cells. **h** Cellular ROS, **i** Relative NADP^+^/NADPH ratios, **j** Relative ATP concentration in shBCKDK and shCtrl BT-549 and MDA-MB-231 cells. *P < 0.05, **P < 0.01, ***P < 0.001.

Mitochondrial membrane potential is closely related to the energy-producing pathways and is used by ATP synthase to produce ATP as an intermediate form of energy storage. Glycolysis and the oxidative respiration pathway are pivotal energy-producing pathways in tumour cells. We therefore used OCR and ECAR to measure oxidative phosphorylation and glycolysis, respectively. The results showed that BCKDK knockdown led to an increase in OCR and a decrease in ECAR; however, these changes were not statistically significant (Fig. 3a, b). To investigate the mechanisms that govern the function of BCKDK in TNBC cells, a metabolome analysis was conducted, revealing enrichment in pathways such as BCAA biosynthesis, the PPP, and glycolysis/gluconeogenesis (Fig. 3c–f). Oxidative PPP metabolites 6PG (6-phosphogluconic acid) and R5P (ribose-5-phosphate), and non-oxidative metabolites S7P (sedoheptulose-7-phosphate) and E4P (erythrose-4-phosphate), were significantly decreased in the BCKDK knockdown group as well as nucleotides (Fig. 3g). Subsequent examination of cellular NADP^+^/NADPH and ROS levels indicated that silencing BCKDK elevated the NADP^+^/NADPH ratio and increased ROS accumulation, indicating intensified oxidative stress damage (Fig. 3h, i). Relative ATP concentration was also decreased with BCKDK knockdown (Fig. 3j).

To further confirm metabolic changes, [U-^13^C6] glucose and [U-^13^C6] leucine were individually utilised to track metabolic flux in shBCKDK and shCtrl BT-549 cells. Metabolites from glycolysis and PPP decreased in the shBCKDK group (Fig. S3), while BCAA and the tricarboxylic acid (TCA) cycle significantly increased indicating a new model of metabolic reprogramming in TNBC with the loss of BCKDK (Fig. S4).

### *G6PD* identified as a target gene for BCKDK

**Fig. 4:**
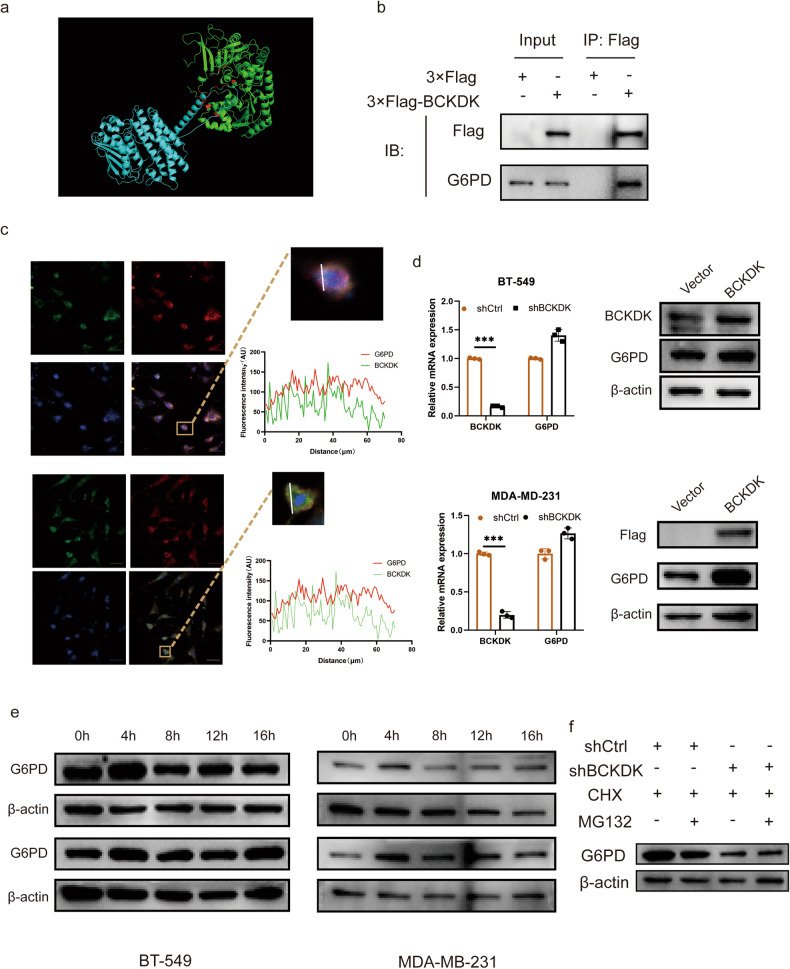
Interaction between BCKDK and G6PD influencing protein stability. **a** Interaction model of BCKDK (blue) and G6PD (green). Red indicates interaction sites. **b** Co-immunoprecipitation of BCKDK with G6PD. **c** Representative images of green fluorescence from BCKDK co-localised with red fluorescence from G6PD. **d** BCKDK did not substantially affect *G6PD* RNA expression but positively influenced protein expression. **e** G6PD degradation analysis over different time points between BCKDK (lower) and Vector (upper) groups in BT-549 and MDA-MB-231 cells. **f** MG132, a proteasome inhibitor, did not affect the degradation of G6PD in the shBCKDK and shCtrl groups.

HK2 plays a crucial role in glycolysis and the PPP by phosphorylating glucose to glucose-6-phosphate. However, although the expression of HK2 protein decreased after BCKDK knockdown, BCKDK protein was not detected in immunoprecipitation of cell lysate with HK2 antibody (Fig. S5). We hypothesised that G6PD plays a pivotal role in TNBC metabolic reprogramming as the rate-limiting enzyme for PPP. To validate this hypothesis, an interaction model demonstrating the strong protein–protein interaction between BCKDK and G6PD was constructed (Fig. 4a, Interaction confidence analysis: 0.874). Specific binding sites are detailed in Table S4. IP-MS was performed in BT-549 stable cells expressing 3×Flag-tagged BCKDK overexpression/vector cells. Co-IP experiments in BT-549 cells overexpressing BCKDK further validated the robust binding between BCKDK and G6PD (Fig. 4b). Immunofluorescence co-localisation analysis revealed partial co-localisation of G6PD (red) with BCKDK (green) in the cytosolic fraction (Fig. 4c).

Next, we examined whether BCKDK modulates the mRNA or protein levels of G6PD through their interaction. Results indicated that BCKDK overexpression increased G6PD protein levels without altering *G6PD* mRNA levels (Fig. 4d). Additionally, investigations into whether BCKDK enhances the stability of G6PD protein revealed that BCKDK overexpression weakened G6PD degradation compared with that in the control group (Fig. 4e). The proteasomal pathway, the primary pathway for intracellular protein degradation, was explored using the proteasome inhibitor MG132. Our findings suggested that G6PD degradation occurred through a proteasome-independent pathway (10-h treatment, Fig. 4f). These findings suggest an alternative pathway for G6PD degradation.

### BCKDK enhances cell proliferation via G6PD through mTOR signalling

**Fig. 5:**
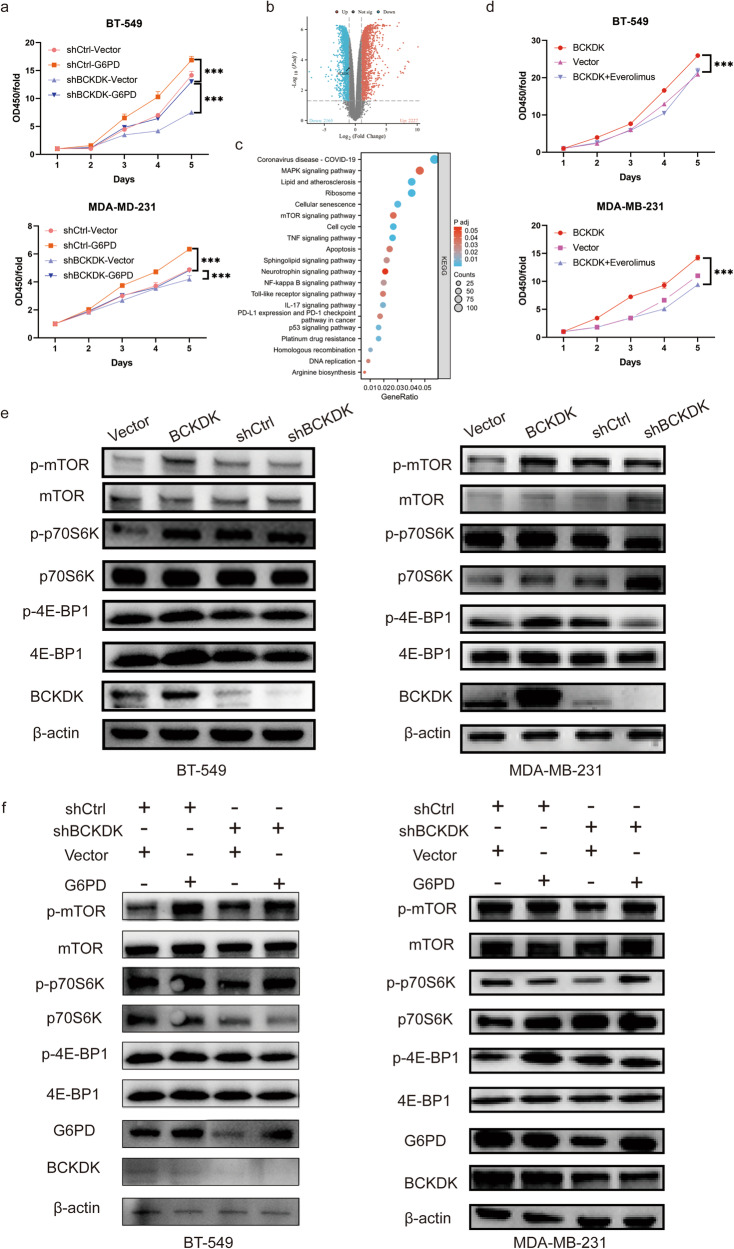
Knockdown of BCKDK suppresses tumorigenesis. **a** BCKDK knockdown-mediated inhibition in proliferation was rescued by G6PD. **b** Volcano of differentially expressed genes between shBCKDK and shCtrl BT-549 cells. **c** Kyoto Encyclopedia of Genes and Genomes (KEGG) enrichment analysis of differential metabolites between shBCKDK and shCtrl cells of BT-549. **d** BCKDK overexpression-induced proliferation reversed by everolimus. **e** Changes in key proteins of the mTOR signalling pathway in differentially expressed BCKDK levels. **f** Changes in key proteins of the mTOR signalling pathway were analysed by western blot after BCKDK knockdown and G6PD overexpression. ***P < 0.001.

Given the significant impact of BCKDK on tumour proliferation, we hypothesised that BCKDK plays a proliferative role through G6PD. Transfection of G6PD overexpression plasmids into stable BCKDK knockdown BT-549 and MDA-MB-231 cells demonstrated that G6PD restored proliferation inhibited by BCKDK knockdown (Fig. 5a).

The mTOR signalling pathway, known for its central role in metabolism regulation, was further explored. KEGG pathway enrichment analysis of differentially expressed genes obtained using RNA-seq analysis revealed enhanced mTOR signalling between the knockdown and control groups (Fig. 5b, c). BCKDK up-regulation resulted in increased protein levels of p-mTOR^S2448^, p-ribosomal protein S6 kinase (p70S6K)^T389^, and p-eukaryotic translation initiation factor 4E (eIF-4E)-binding protein 1 (4E-BP1)^S65^. Everolimus, an mTOR inhibitor, effectively inhibited cell proliferation induced by BCKDK overexpression (Fig. 5d). Results from western blotting showed that BCKDK activated mTOR signalling (Fig. 5e).The down-regulated expression of p-mTOR, p-p70S6K and p-4E-BP1 proteins induced by BCKDK knockdown can be reversed by overexpression of G6PD (Fig. 5f).

Collectively, our data suggest that BCKDK upregulates G6PD expression by increasing the stability of the G6PD protein, thereby exerting its proliferation ability through mTOR signalling.

### MAZ induces BCKDK transcription in TNBC

**Fig. 6:**
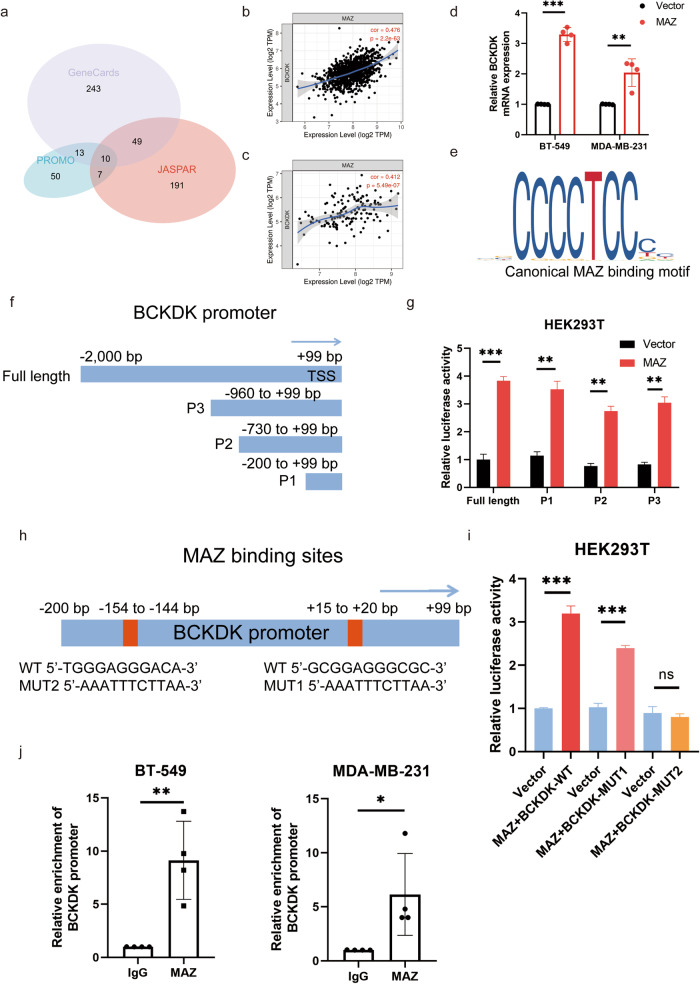
MAZ activates BCKDK transcription by direct binding to its promoter. **a** Venn diagram of upstream transcription factor of BCKDK based on GeneCards, promo, and JASPAR datasets. mRNA expression of BCKDK and MAZ was evaluated using TIMER online analysis of BC (**b**) and TNBC (**c**) tissues. **d** *BCKDK* mRNA levels with MAZ overexpression. **e** Schematic diagram of canonical MAZ-binding motif (JASPAR Database). **f** Schematic representation of BCKDK promoter fragments. TSS is the transcriptional start site of BCKDK. (**g)** Luciferase assay was used to detect BCKDK promoter transcriptional activity. **h**, **i** Luciferase activity of BCKDK promoter (WT or MUT) was detected after MAZ overexpression in HEK-293T cells. **j** ChIP assays confirmed that MAZ is bound to the BCKDK promoter. IgG served as a negative control. *P < 0.05, **P < 0.01, ***P < 0.001.

After intersecting JASPAR, GeneCards, and PROMO databases, ten candidates, including *PAX5*, *LEF1*, *MYC*, *ETS1*, *ELK1*, *SP1*, *MAZ*, *TBP*, *ATF2*, and *HNF1A*, were selected for further analysis (Fig. 6a and Fig. S6). MAZ was predicted to be the most likely candidate using TIMER, which was utilised to investigate the expression correlation in BC and TNBC (Fig. 6b, c).

Results from western blotting and qRT-PCR confirmed that overexpression of MAZ increased BCKDK expression in TNBC cells (Fig. 6d). The canonical MAZ-binding motif is depicted in Fig. 6e. We designed three BCKDK promoter fragments, named P1 to P3 (−200 to +99 bp, −730 to +99 bp, and −960 to +99 bp; Fig. 6f). Using a luciferase reporter construct, the relative luciferase activity of BCKDK (−2000 to +99 bp) significantly increased after transfection with the MAZ overexpression plasmid. The relative luciferase activity of each fragment demonstrated significant changes after co-transfection (Fig. 6g). Subsequently, specific MAZ-binding sites on the BCKDK promoter were predicted (Fig. 6h). Thus, two predicted binding sites (+15, GCGGAGGGCGC and −154, TGGGAGGGACA) were selected, and the enhanced luciferase activity was reversed by transfection of the “TGGGAGGGACA”-mutated BCKDK sequence (Fig. 6i). Subsequently, ChIP assays confirmed that MAZ was significantly enriched in the BCKDK promoter region in TNBC cells (Fig. 6j). Collectively, the transcription factor MAZ binds to the BCKDK promoter region and promotes its transcription.

### BT2 inhibits TNBC growth in the PDX model

**Fig. 7:**
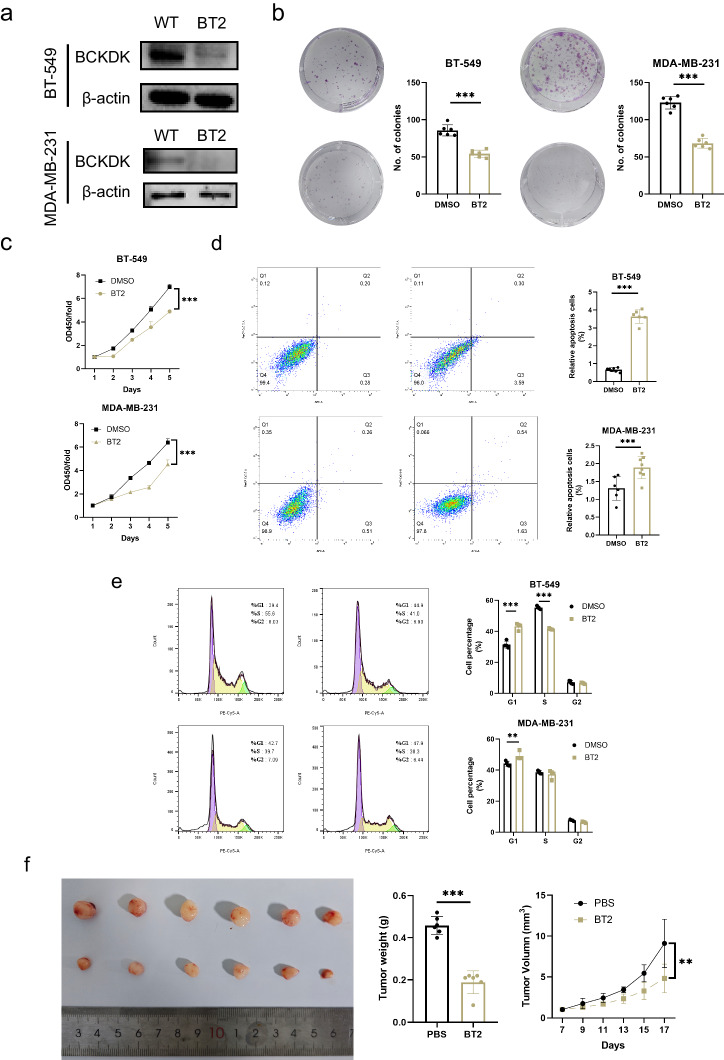
BT2 suppresses tumour growth in vitro and in vivo. **a** Inhibition efficiency under BT2 treatment assessed via western blotting in MDA-MB-231 and BT-549 cells. **b** Growth curves, **c** Colony formation assay, **d** Apoptosis, **e** Cell cycle assay results of MDA-MB-231 and BT-549 cells under BT2 treatment. **f** BT2 inhibited TNBC growth in the patient-derived tumour xenograft model. *P < 0.05, **P < 0.01, ***P < 0.001.

To further validate our results, BCKDK inhibitor, BT2, was employed to determine the role of BCKDK in TNBC both in vitro and in vivo. IC50 curves were fitted by non-linear regression and used to determine the IC50 concentrations. Log-logistic showed that the median lethal dose was 165.9 µM (161.9 µm-170.0 µM), so a BT2 concentration of 100 µM was chosen as the working concentration for subsequent experiments (Fig. S7a). Similar to BCKDK knockdown, BT2 treatment inhibited TNBC growth, increased the number of apoptosis cells and blocked G1-S cell cycle (Fig. 7a–e). Metabolite changes induced by BT2 were shown in Fig. S7b–e.

PDX models are regarded as accurate and reliable preclinical models because they closely mimic the clinical characteristics of original human cancer [26]. BT2 exerted a comparable effect on the PDX model and demonstrated a marked anti-tumour effect. indicating the immense potential of BCKDK activity inhibition in clinical therapeutic development (Fig. 7f).

## Discussion

One hallmark of cancer is metabolic reprogramming; however, its contribution to the survival and proliferation of TNBC cells requires further investigation. Our findings suggested that BCKDK enhanced PPP flux by interacting with G6PD. This interaction helped meet the bioenergetics and biosynthetic demands and decreased the NADP/NADPH ratio and ROS accumulation, minimising oxidative stress damage in TNBC. The BCKDK inhibitor, BT2, exerted its anti-proliferation effect both in vitro and in vivo.

BCKDK, the key enzyme of BCAA metabolism, is reportedly highly expressed in colorectal [27], liver [28], lung [29], breast [30], and ovarian [9] cancers. Additionally, BCKDK is associated with resistance to certain chemotherapies, including doxorubicin and paclitaxel, and radiotherapy resistance [31, 32]. BCKDK knockdown increased oxidative phosphorylation as measured using OCR. One possible explanation for this is that BCKDK phosphorylates branched-chain keto acid dehydrogenase complex subunit alpha (BCKDHA) to inhibit its activity, leading to the accumulation of branched-chain keto acid (BCKA) and subsequently causing a decrease in the TCA cycle and mitochondrial oxidative phosphorylation. However, increased mitochondrial function is typically related to the promotion of cell proliferation. Therefore, we hypothesised that other metabolic pathways may be involved.

BCKDK resides in the mitochondrial fraction; however, a recent study demonstrated that BCKDK was preferentially localised in the cytoplasm as well as mitochondrial subcellular compartment [33]. Although BCKDK reportedly increases ATP citrate lyase (ACL) phosphorylation and de novo lipogenesis, its role in regulating glucose metabolism remains less explored. Our findings revealed that BCKDK increased the stability of G6PD. Thus, elevated PPP can provide essential biosynthetic intermediates to the tumour, facilitating tumour growth and promoting chemotherapy drug-induced DNA damage repair. Additionally, PPP can protect tumour cells from ionising radiation or chemotherapy drugs by using NADPH as a scavenger of intracellular ROS released by these treatments. Therefore, targeting BCKDK-dependent induction of PPP may contribute to the proliferation of tumour cells sensitive to chemotherapy and/or radiotherapy. A previous study reported that BCKDK could be phosphorylated by Src, thereby promoting metastasis of colorectal cancer. EGF also can regulate the activity of BCKDK [27]. In this study, we observed that MAZ activated the transcription of BCKDK.

G6PD catalyses a critical reaction in the PPP as a rate-limiting enzyme. G6PD inhibitors, such as 6-aminonicotinamide and dehydroepiandrosterone, have been widely used in many cancers [34–37]. p52-ZER6 was previously reported to enhance *G6PD* activity as a transcriptional factor in estrogen receptor positive BC cells regardless of *TP53* mutation [38]. Suppressed expression of 4-hydroxyphenylpyruvate dioxygenase (HPD) decreases oxidative PPP flux, and HPD is a key enzyme involved in tyrosine catabolism, which catalyses the conversion of 4-hydroxyphenylpyruvate into homogentisate. HPD contributes to G6PD expression, thus enhancing the PPP flux and facilitating lung cancer growth [39].

By activating classical pathways such as the MEK/ERK and MAPK signal pathway, BCKDK promotes tumorigenesis and metastasis [9, 40]. In this study, the proliferation of BCKDK in TNBC was suppressed by the mTOR signalling pathway inhibitor, everolimus. The mTOR signalling pathway is associated with BCAA metabolism, lipid metabolism, and glucose metabolism [41–43]. In PPP, mTORC1-activated HIF1α and SREBP transcription could enhance *G6PD* mRNA expression [44]. mTOR inhibitors promote G6PD autophagic degradation, thus affecting PPP [45]. Androgen receptor signalling reportedly promotes the PPP through mTOR-mediated up-regulation of G6PD in prostate cancer [46]. Therefore, further investigation of the mechanism by which BCKDK interacts with G6PD and whether mTOR signalling pathways function cooperatively or translocation warrants further exploration. This is the shortcoming of our study.

Our findings highlight the significance of BCKDK in TNBC metabolism, shedding light on potential therapeutic targets for intervention. The identification of the BCKDK-G6PD interaction and its impact on PPP flux opens avenues for targeted drug development. Moreover, the observed sensitivity of TNBC cells to the BCKDK inhibitor, BT2, suggests its potential as a promising candidate for further preclinical and clinical exploration. This research lays the groundwork for future studies aiming to unravel the complexities of metabolic reprogramming in TNBC and holds promise for the development of innovative treatment strategies.

### Supplementary information

Supplementary data

Highlights

## Data Availability

Core data has been provided in the main text. The datasets used and/or analysed during the current study are available from the corresponding author on reasonable request.
