# Supplementary material for: MAZ-mediated up-regulation of BCKDK reprograms glucose metabolism and promotes growth by regulating glucose-6-phosphate dehydrogenase stability in triple-negative breast cancer
Source: Cell Death Dis. 2024 Jul 18;15(7):516. doi: 10.1038/s41419-024-06835-y (PMC11258276; doi:10.1038/s41419-024-06835-y)
Supplement: Supplementary file 1 — Supplementary data [file 41419_2024_6835_MOESM1_ESM.pdf]

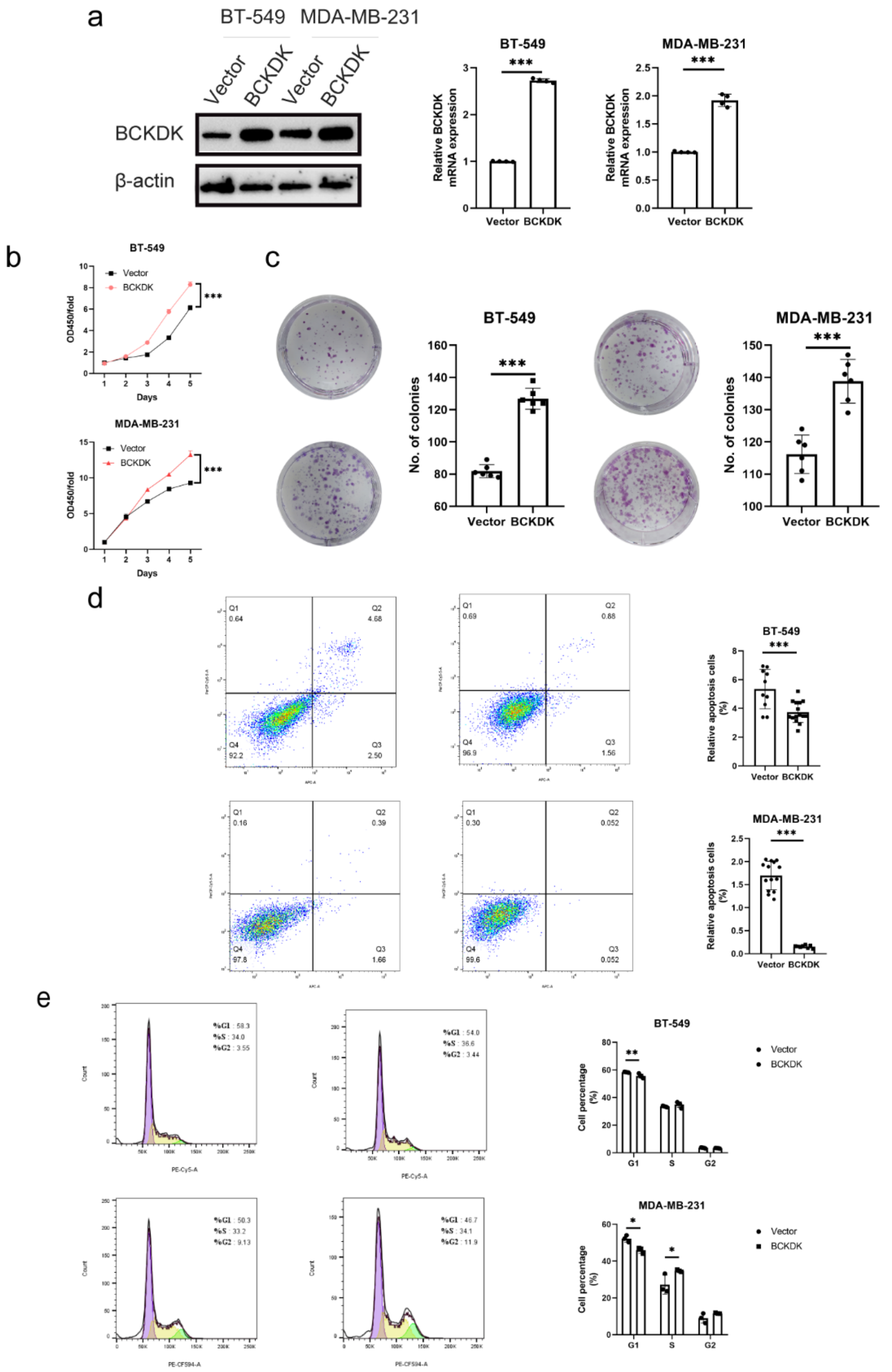

Fig. S1. Overexpression of BCKDK promotes tumorigenesis. **(a)** Overexpression efficiency of BCKDK assessed via western blotting and qRT-PCR in MDA-MB-231 and BT-549 cells. **(b)** Growth curves, **(c)** colony formation assay, **(d)** apoptosis, and **(e)**

cell cycle assay results from BCKDK and empty vector cells. * $P<0.05$, ** $P<0.01$, *** $P<0.001$.

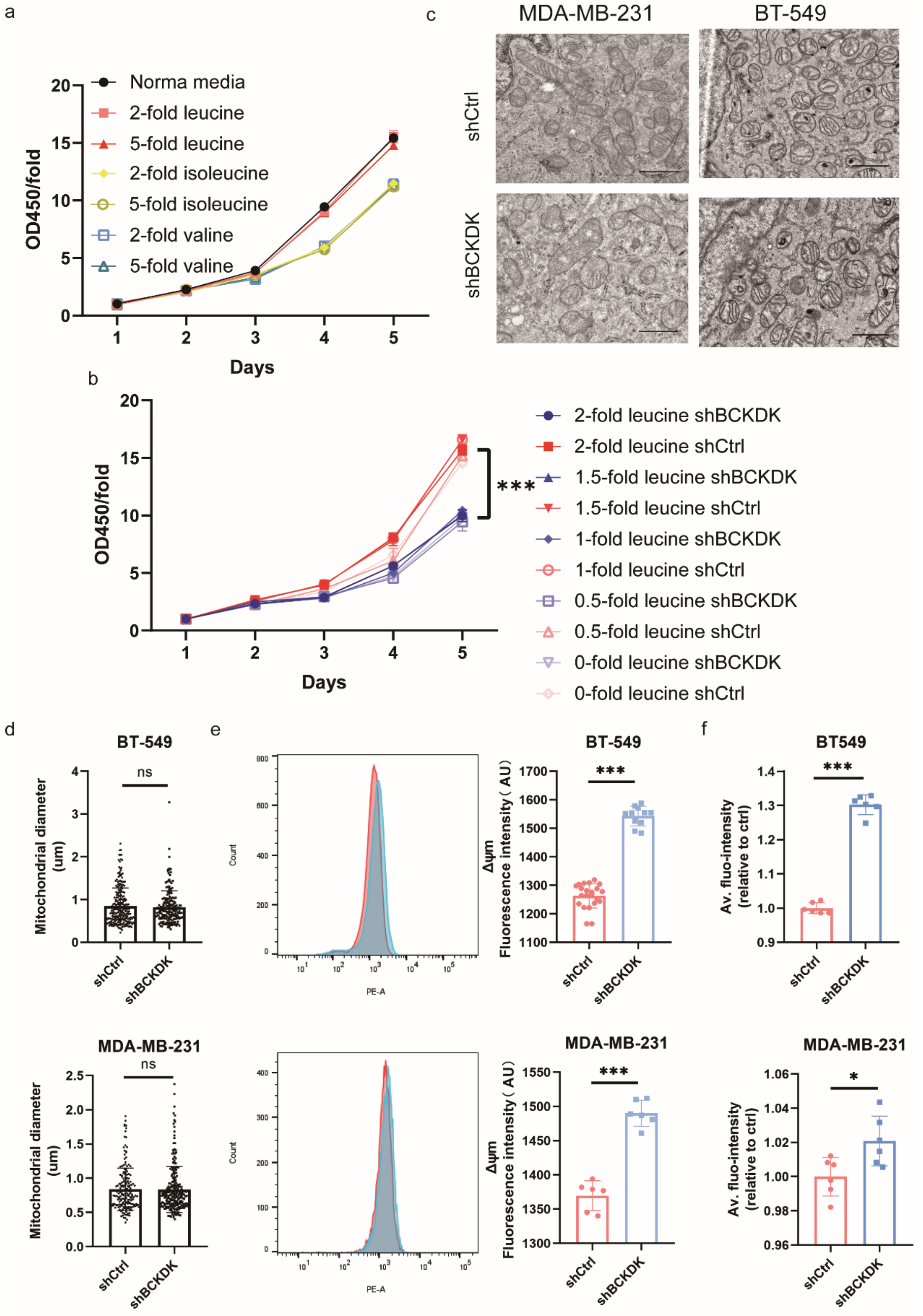

Fig. S2. BCAAs accumulation fails to promote proliferation and changes in mitochondria of TNBC cells. **(a)** Growth curves of BT-549 cells at different BCAA concentrations. **(b)** Growth curves of BCKDK knockdown BT-549 cells at different leucine concentrations. **(c)** Representative electron microscopic images, **(d)** the diameter of mitochondria, **(e)** mitochondrial membrane potential (Δψm), and **(f)** mitochondrial ROS of shBCKDK and shCtrl BT-549 and MDA-MB-231 cells. * *P*<0.05, ** *P*<0.01, *** *P*<0.001.

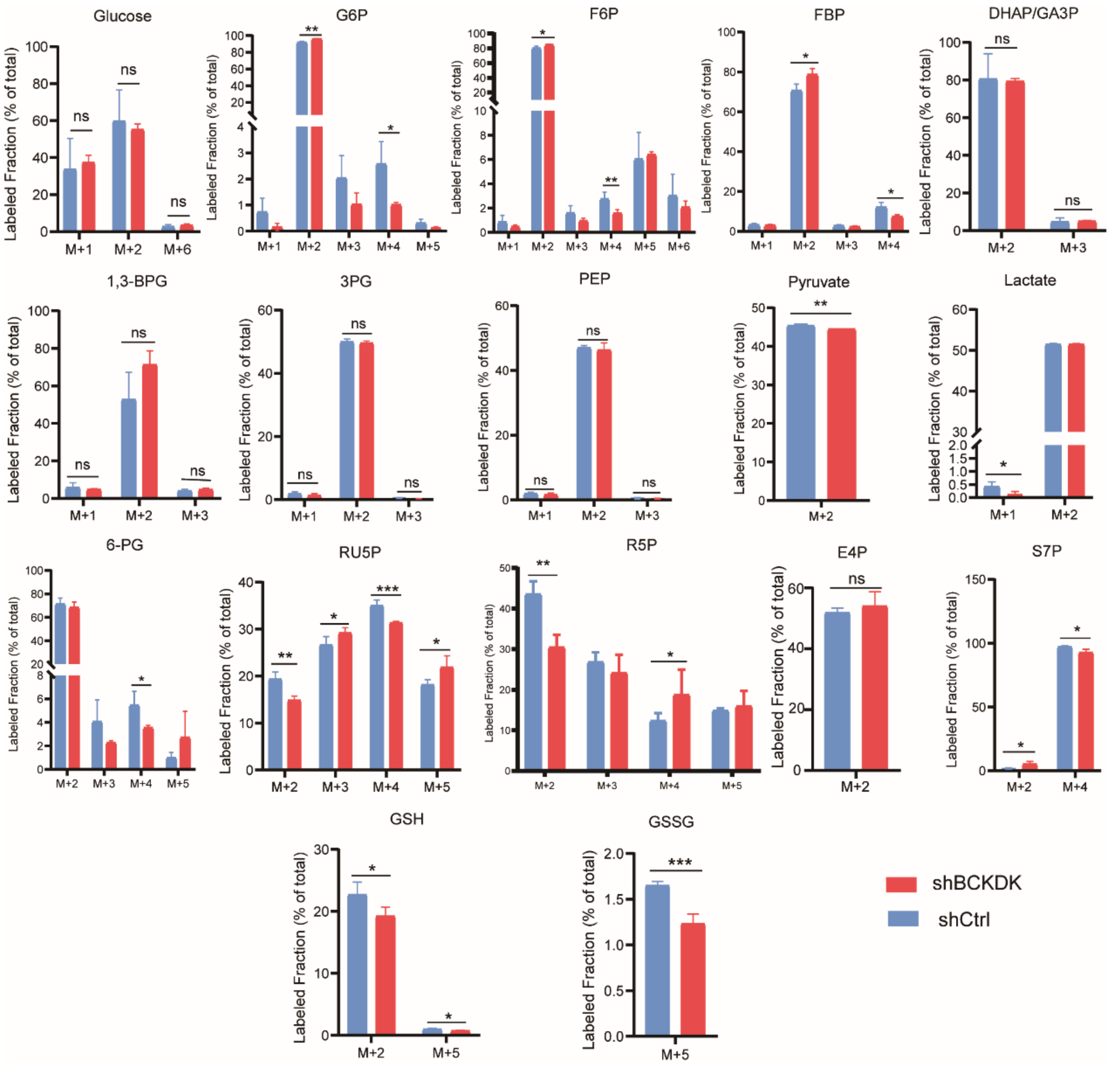

Fig. S3. [U-[13]C6] glucose metabolic flux alteration in shBCKDK BT-549 cells compared with that in the shCtrl group. Changes in metabolites from glycolysis and the

pentose phosphate pathway (PPP). m+1 and m+2 indicate the number of labelled 13C. * $P<0.05$, ** $P<0.01$, *** $P<0.001$.

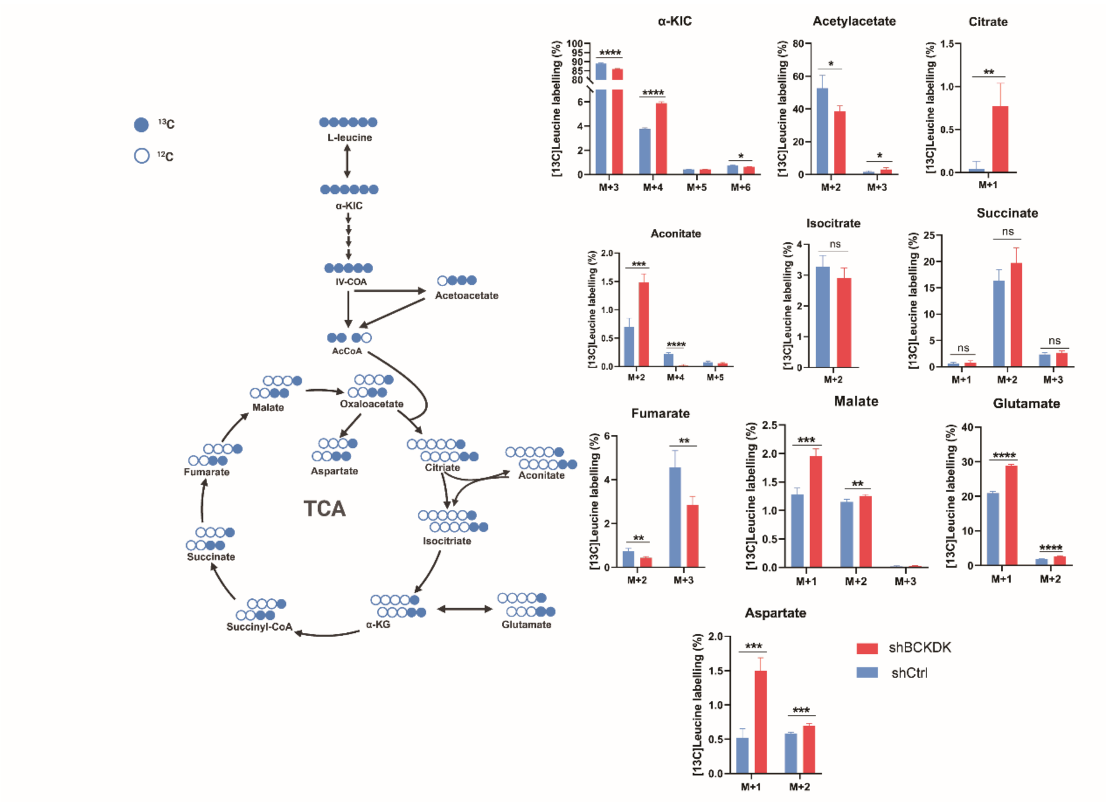

Fig. S4. [U-^{13}C6] leucine metabolic flux alteration in shBCKDK BT-549 cells compared with that in the shCtrl group. Changes in metabolites from branched chain amino acid (BCAA) and the tricarboxylic acid (TCA) cycle. m+1 and m+2 indicate the number of labelled ^{13}C. * $P<0.05$, ** $P<0.01$, *** $P<0.001$.

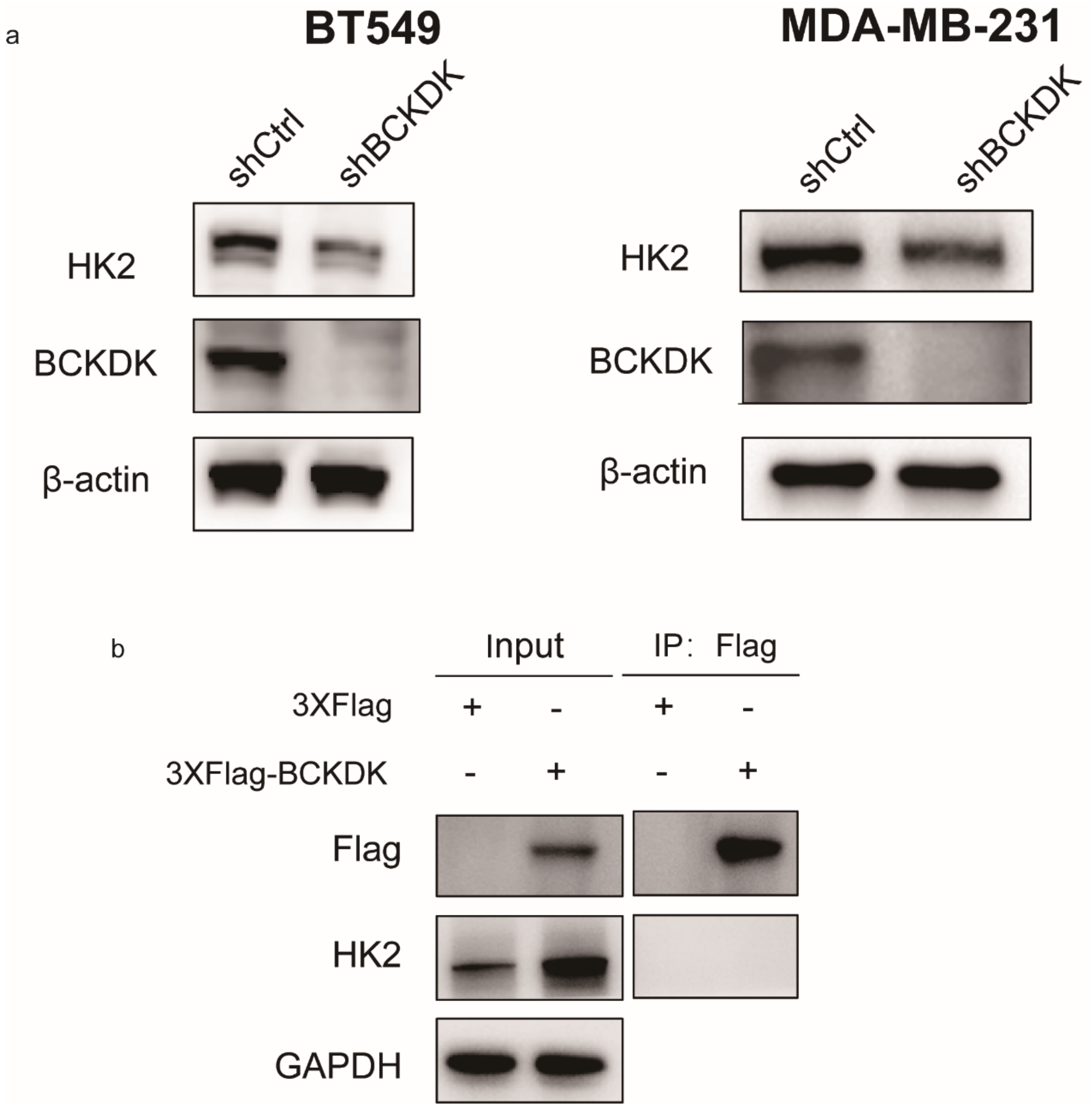

Fig. S5. Interaction between BCKDK and HK2 protein. (**a)** The expression of HK2 protein decreased after BCKDK knockdown. (**b)** Co-immunoprecipitation of BCKDK with HK2.

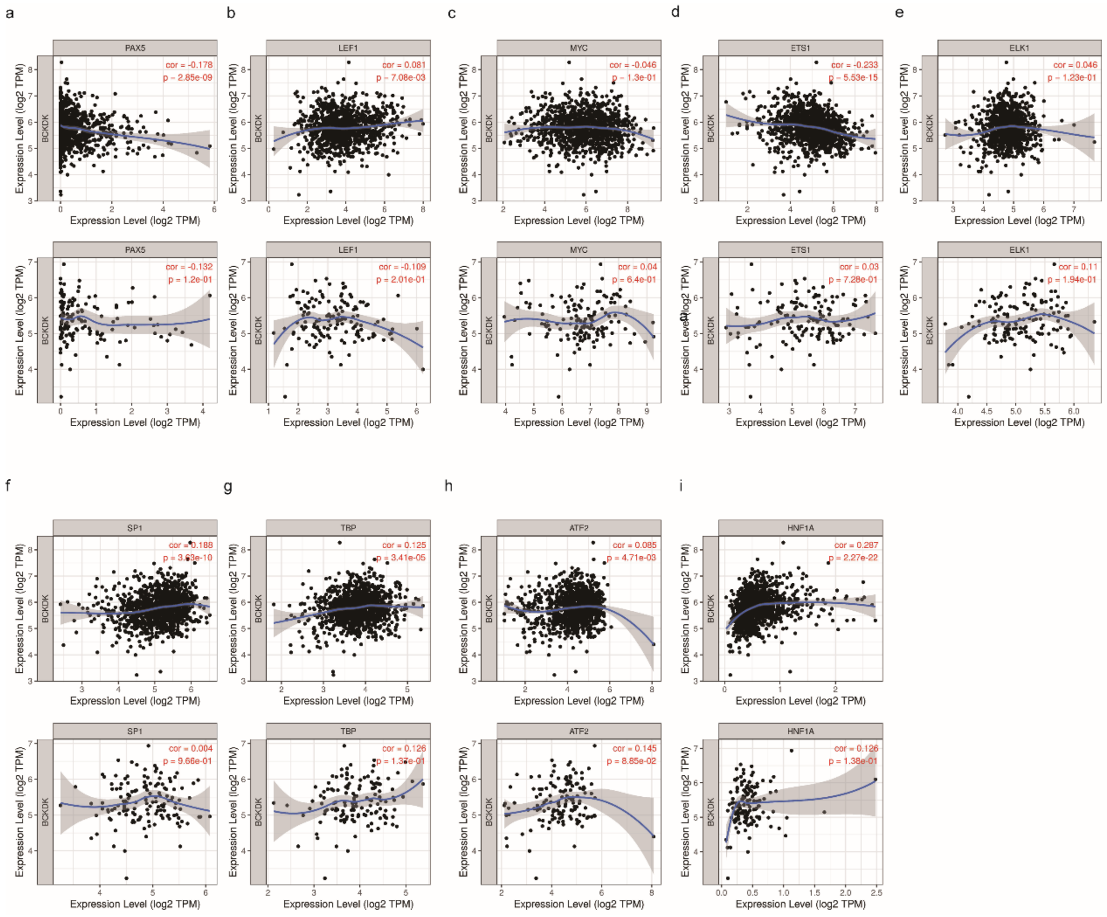

Fig. S6. Correlation between potential upstream transcription factors and BCKDK mRNA assessed through TIMER online analysis. The upper panels illustrate plots from BC samples, and the lower panels depict plots from TNBC samples. **(a)** *PAX5*. **(b)** *LEF1*. **(c)** *MYC*. **(d)** *ETS1*. **(e)** *ELK1*. **(f)** *SP1*. **(g)** *TBP*. **(h)** *ATF2*. **(i)** *HNF1A*.

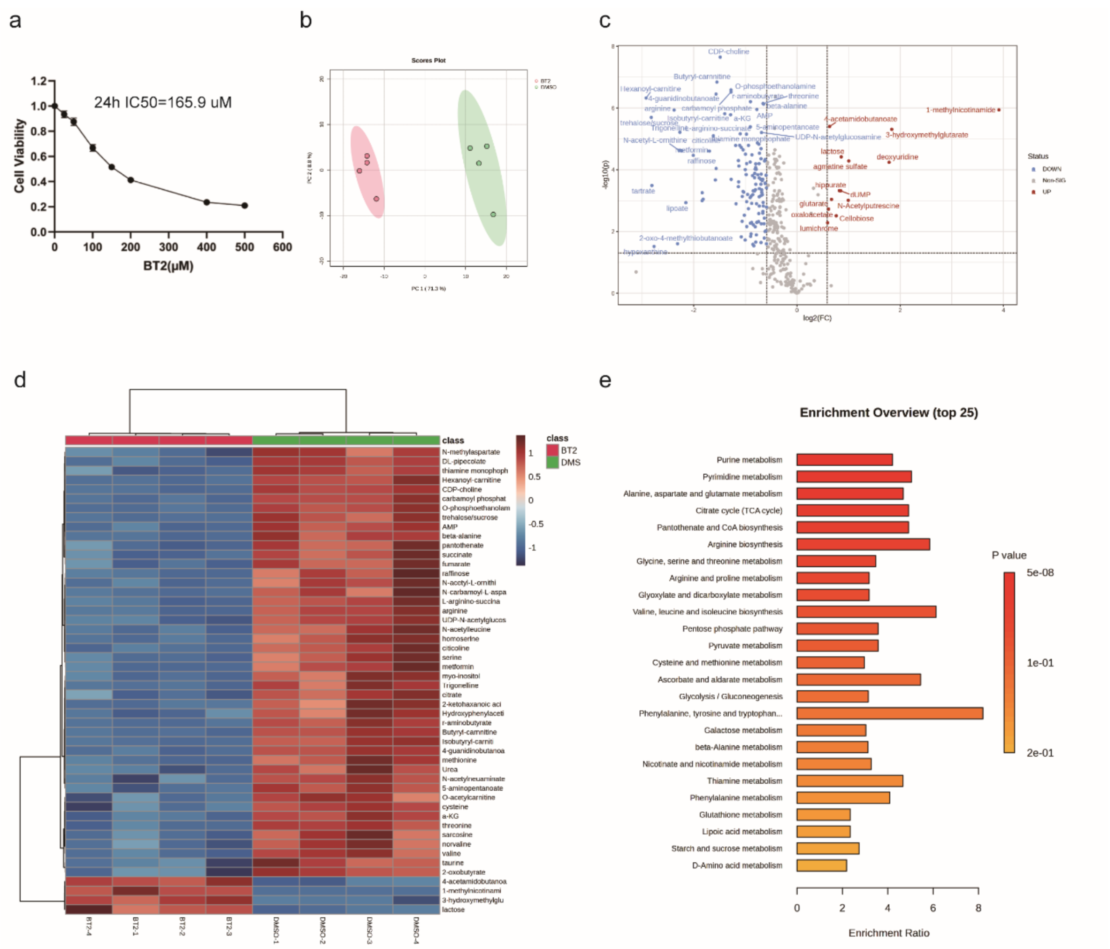

Fig. S7. Metabolic alterations induced by BT2. (**a**) Growth-inhibition curve of BT2. (**b**) Clustering of BT2 samples separately from DMSO groups. (**c**) Volcano plots, (**d**) Heat map of differentially expressed metabolites between BT2 and DMSO groups in BT-549 cells. (**e**) Metabolite enrichment analysis of differential metabolites between BT2 and DMSO BT-549 cells.

Table S1. The sequences of shRNAs used in this study.

Name	Sequence
shBCKDK-1	5’-ACGCTGACTTCGAGGCTTGGA-3’
shBCKDK-2	5’-GACAGACTTCCCTCCGATCAA-3’
shBCKDK-3	5’-AGAAGCCCTCAGTCCGCCTAA-3’
shCtrl	5’-TTCTCCGAACGTGTCACGT-3’

Table S2. Gene-specific primers used for qRT–PCR.

Genes	Sequences (5′→3′)
BCKDK	Forward: GTGAGAGCCGGAAGCACATA<br>Reverse: TGGCAAAGTCCACCCACTTC
MAZ	Forward: CGCACACAGTACGACACGAG<br>Reverse: GAGCTCACAGACATGGTGAGG
BCKDK-chip-qpcr1	Forward: CGTCCTCAGACCAAACTACAACT<br>Reverse: GAAGGGCTCGAACAGTCCAC
BCKDK-chip-qpcr2	Forward: CCGTTTCATAGAGCAGGAGATAGA<br>Reverse: TGGGAGTTGTAGTTTGGTCTGA
G6PD	Forward: ACCGCATCGACCACTACCT<br>Reverse: TGGGGCCGAAGATCCTGTT
β-actin	Forward: CATGTACGTTGCTATCCAGGC<br>Reverse: CTCCTTAATGTCACGCACGAT

Table S3. Information on the primary and secondary antibodies used in this study.

Antibody	Vendor	Cat#	Hosts	Working concentration
BCKDK	Abcam	ab151297	R	1:5000 (WB)<br>1:400 (IHC)
BCKDK	santa cruz	sc-374424	M	1:1000 (WB)<br>1:50 (IF)
mTOR	proteintech	66888-1-Ig	M	1:10000
p-mTOR (S2448)	proteintech	67778-1-Ig	M	1:10000
p70S6K	CST	#9202	R	1:1000
p-p70S6K (T389)	CST	#97596	R	1:1000
4E-BP1	CST	#9452	R	1:1000
p-4E-BP1 (S65)	CST	#9451	R	1:1000
β-actin	Beyotime Biotechnology	AA128	M	1:1000
G6PD	ABclonal	A1537	R	1:500 (WB)<br>1:50 (IF)
Flag	Selleck	A5712	M	1:2000

M, mouse; R, rabbit; G, goat; CST, Cell Signaling Technology; HRP, horseradish peroxidase.

Table S4. Predicted specific binding sites between G6PD and BCKDK.

##	G6PD	Dist.[A]	BCKDK	Type
1	A:ARG 57[NH2]	2.99	B:ASP 36[OD2]	Hydrogen bonds
2	A:TYR 202[OH]	3.86	B:ARG 28[NH1]	Hydrogen bonds
3	A:GLU 398[OE1]	2.26	B:ARG 30[NH1]	Hydrogen bonds
4	A:VAL 394[O]	3.12	B:ARG 30[NH2]	Hydrogen bonds
5	A:ASN 430[O]	3.14	B:HIS 39[NE2]	Hydrogen bonds
6	A:GLN 11[OE1]	2.56	B:ARG 46[NH1]	Hydrogen bonds
7	A:ALA 6[O]	2.89	B:ARG 46[NH2]	Hydrogen bonds
8	A:ARG 9[NH1]	2.52	B:ASP 36[OD1]	Salt bridges
9	A:ARG 9[NE]	3.35	B:ASP 36[OD1]	Salt bridges
10	A:ARG 9[NH2]	2.60	B:ASP 36[OD1]	Salt bridges
11	A:ARG 9[NH1]	3.46	B:ASP 36[OD2]	Salt bridges
12	A:ARG 57[NH1]	3.61	B:ASP 36[OD2]	Salt bridges
13	A:ARG 57[NH2]	2.99	B:ASP 36[OD2]	Salt bridges
14	A:ARG 9[NH2]	3.21	B:ASP 36[OD2]	Salt bridges
15	A:LYS 432[NZ]	3.95	B:ASP 36[OD2]	Salt bridges
16	A:GLU 239[OE1]	3.43	B:ARG 28[NE]	Salt bridges
17	A:GLU 239[OE1]	3.66	B:ARG 28[NH1]	Salt bridges
18	A:GLU 239[OE1]	3.07	B:ARG 28[NH2]	Salt bridges
19	A:GLU 398[OE1]	3.77	B:ARG 30[NE]	Salt bridges
20	A:GLU 398[OE1]	2.26	B:ARG 30[NH1]	Salt bridges
21	A:GLU 398[OE1]	3.08	B:ARG 30[NH2]	Salt bridges
