# Supplementary material for: MAZ-mediated up-regulation of BCKDK reprograms glucose metabolism and promotes growth by regulating glucose-6-phosphate dehydrogenase stability in triple-negative breast cancer
Source: Cell Death Dis. 2024 Jul 18;15(7):516. doi: 10.1038/s41419-024-06835-y (PMC11258276; doi:10.1038/s41419-024-06835-y)

Figure 2a

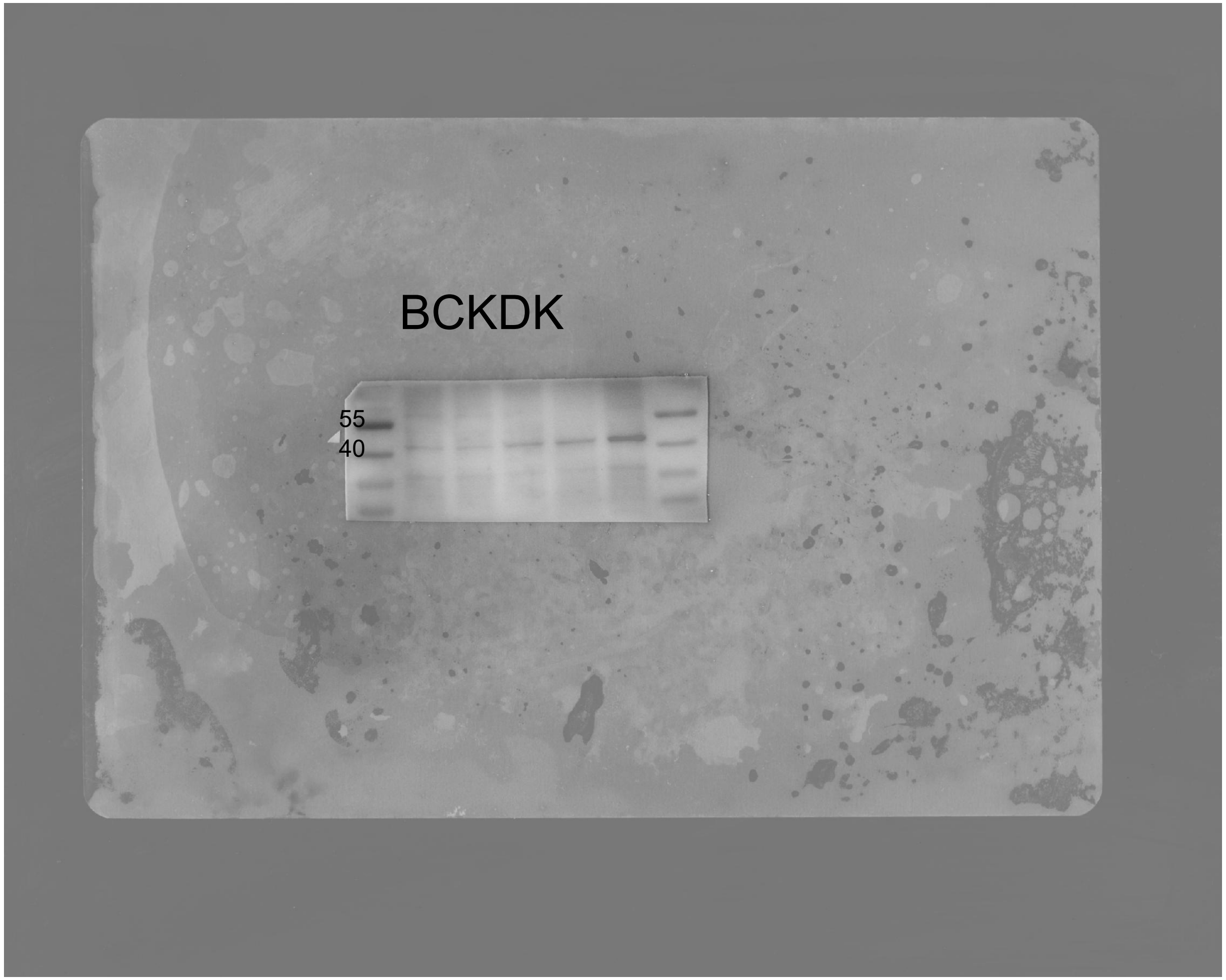

Figure 2a

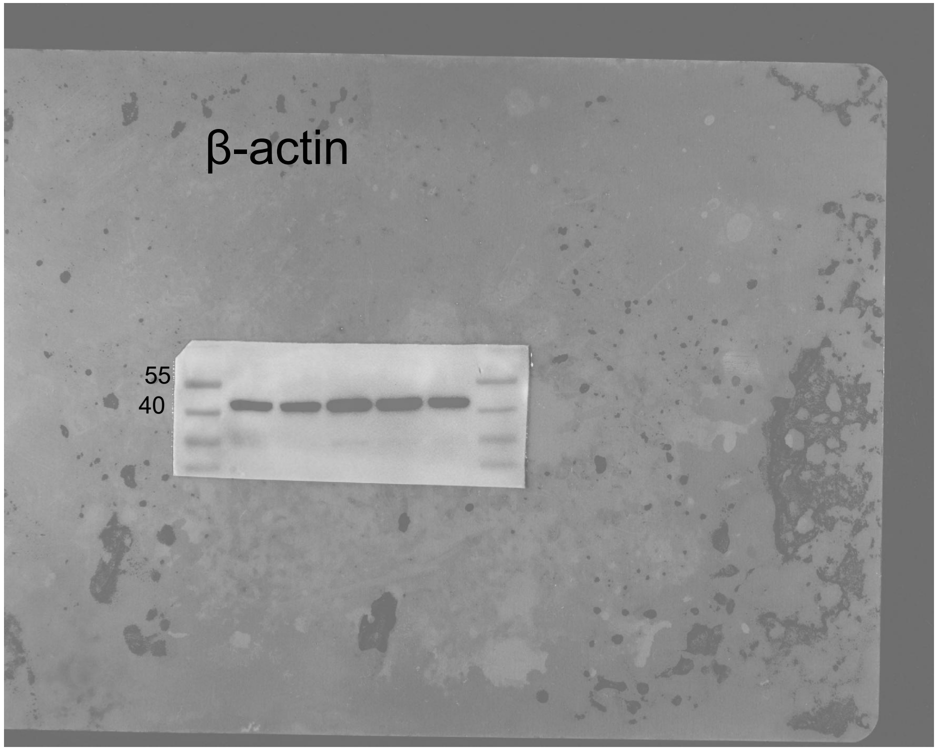

Figure 2b

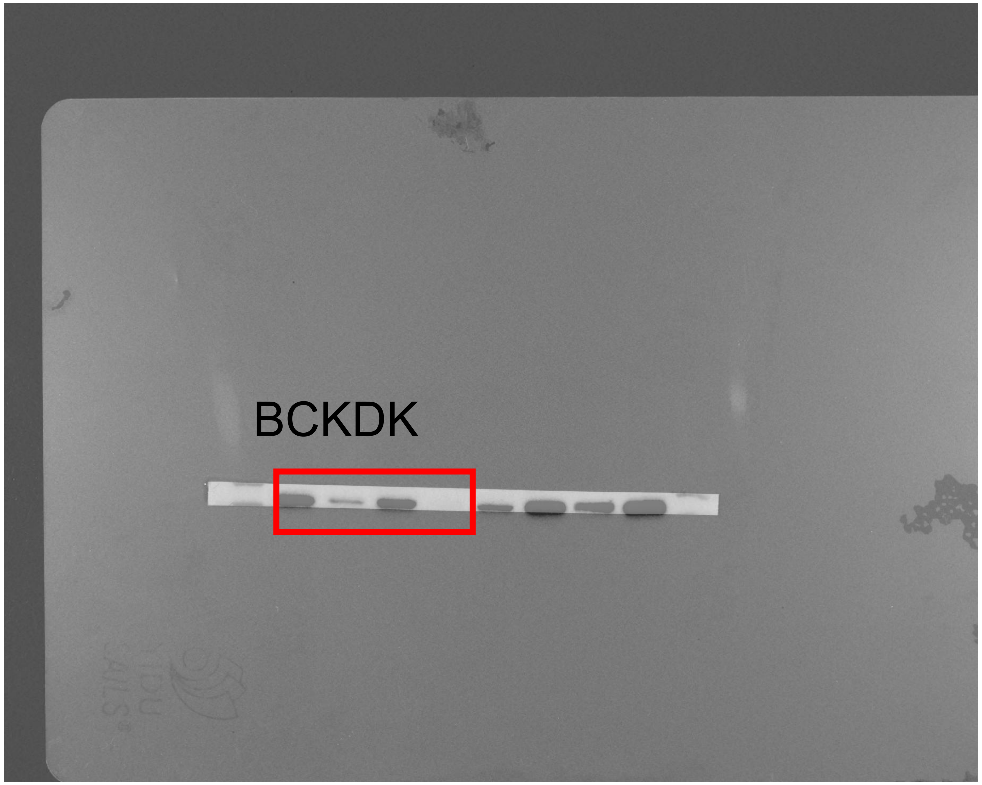

Figure 2b

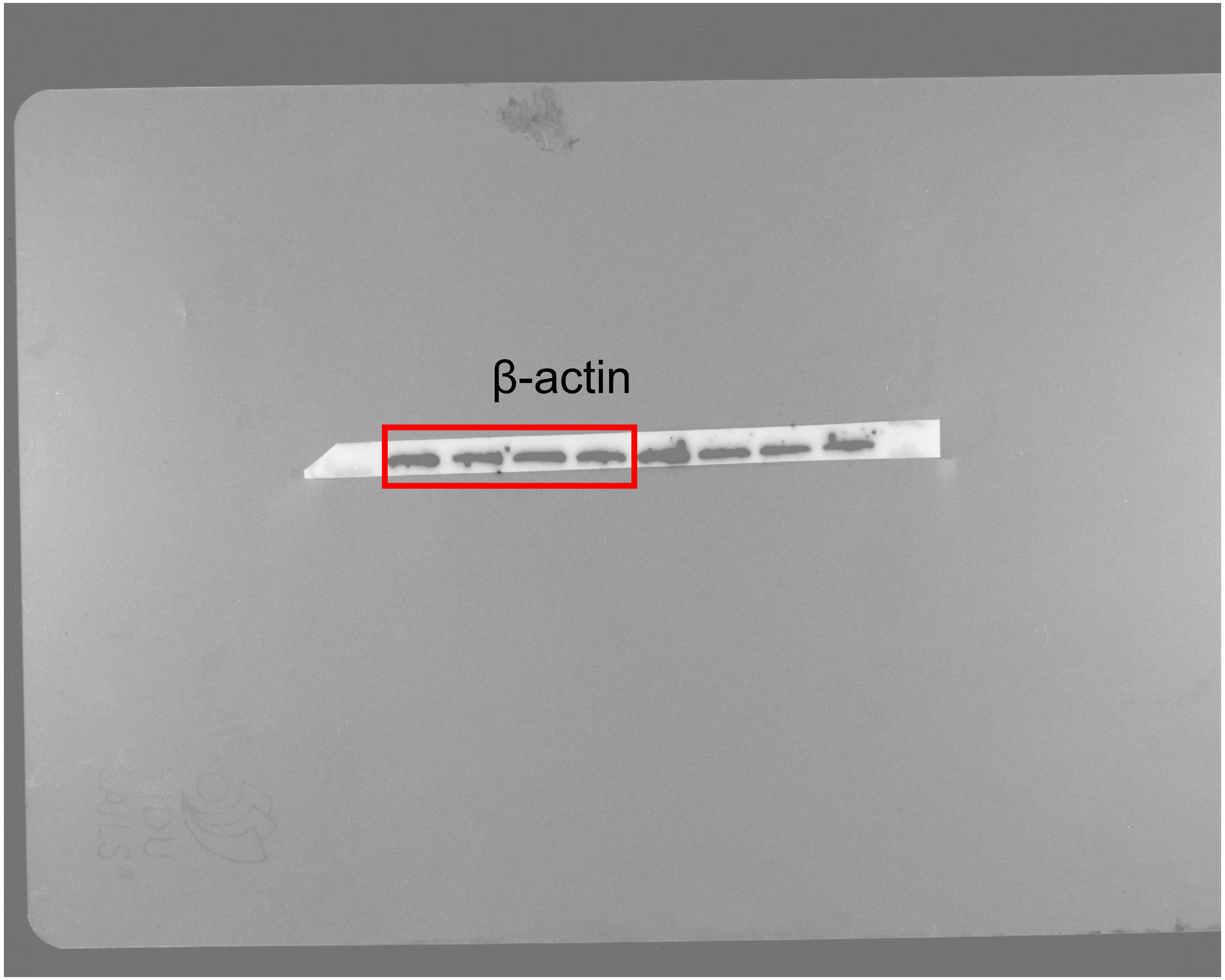

Figure 4b

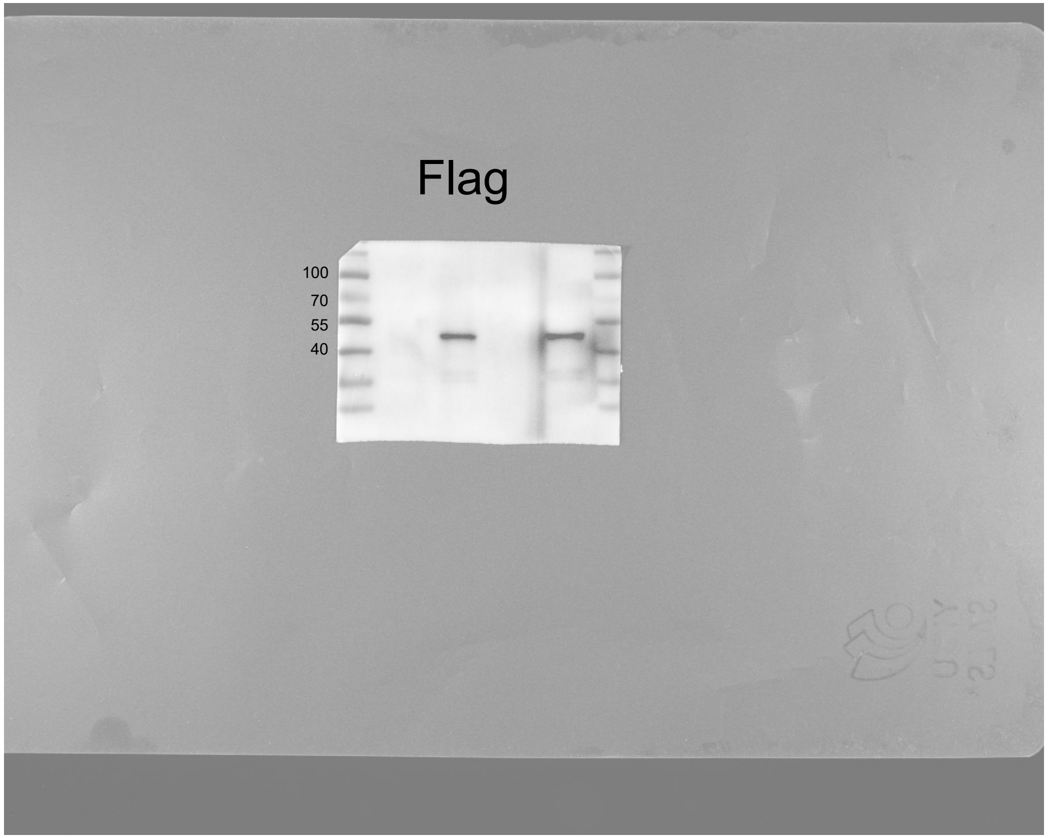

Figure 4b

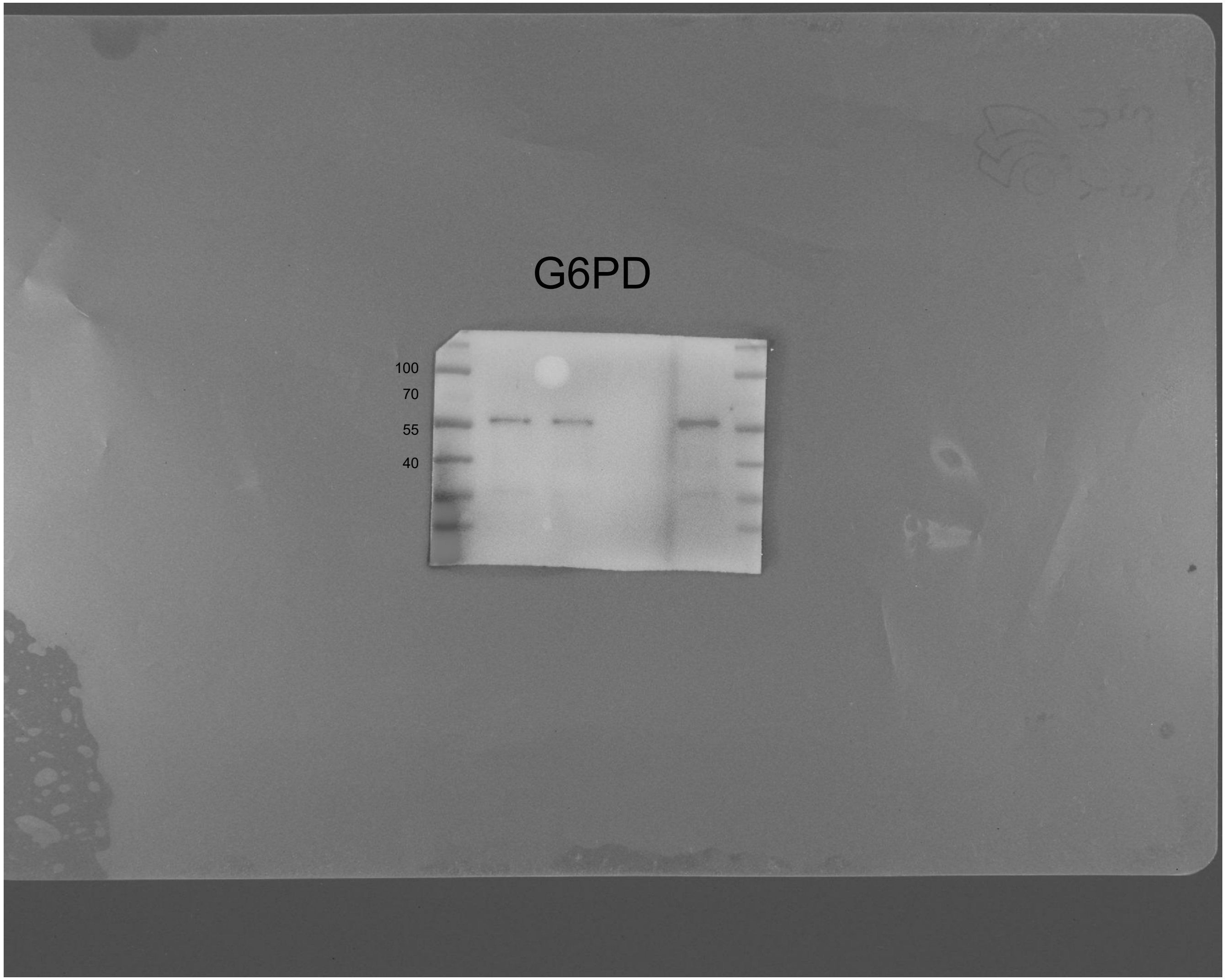

Figure 4d upper

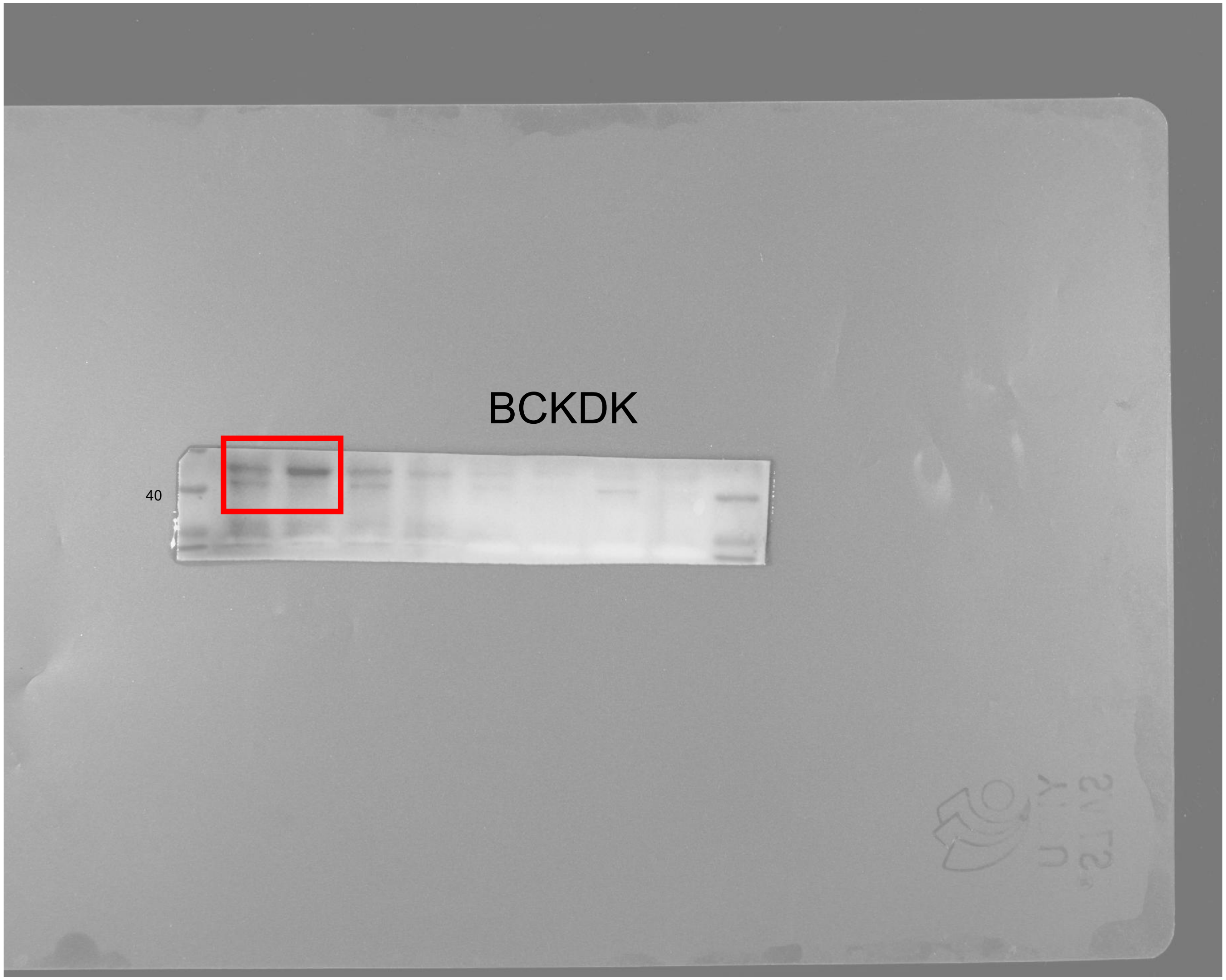

Figure 4d upper

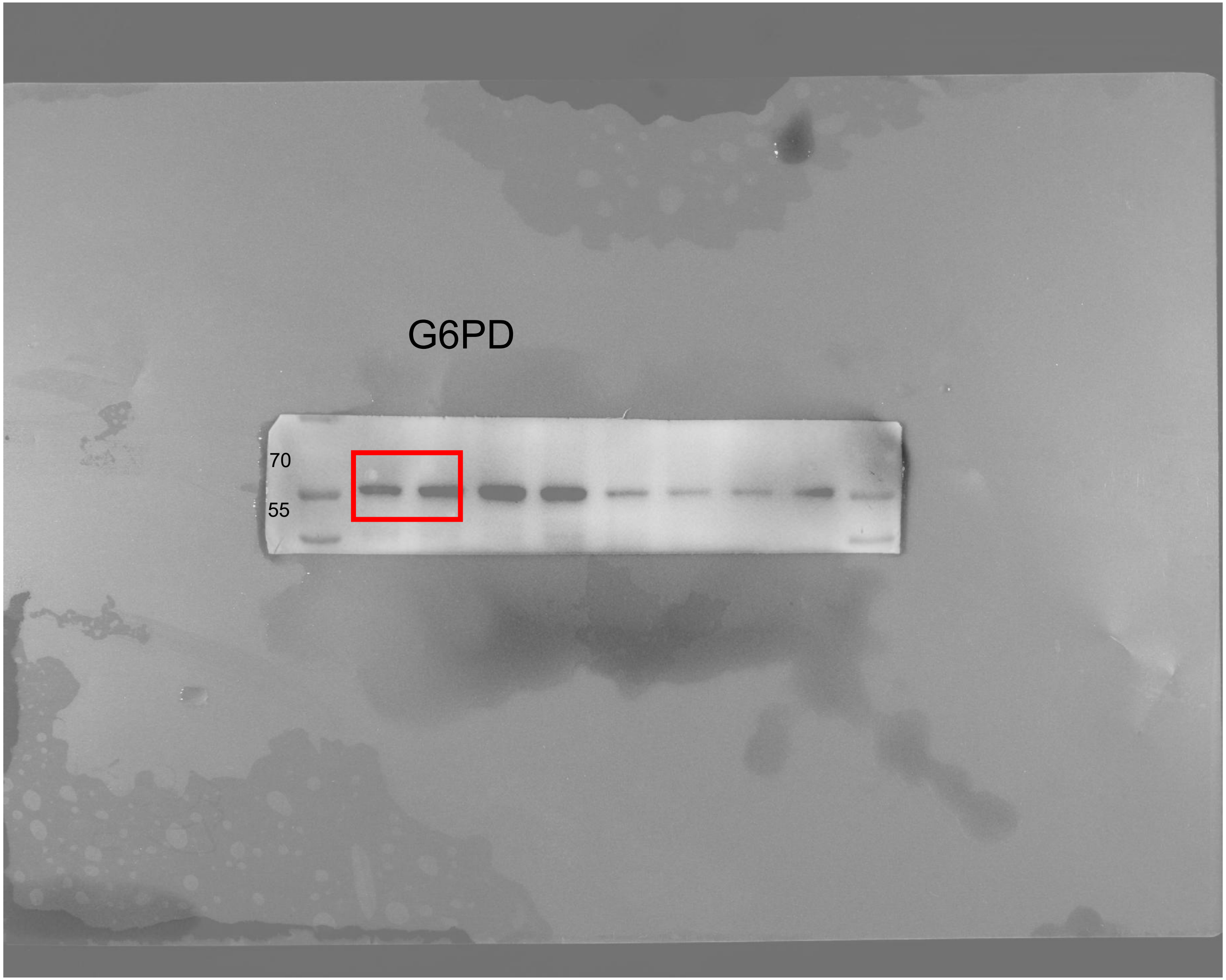

Figure 4d upper

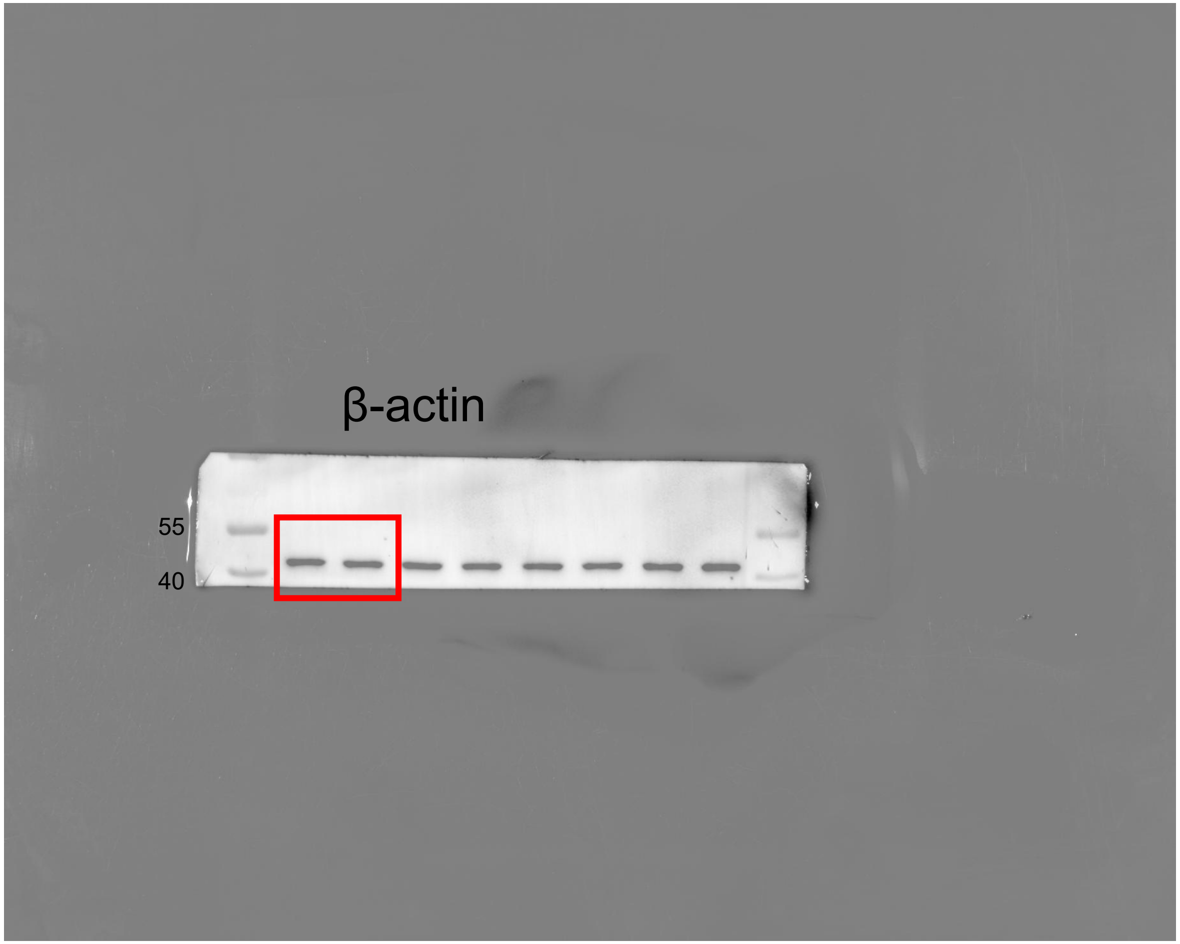

Figure 4d lower

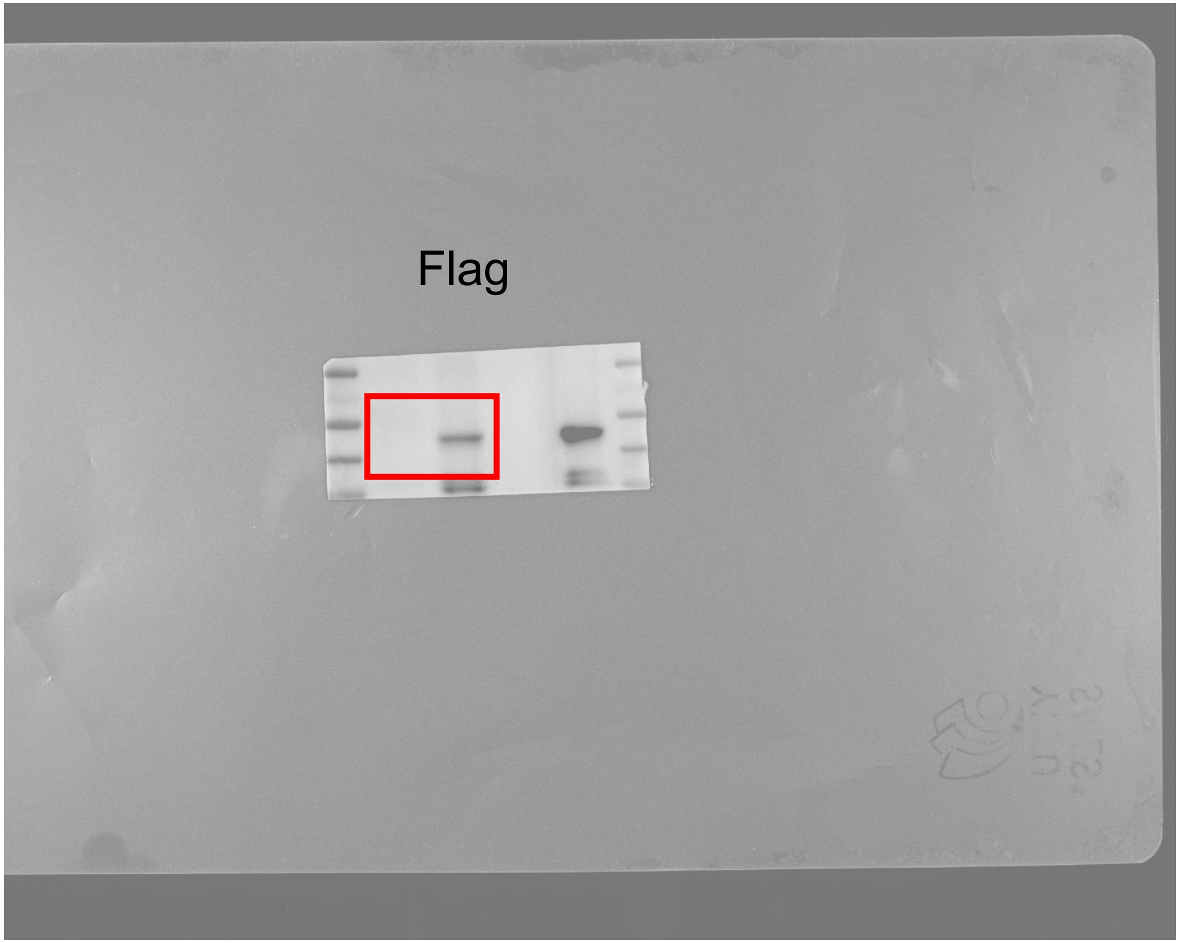

Figure 4d lower

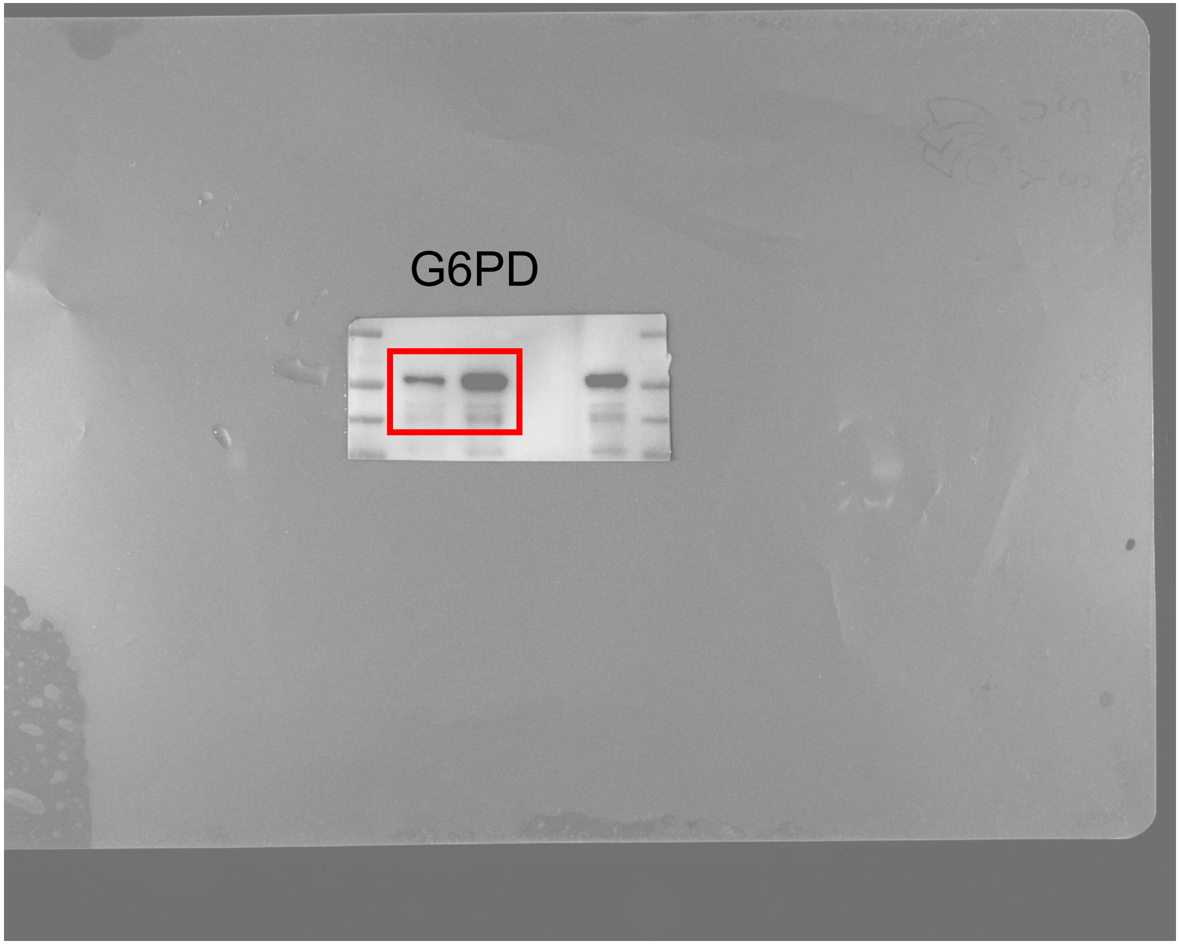

Figure 4d lower

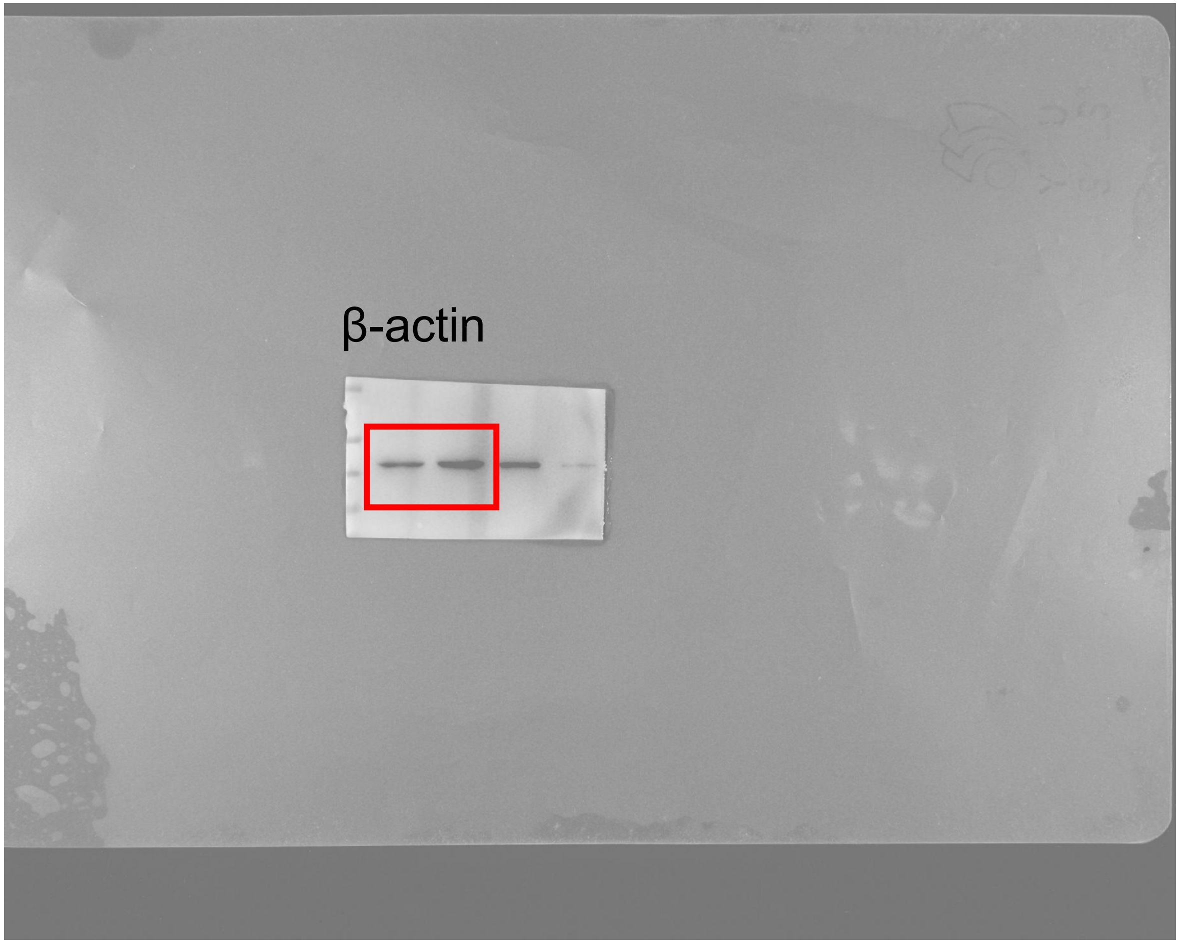

Figure 4e left

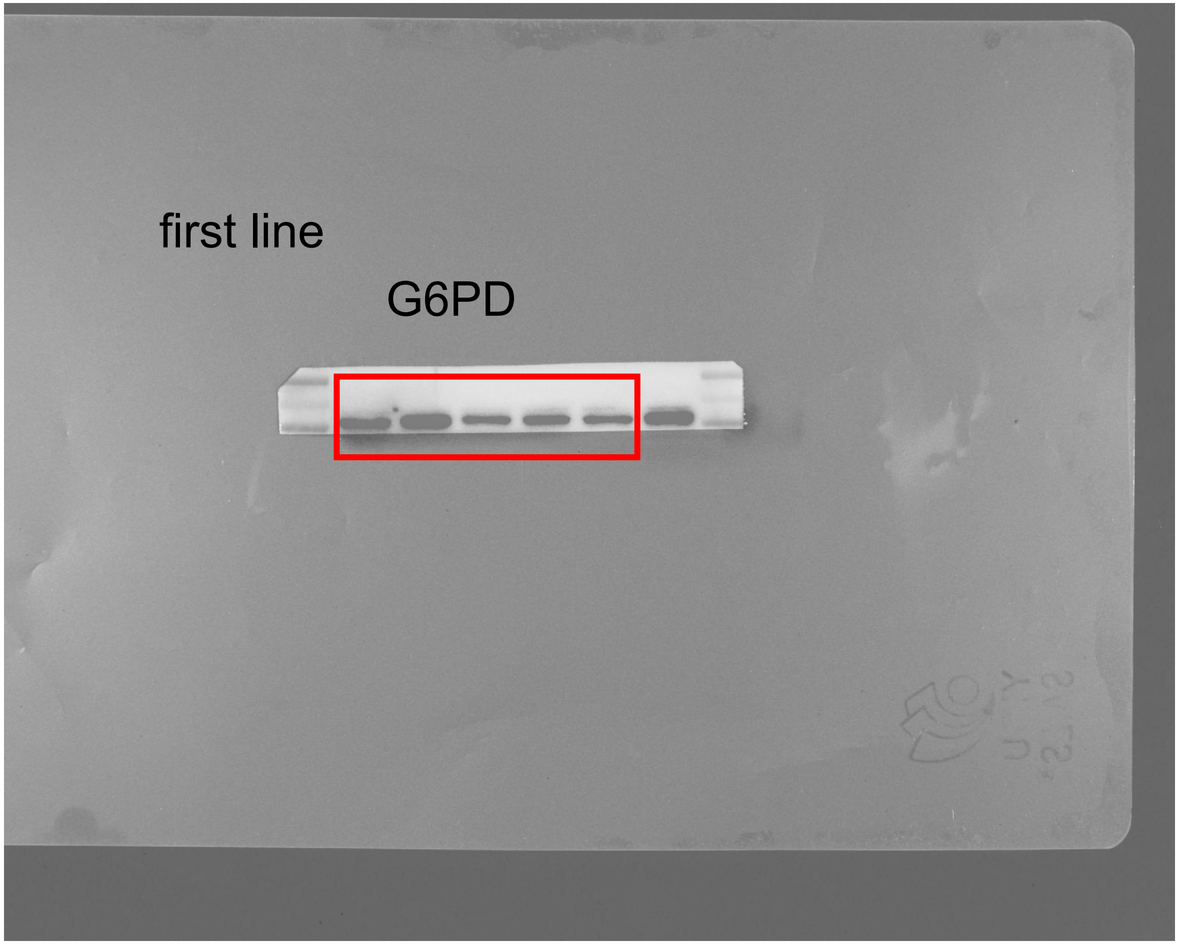

Figure 4e left

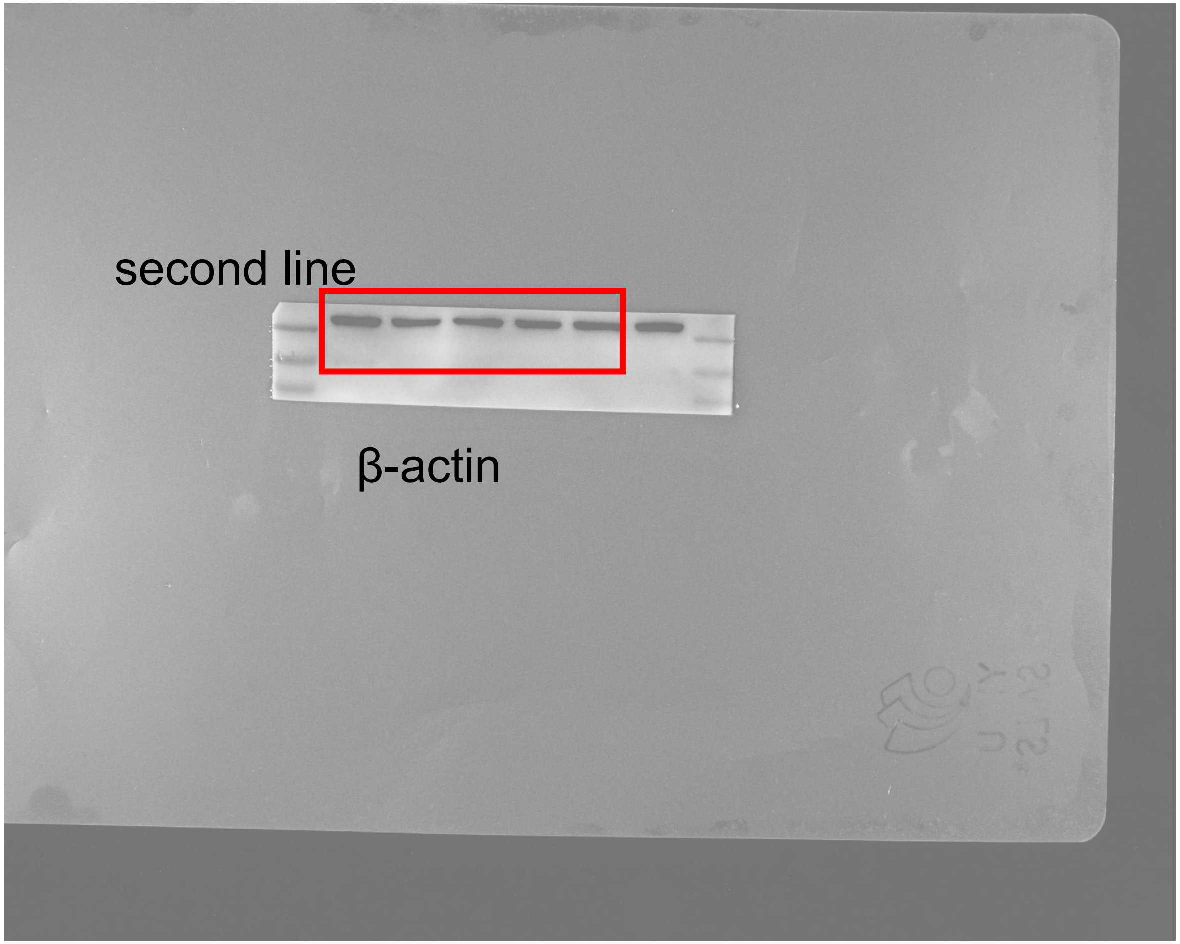

Figure 4e left

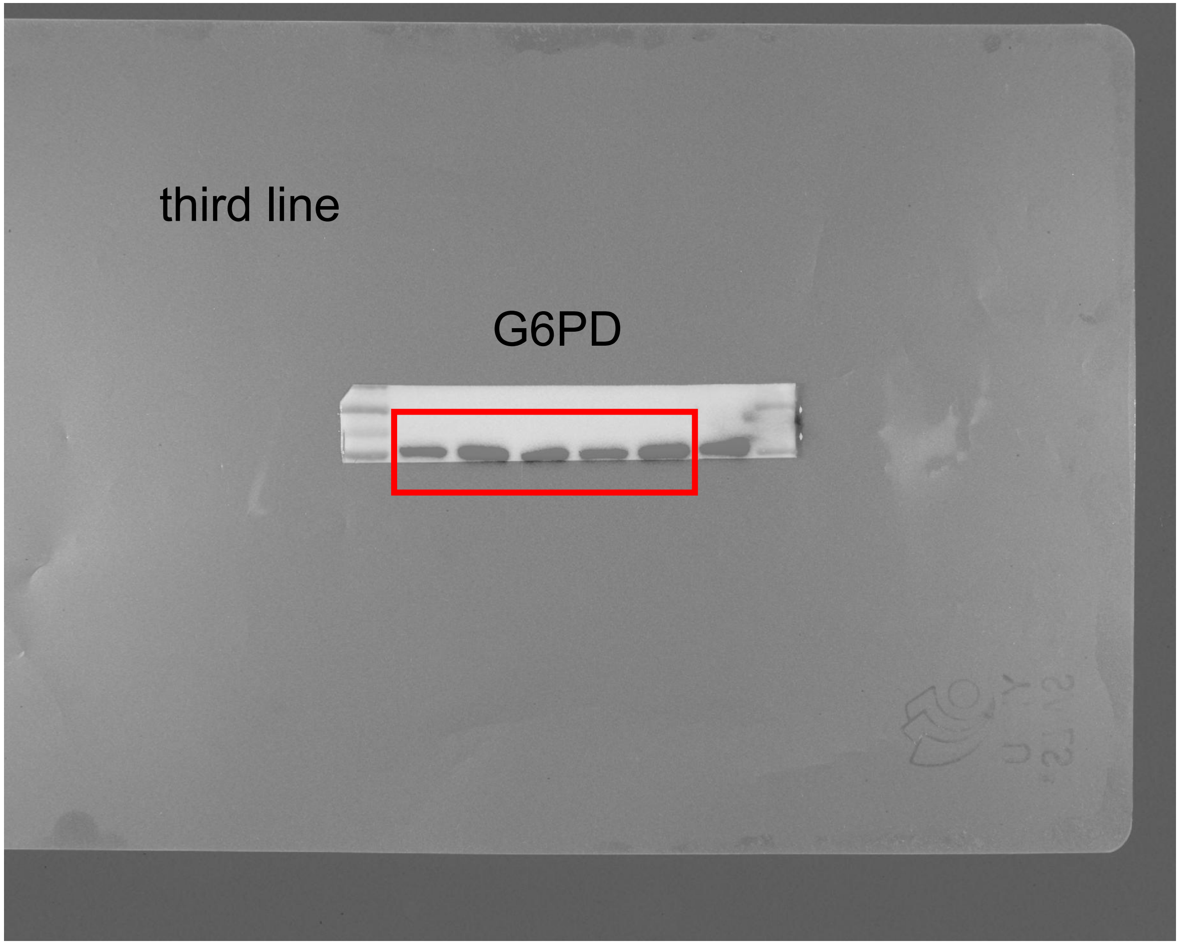

Figure 4e left

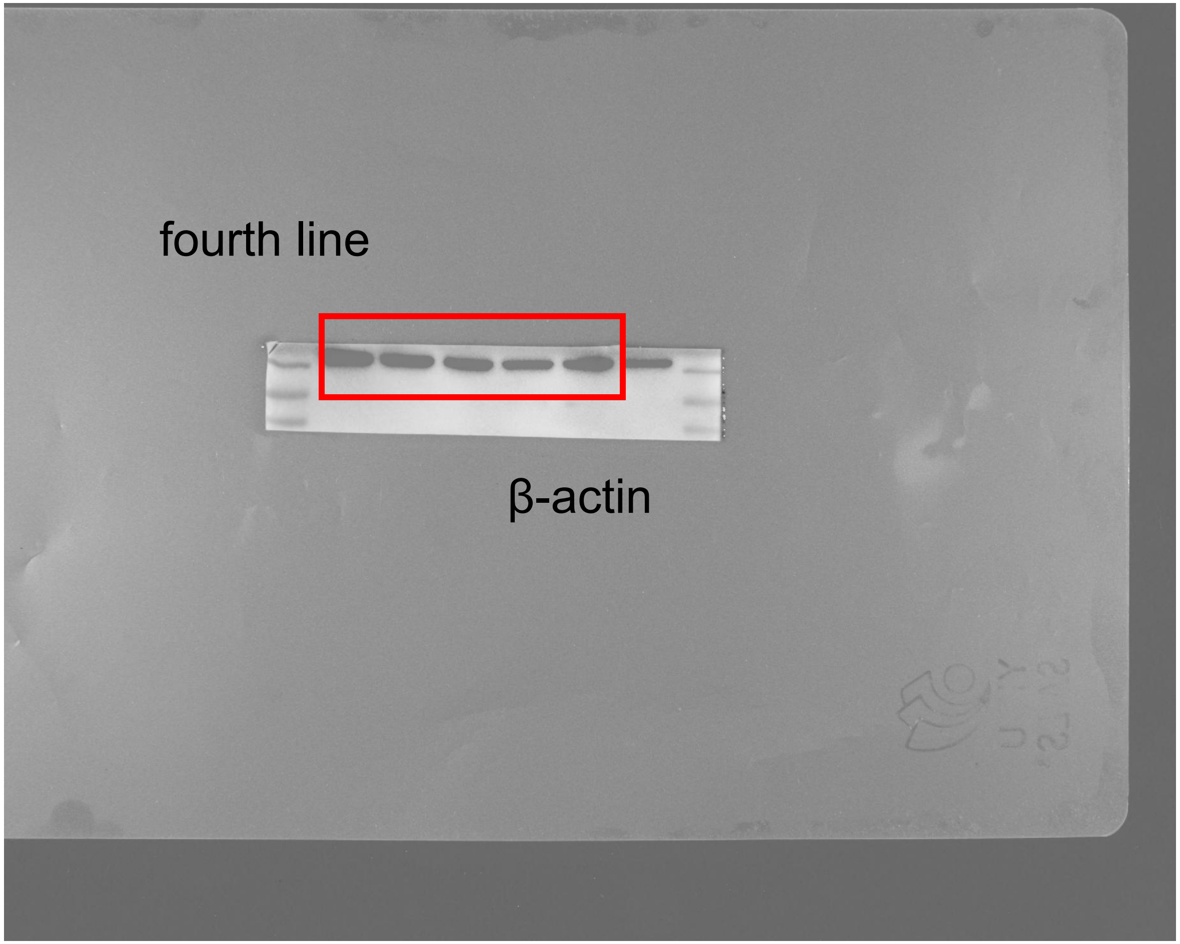

Figure 4e right

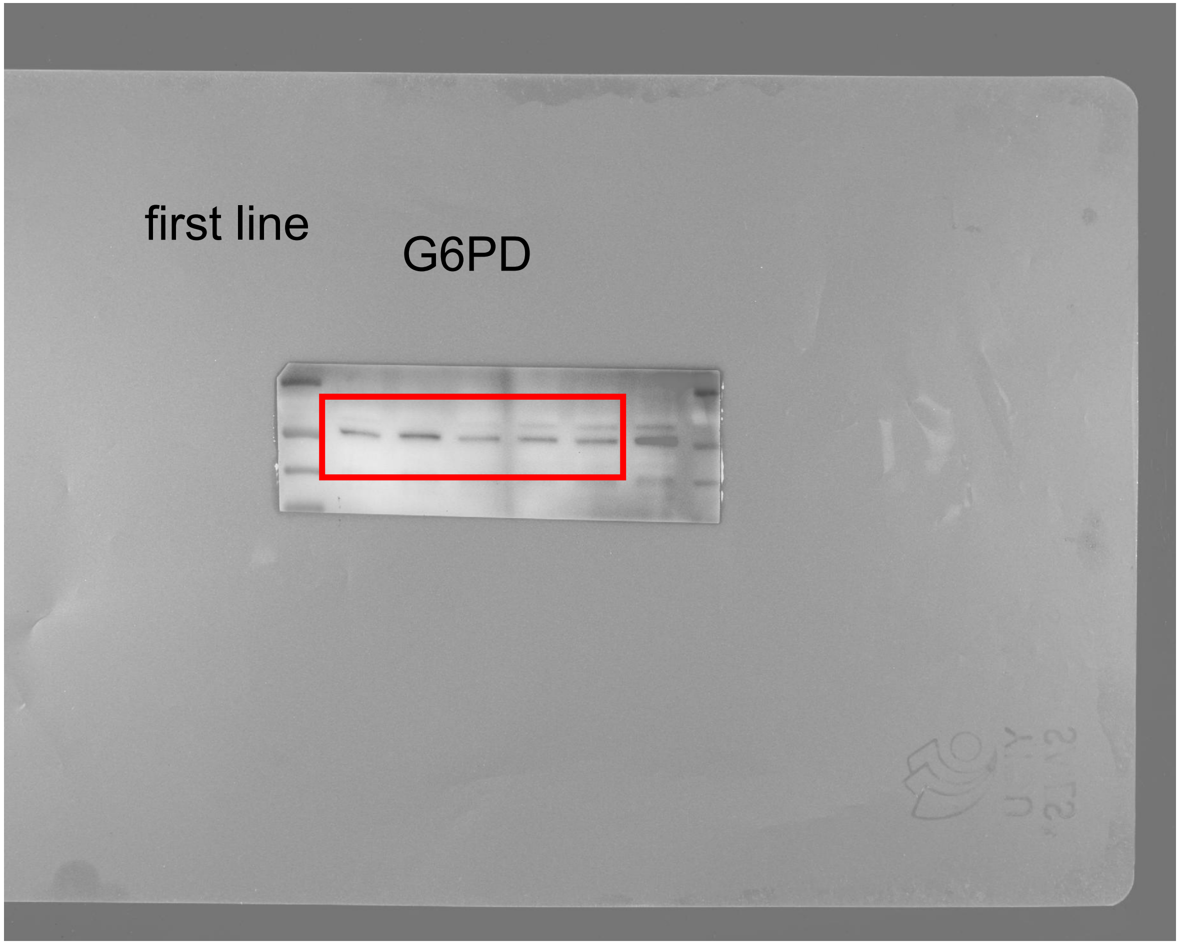

Figure 4e right

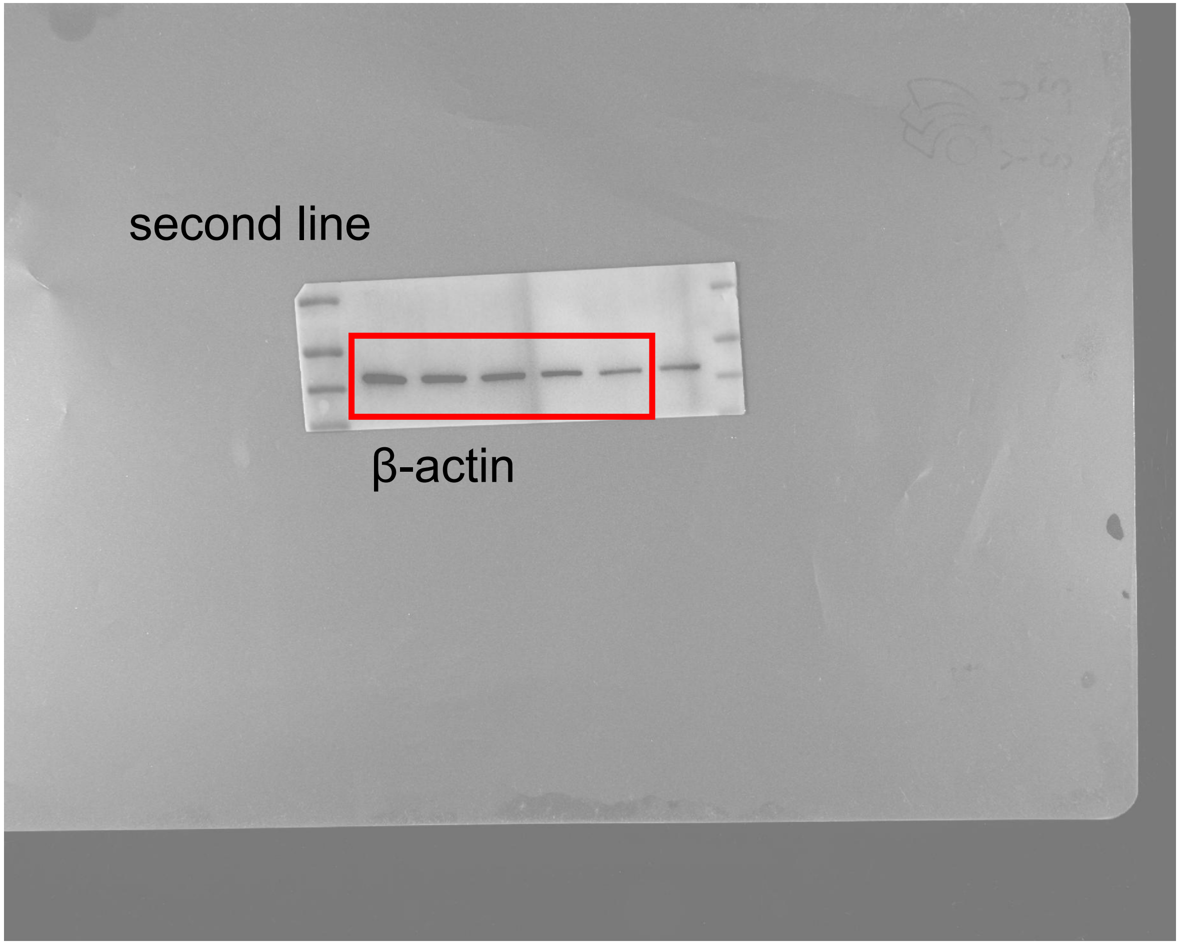

Figure 4e right

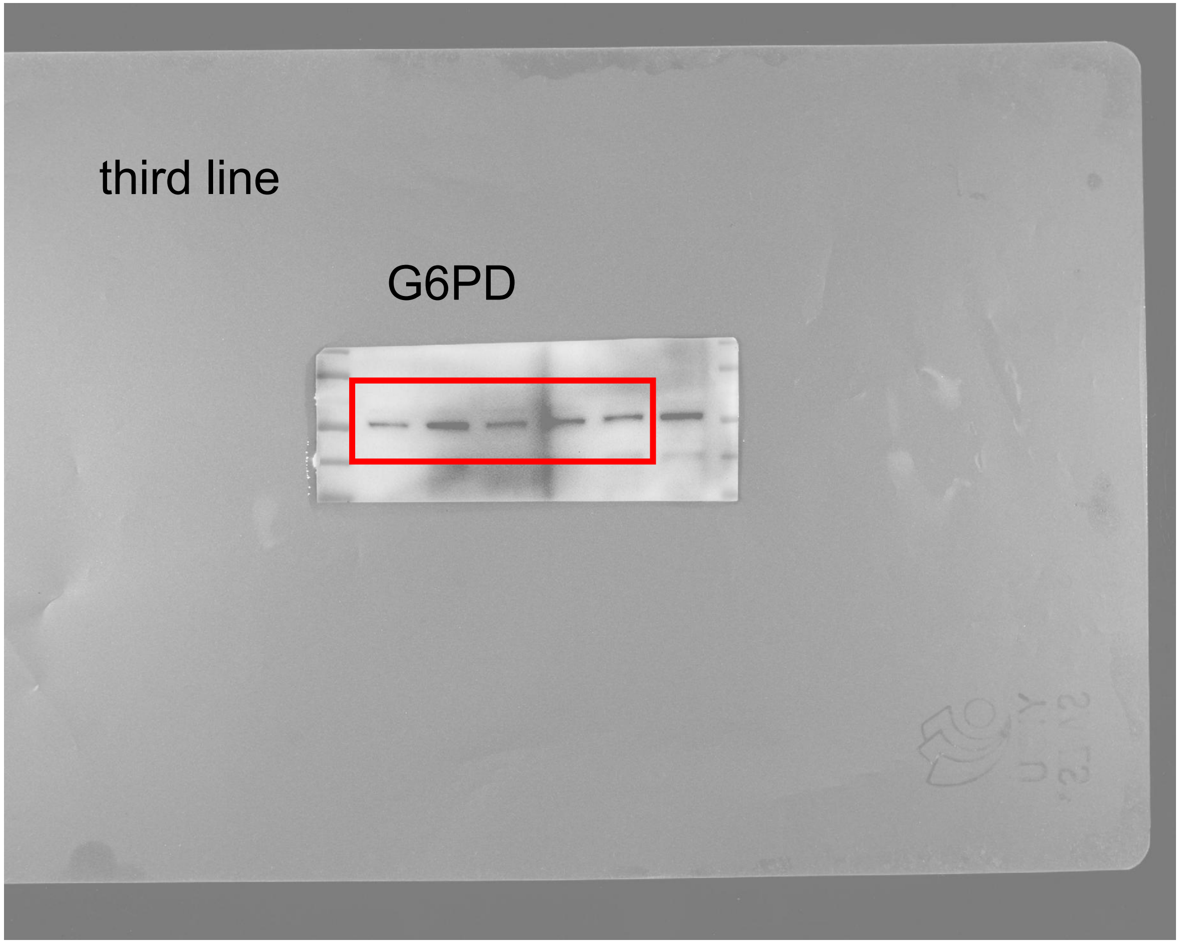

Figure 4e right

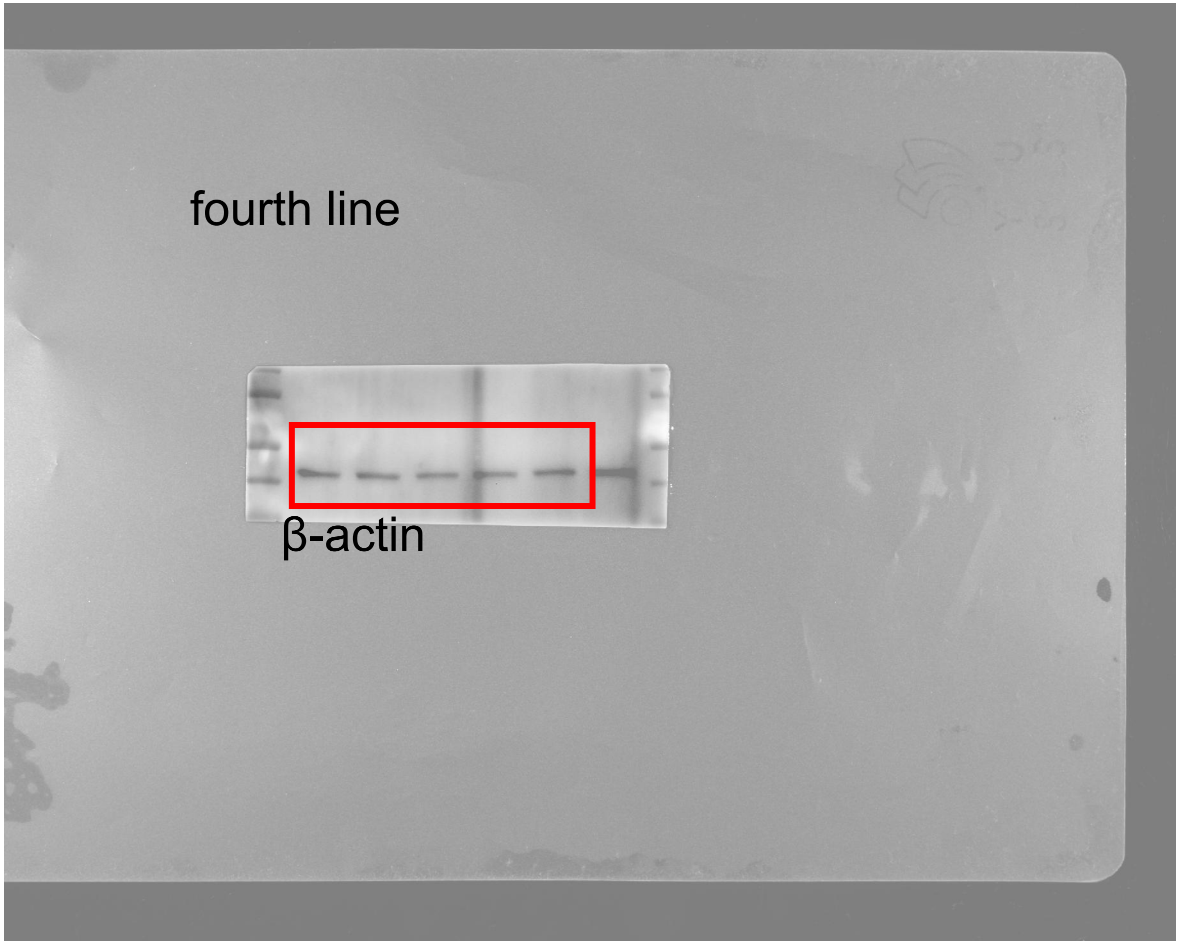

Figure 4f

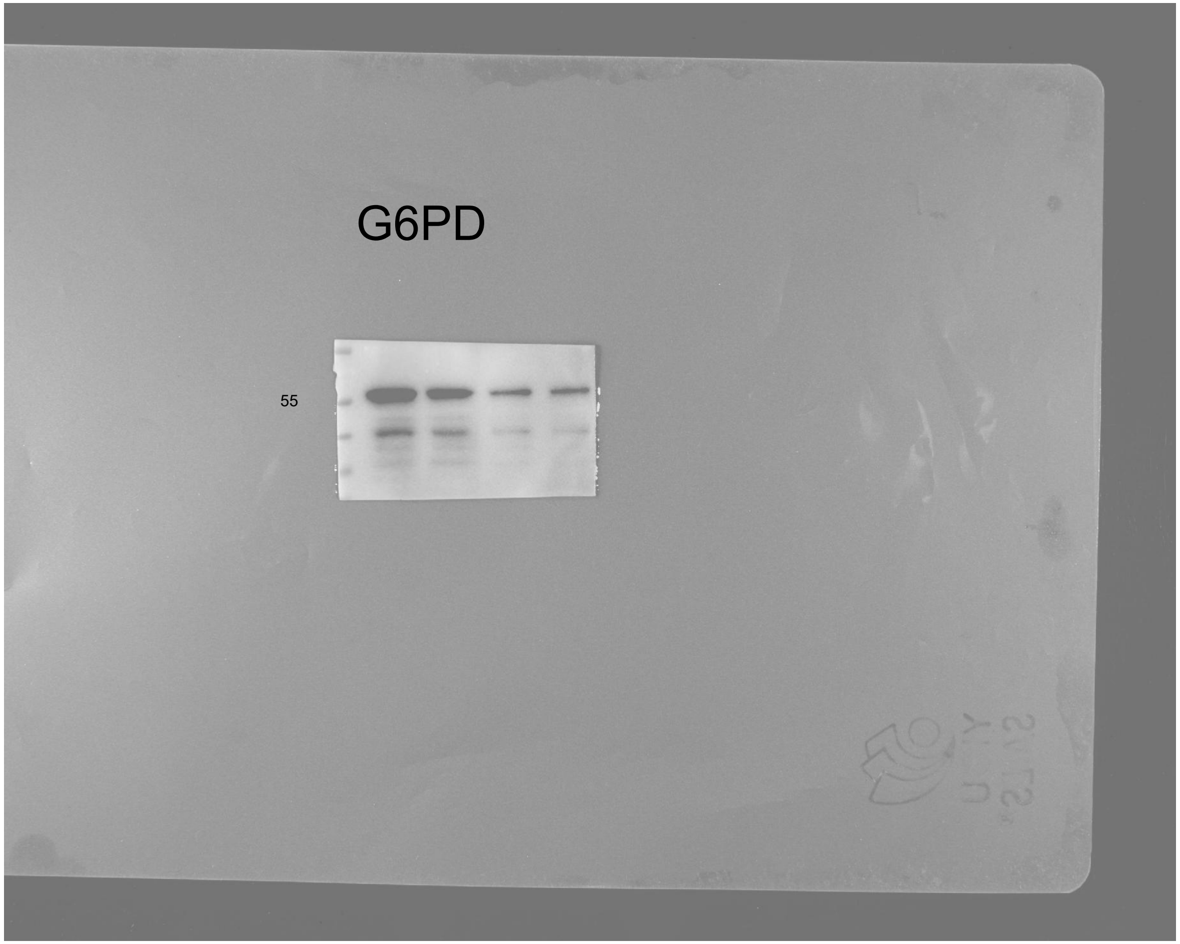

Figure 4f

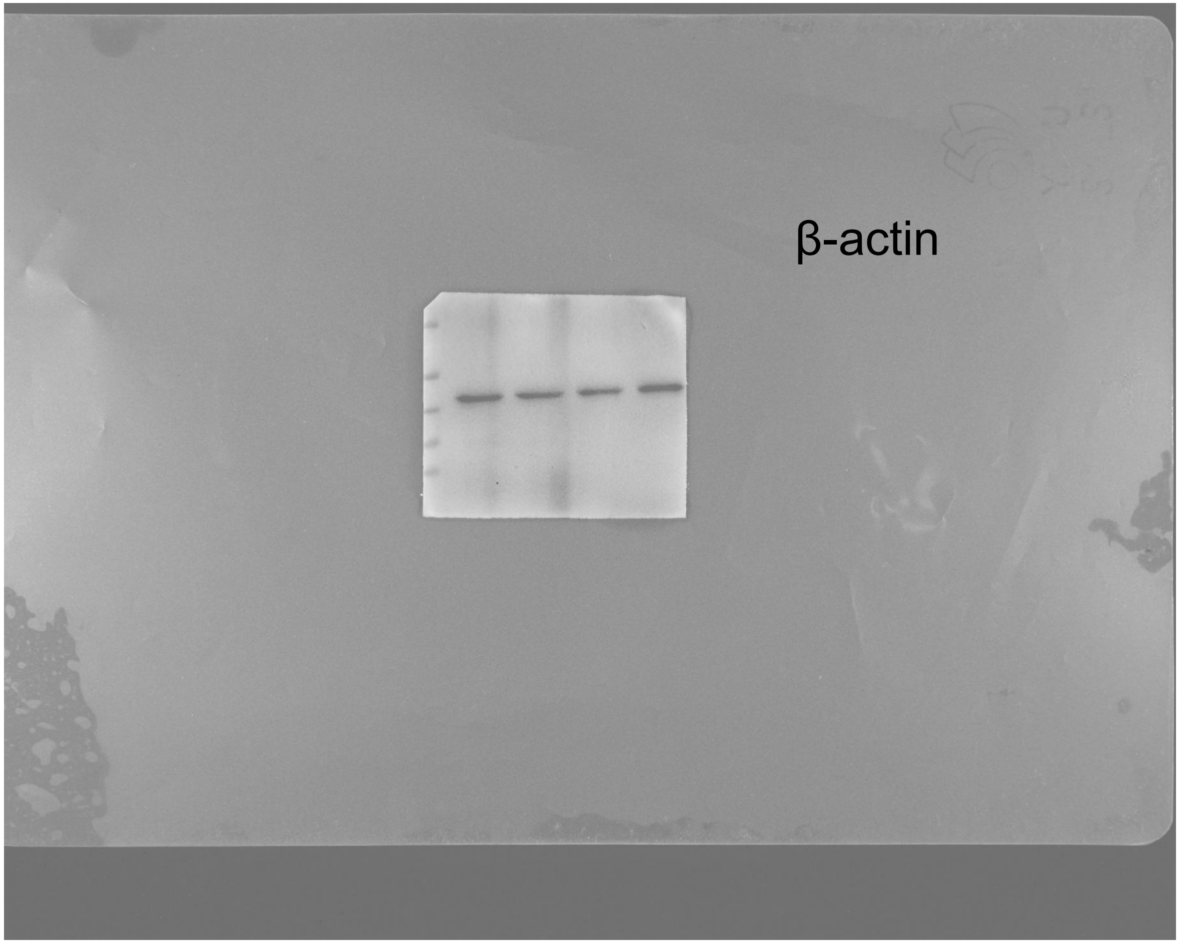

Figure 5e left

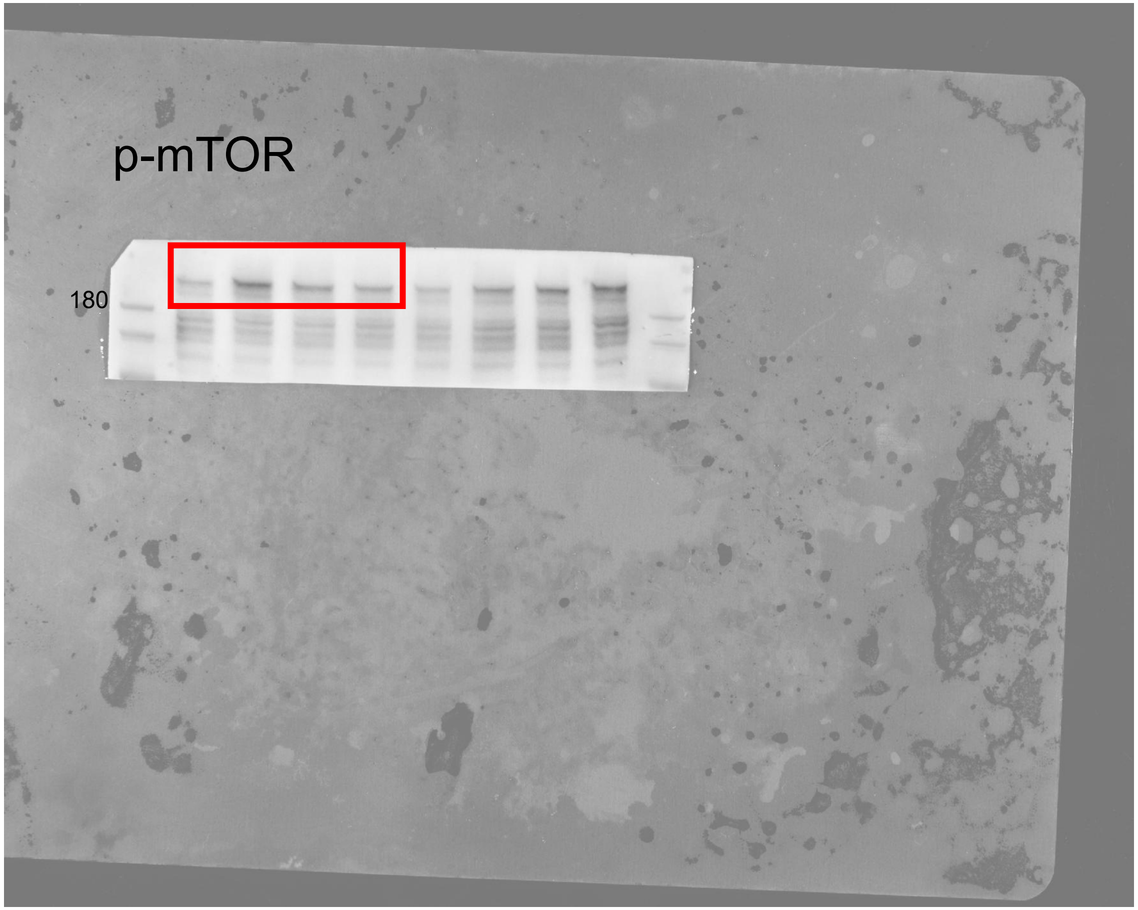

Figure 5e left

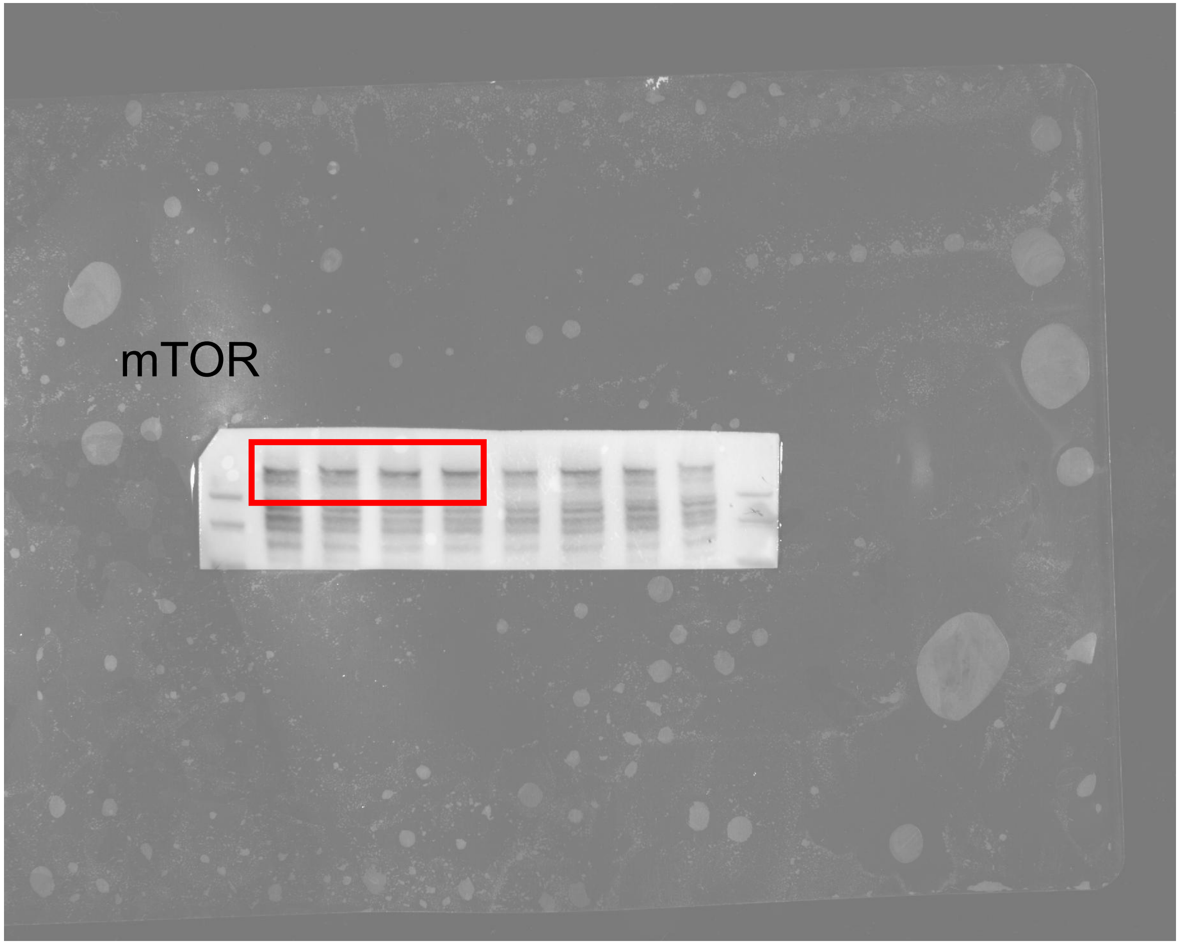

Figure 5e left

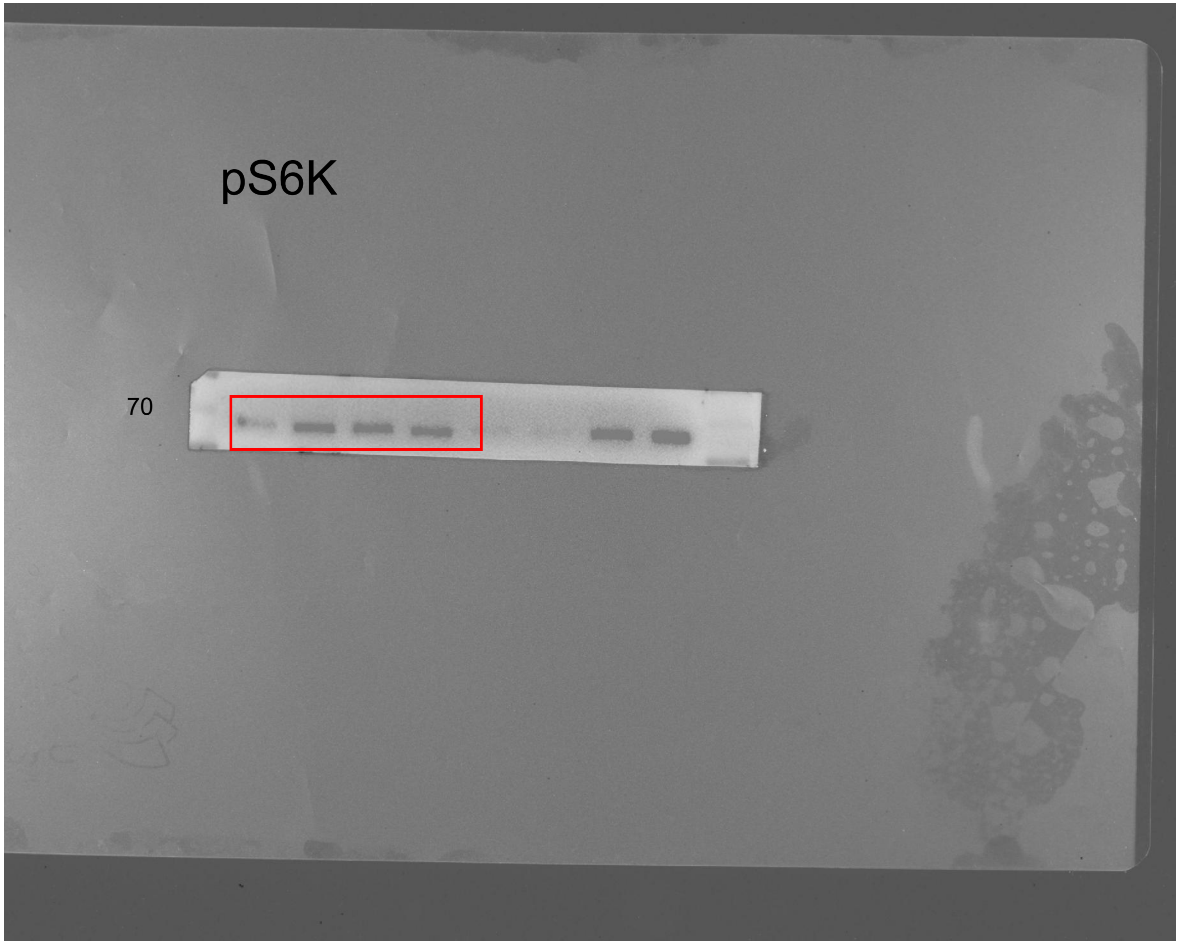

Figure 5e left

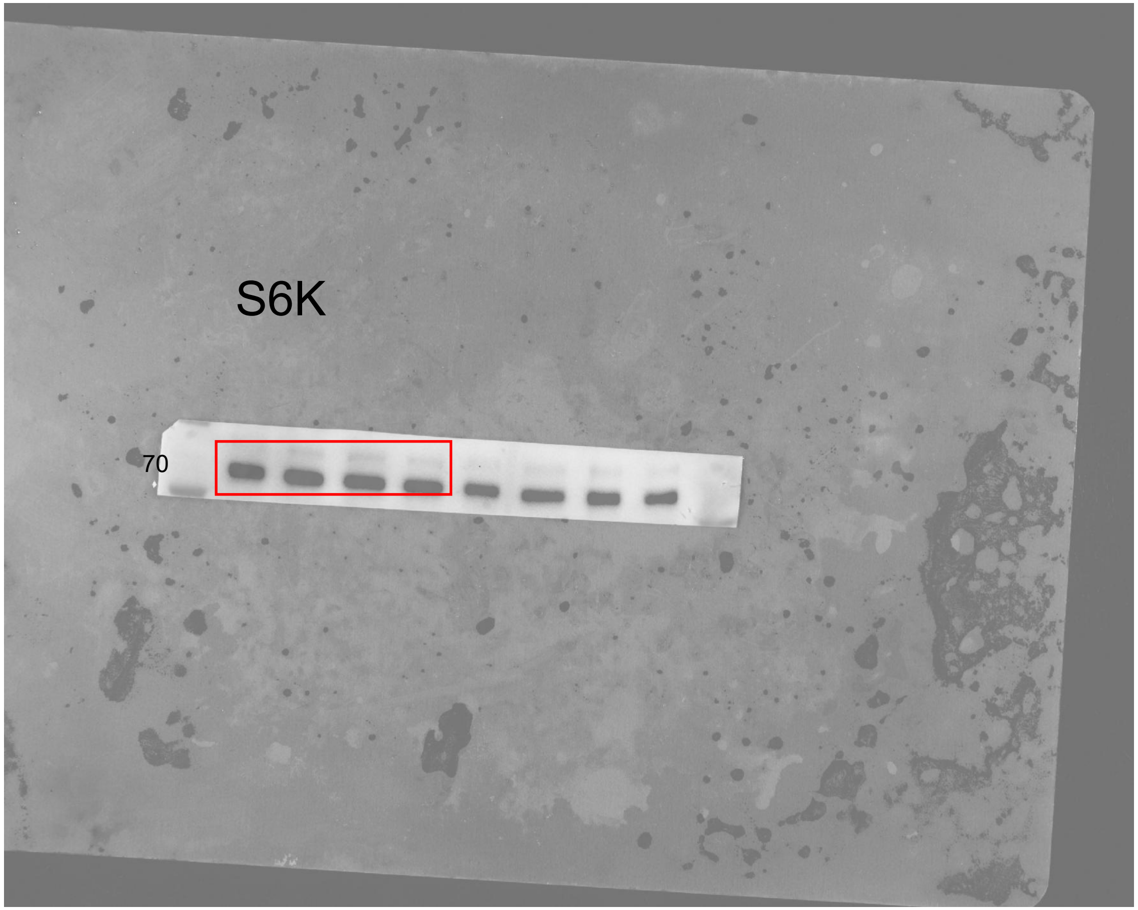

Figure 5e left

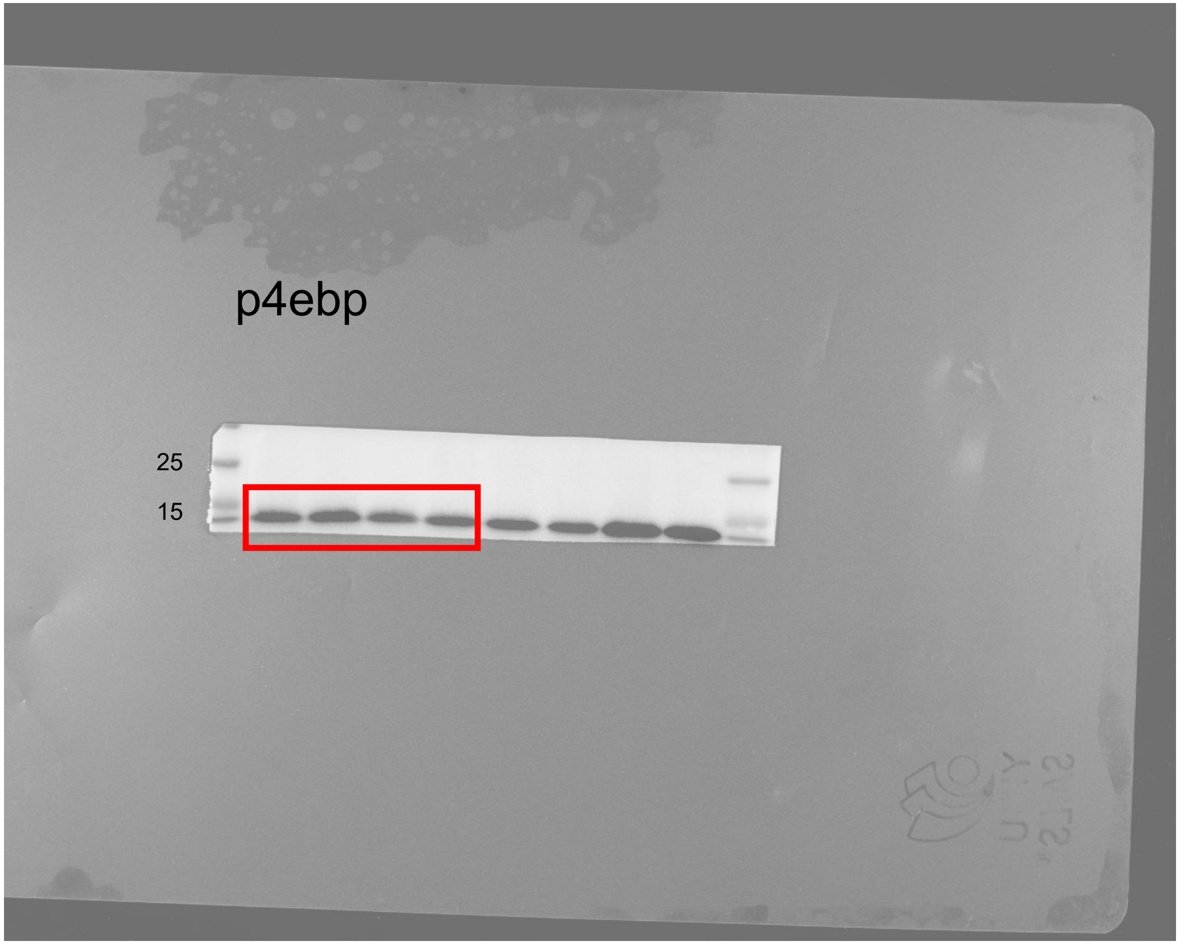

Figure 5e left

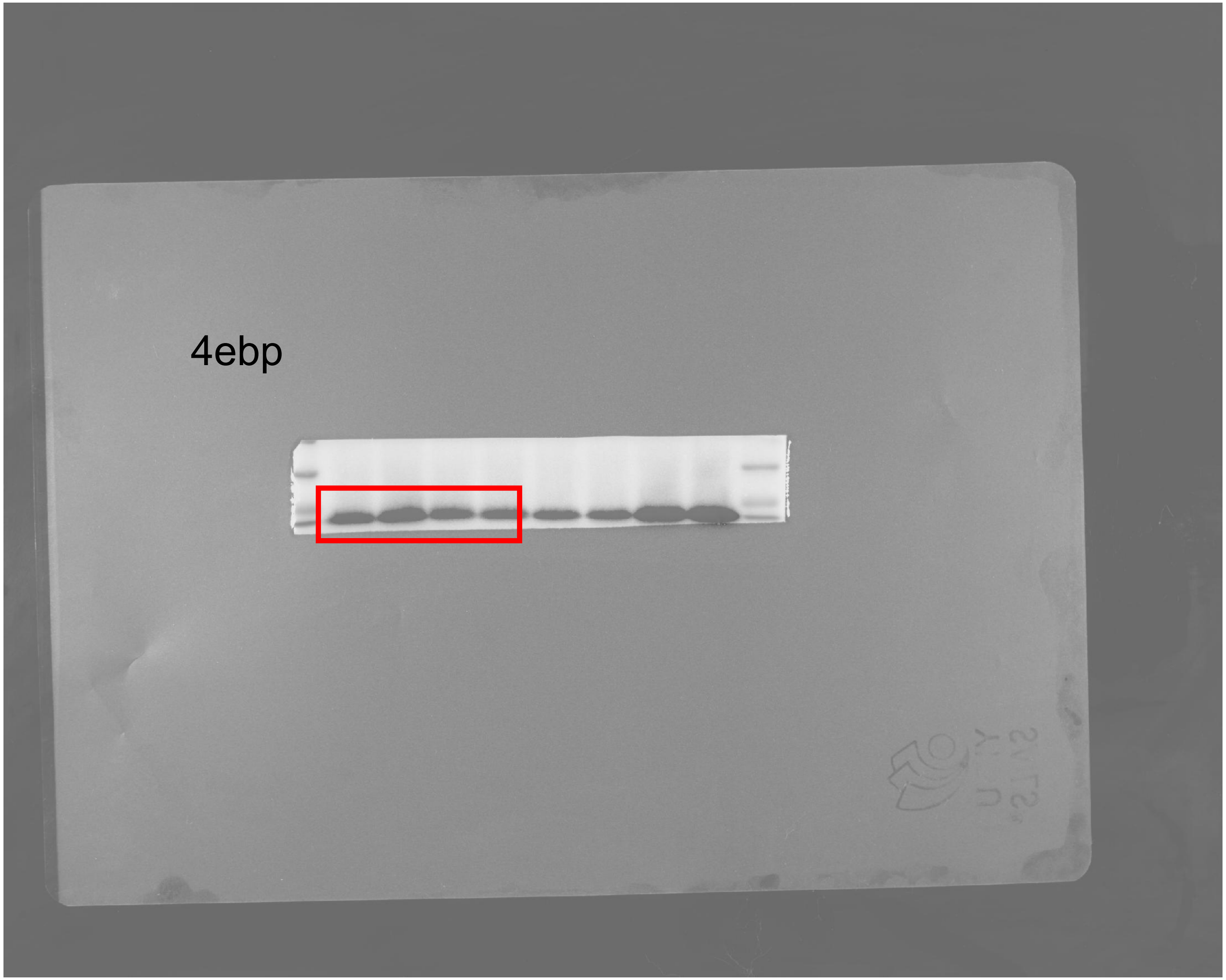

Figure 5e left

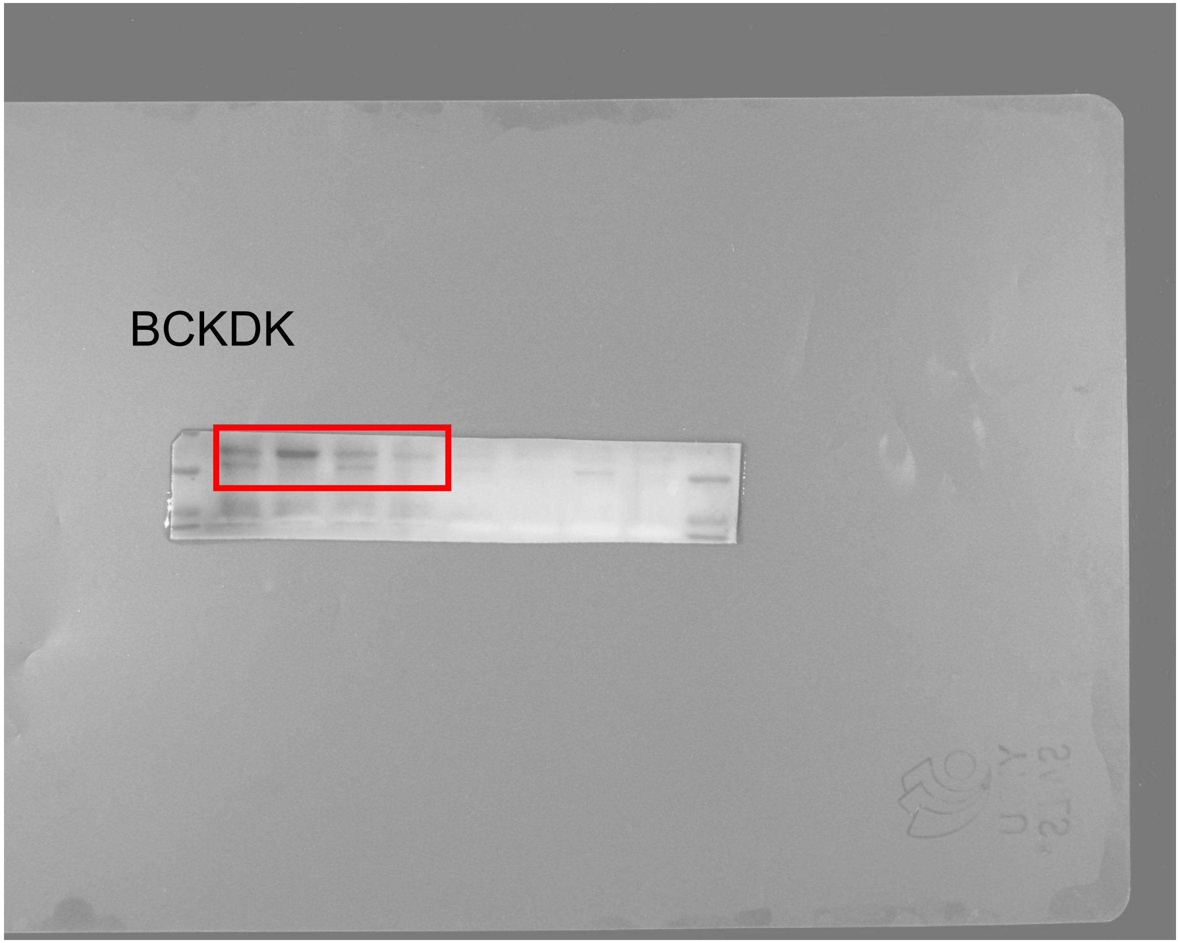

Figure 5e left

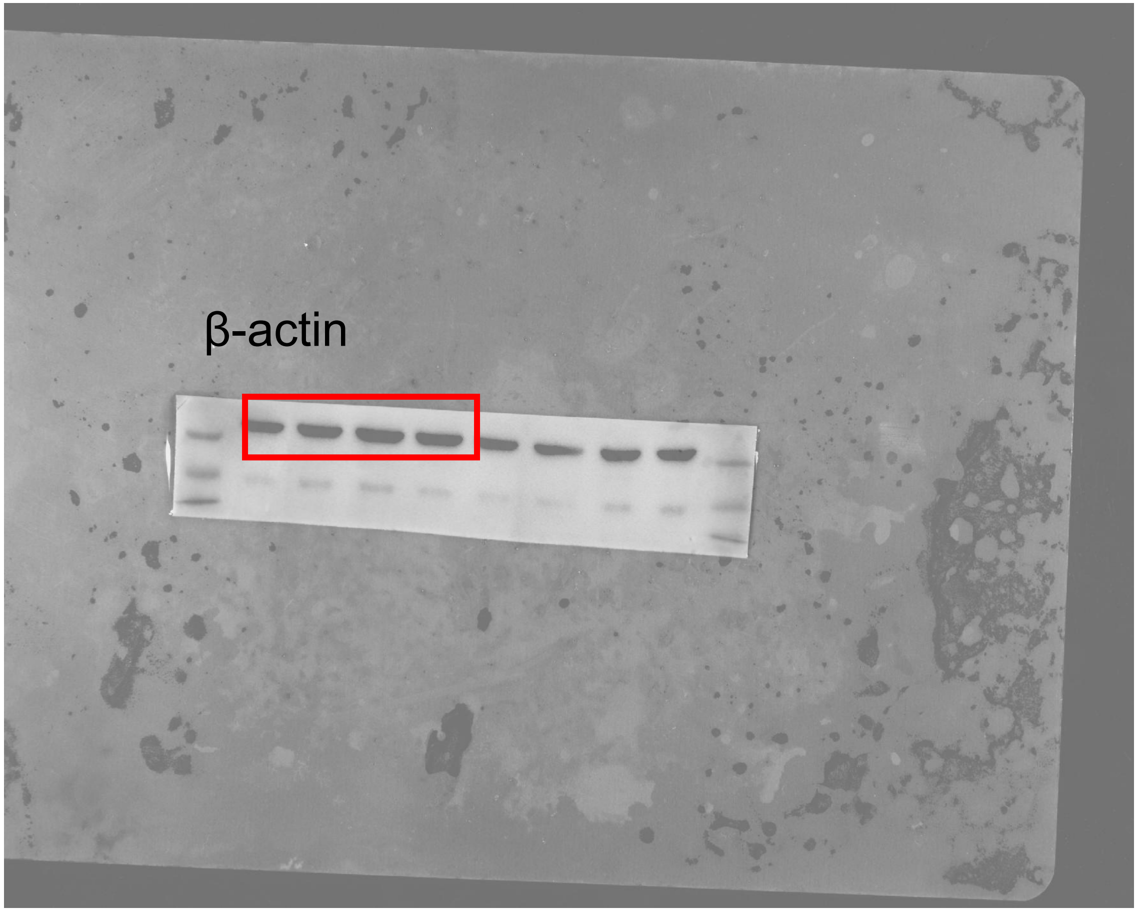

Figure 5e right

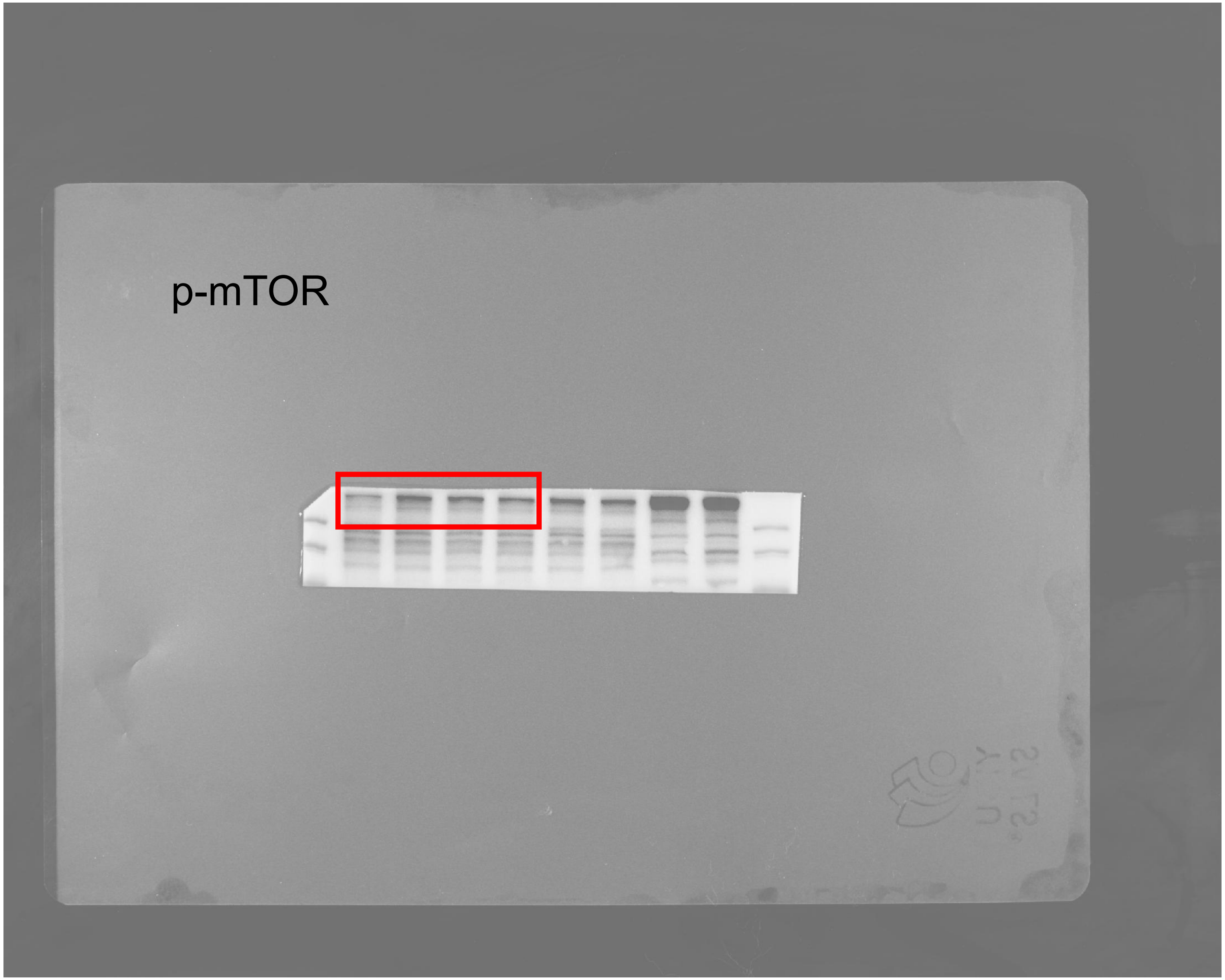

Figure 5e right

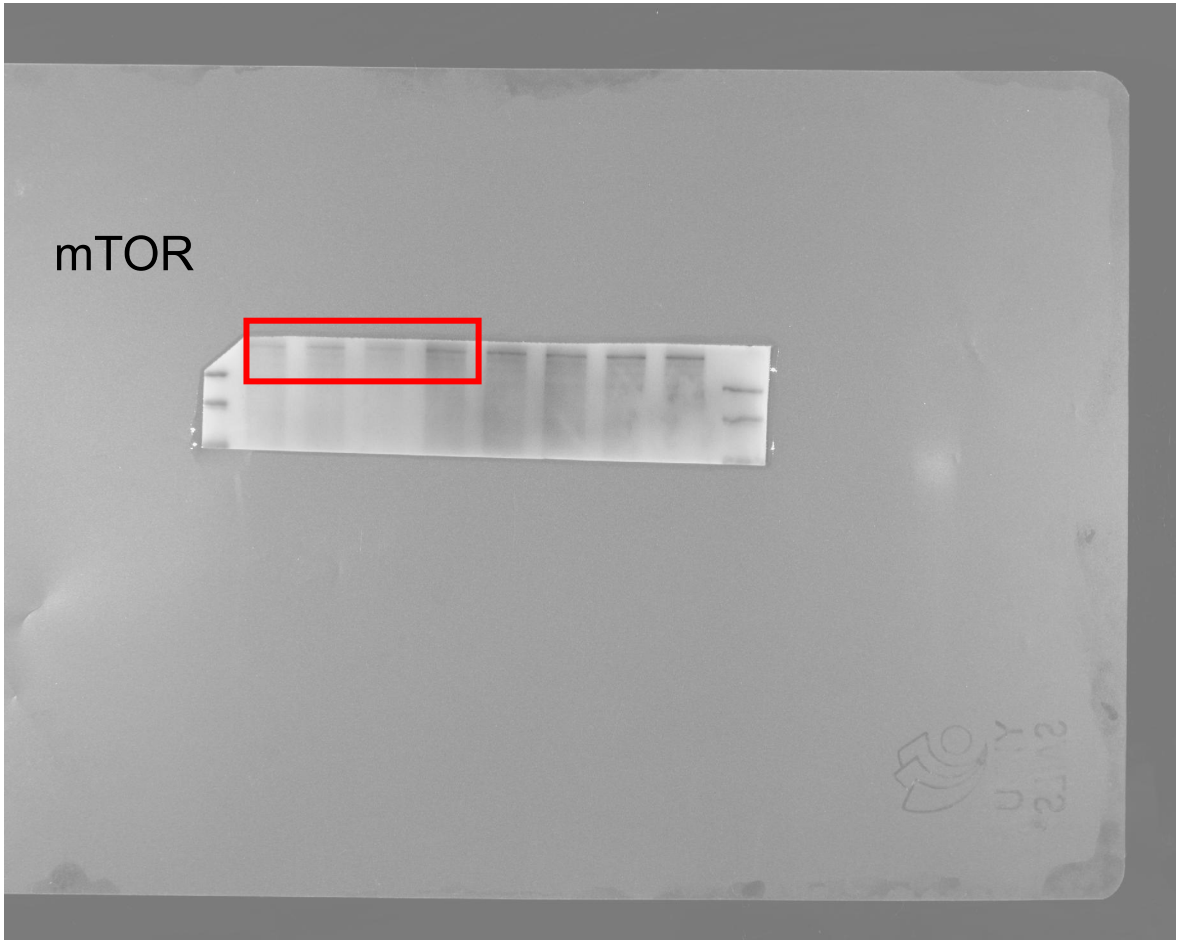

Figure 5e right

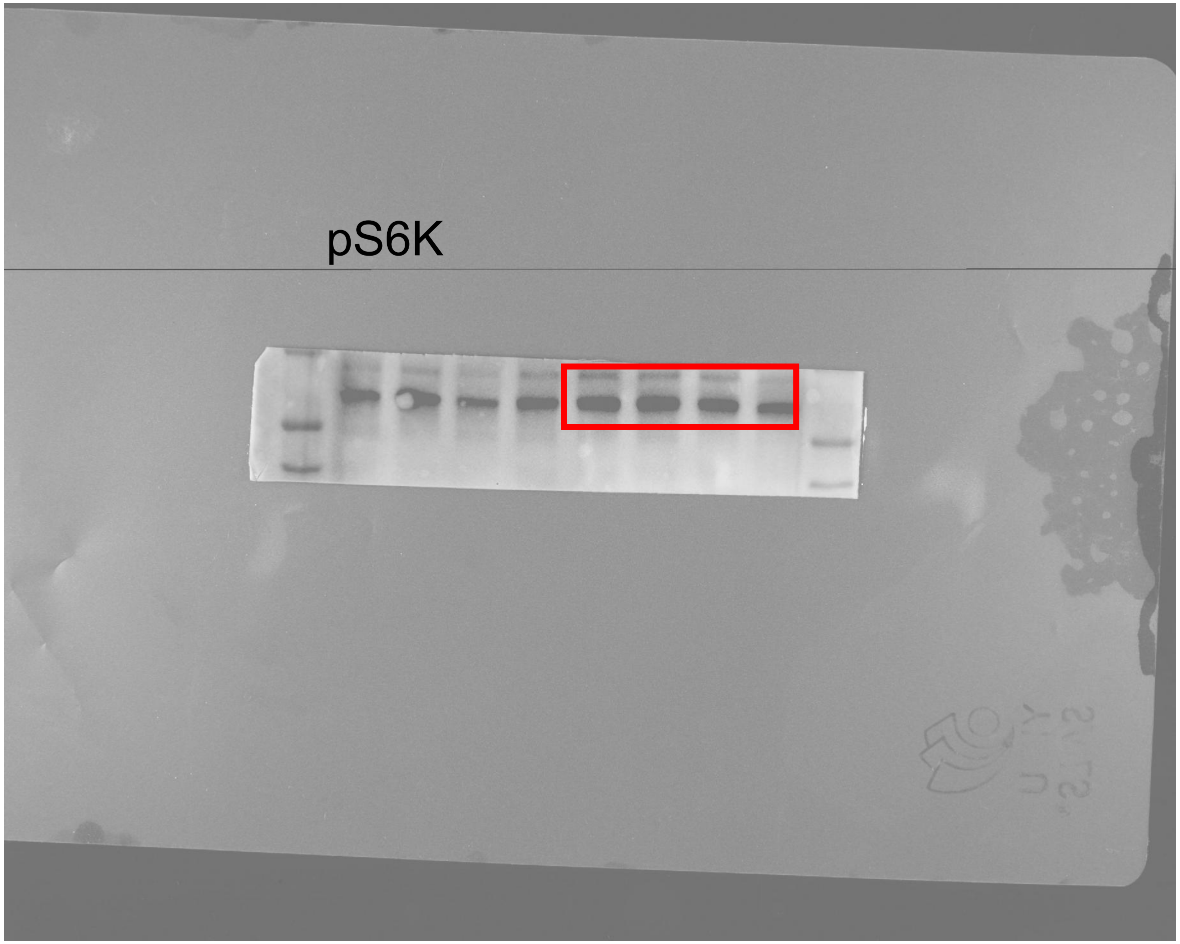

Figure 5e right

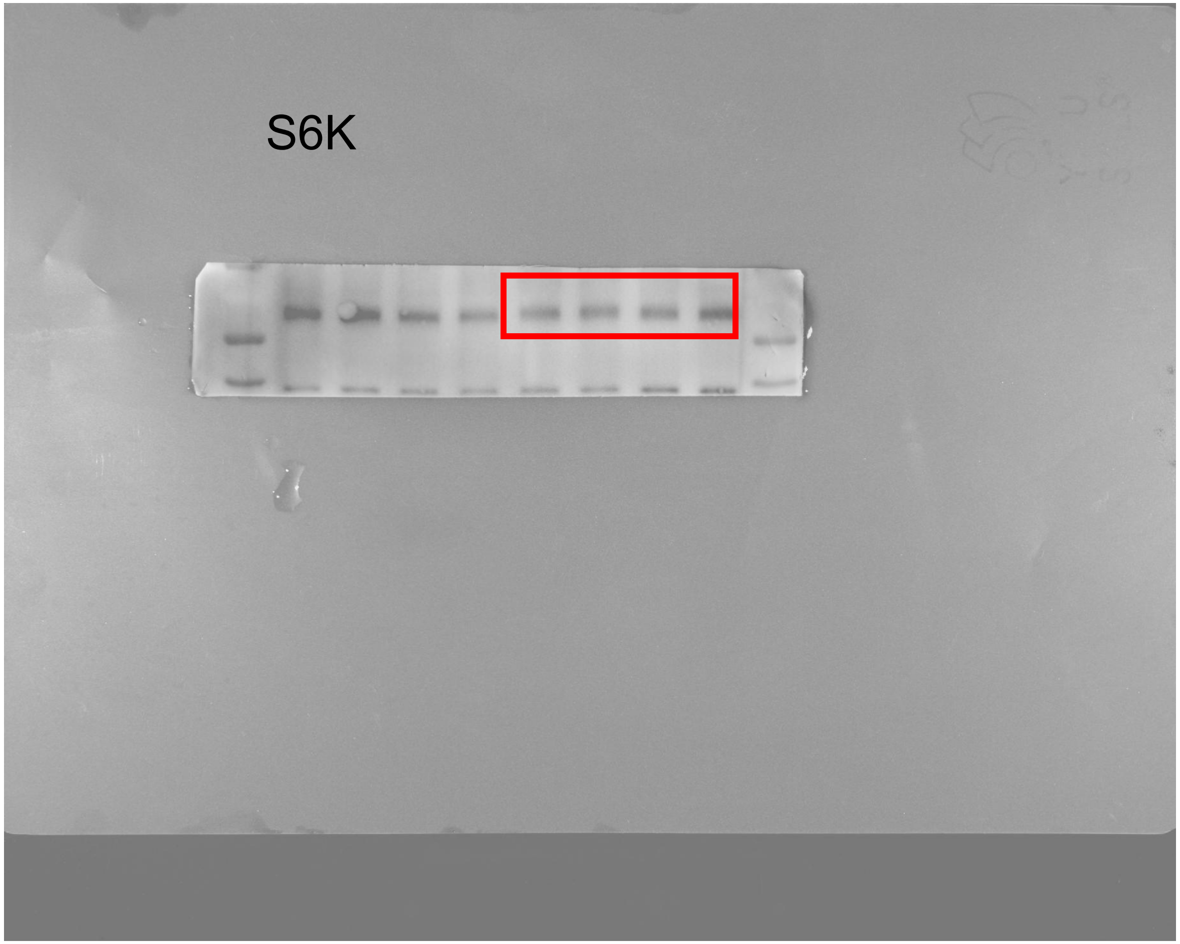

Figure 5e right

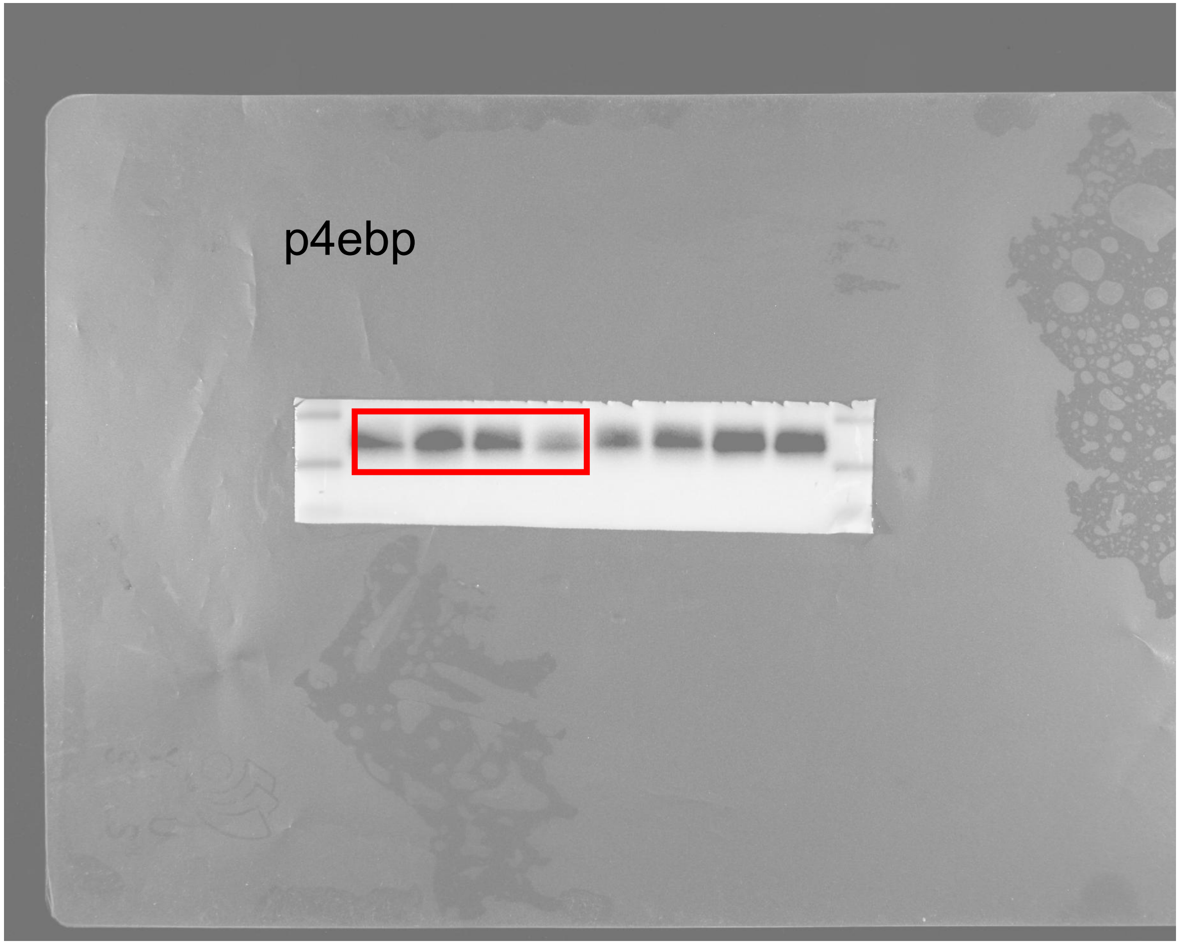

Figure 5e right

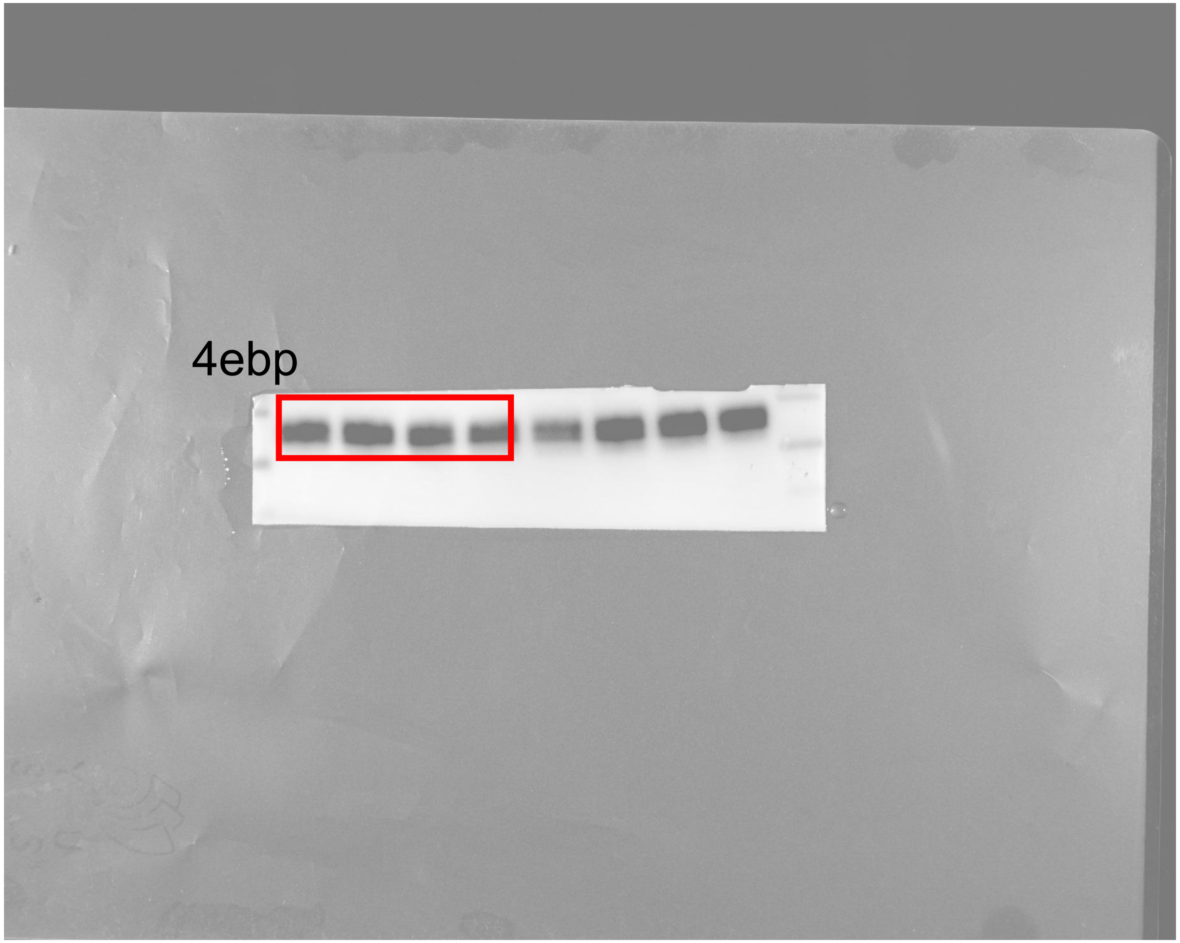

Figure 5e right

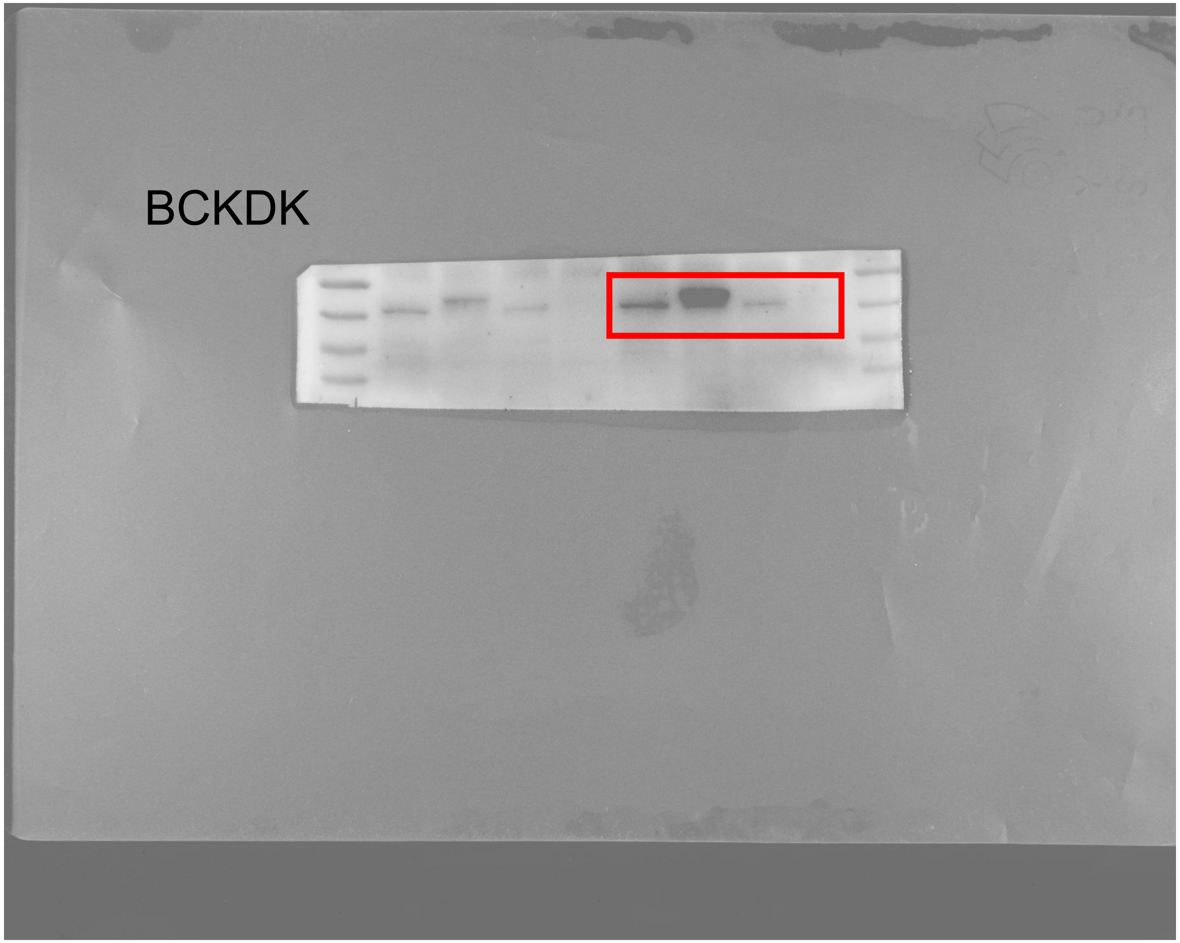

Figure 5e right

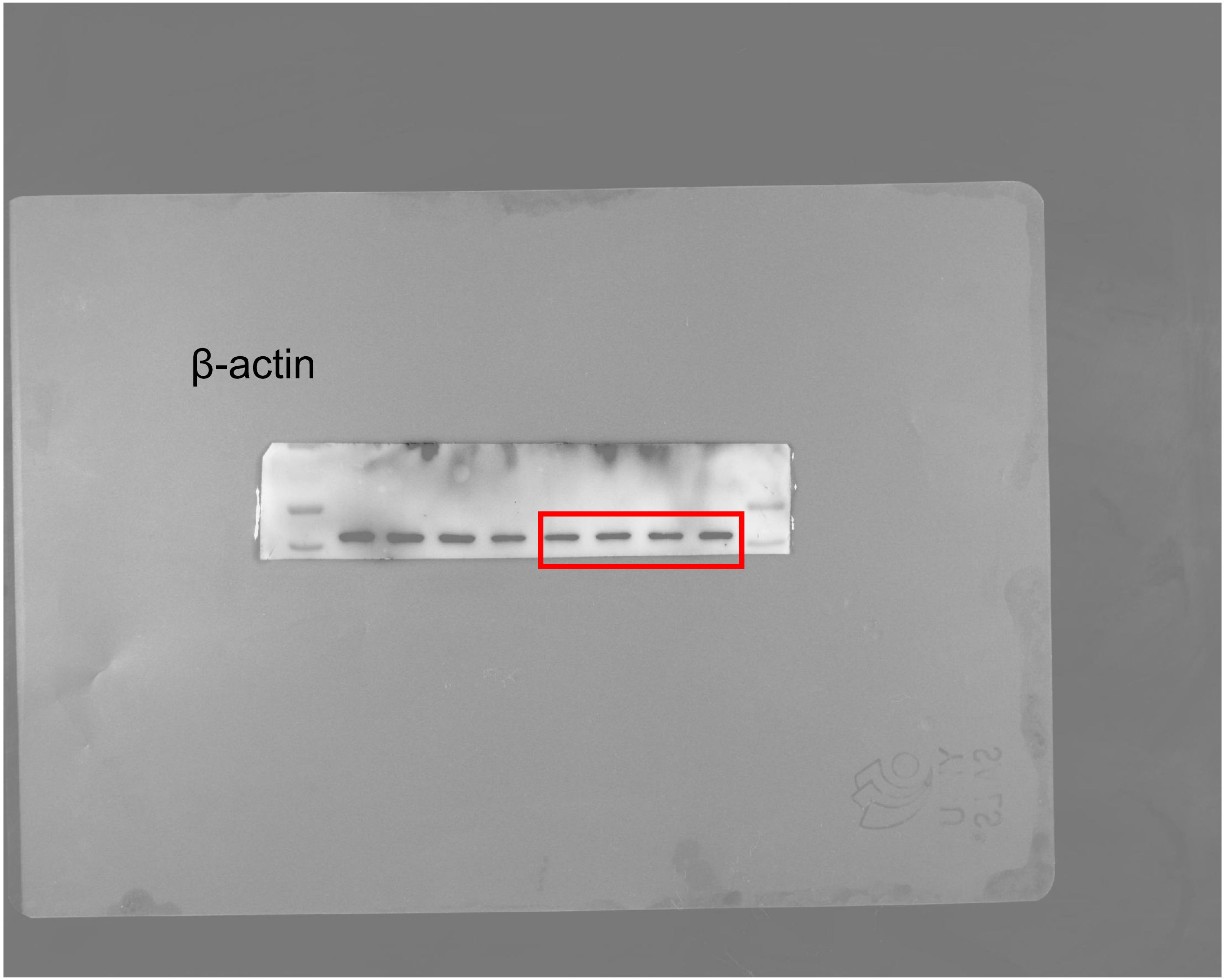

Figure 5f left

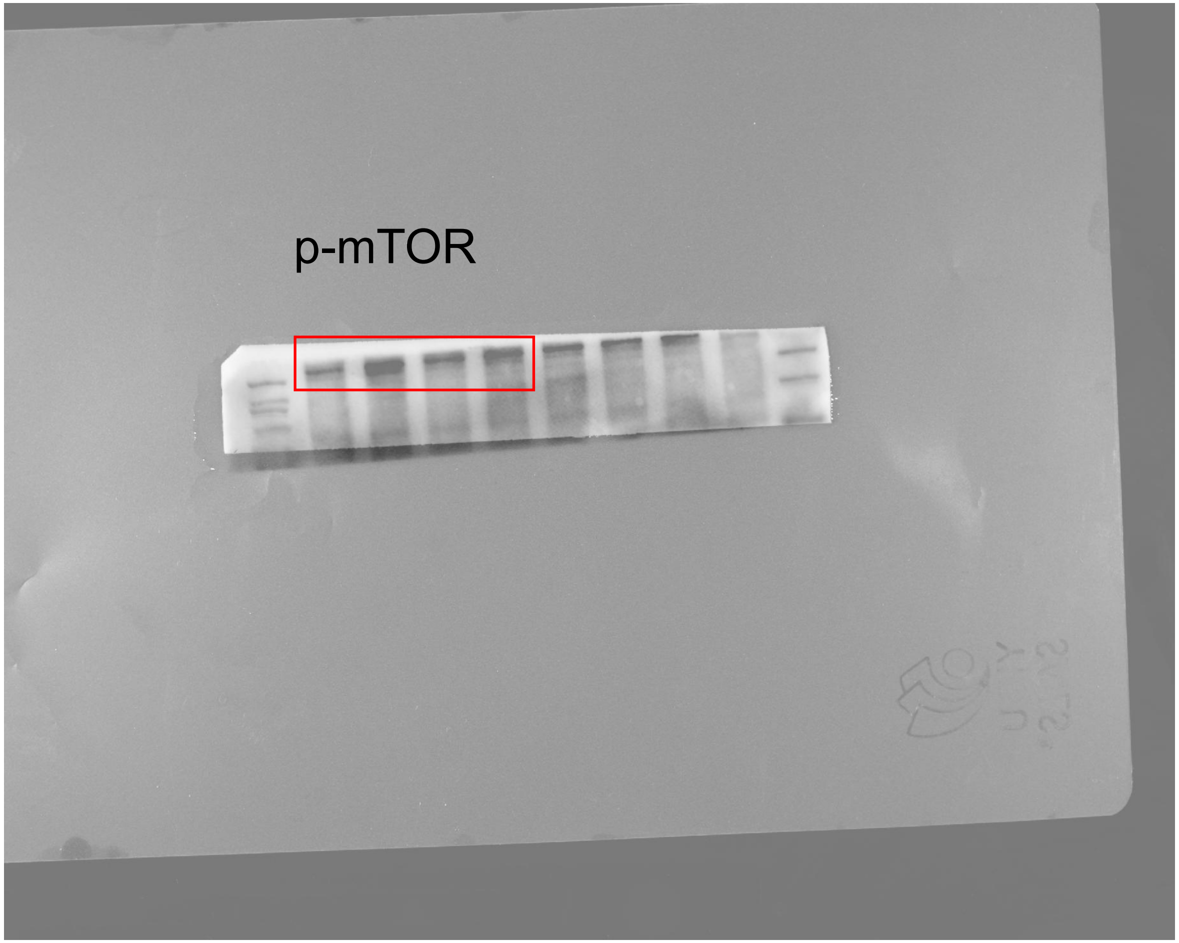

Figure 5f left

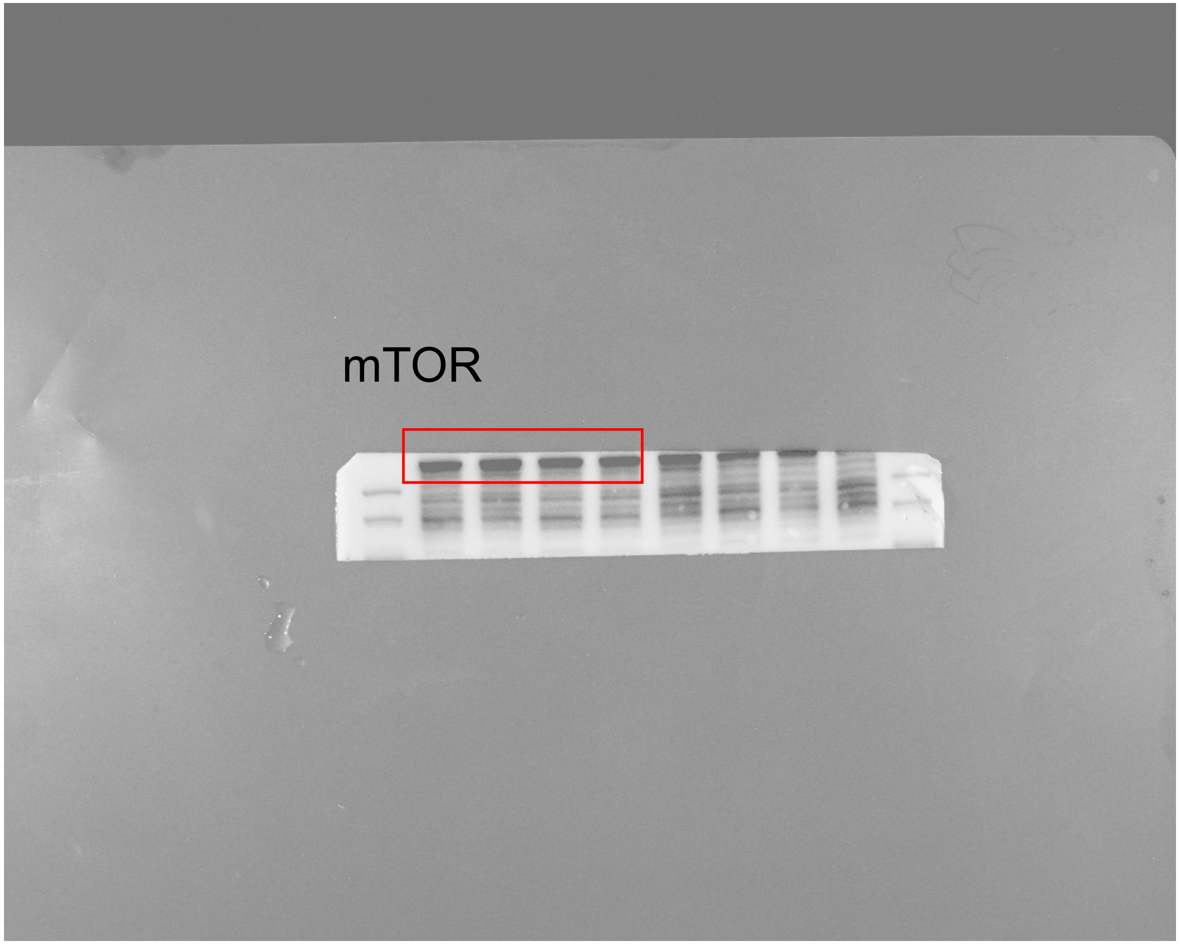

Figure 5f left

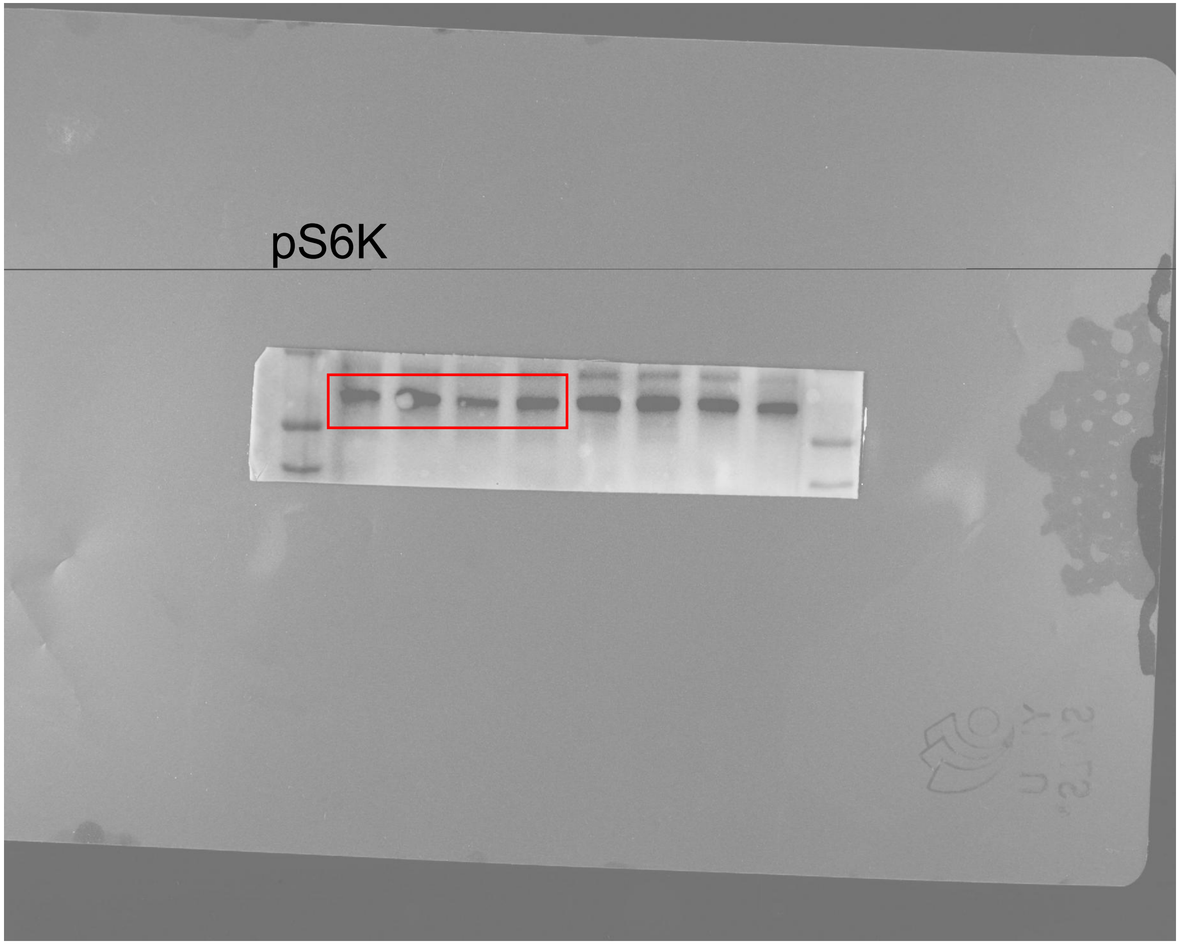

Figure 5f left

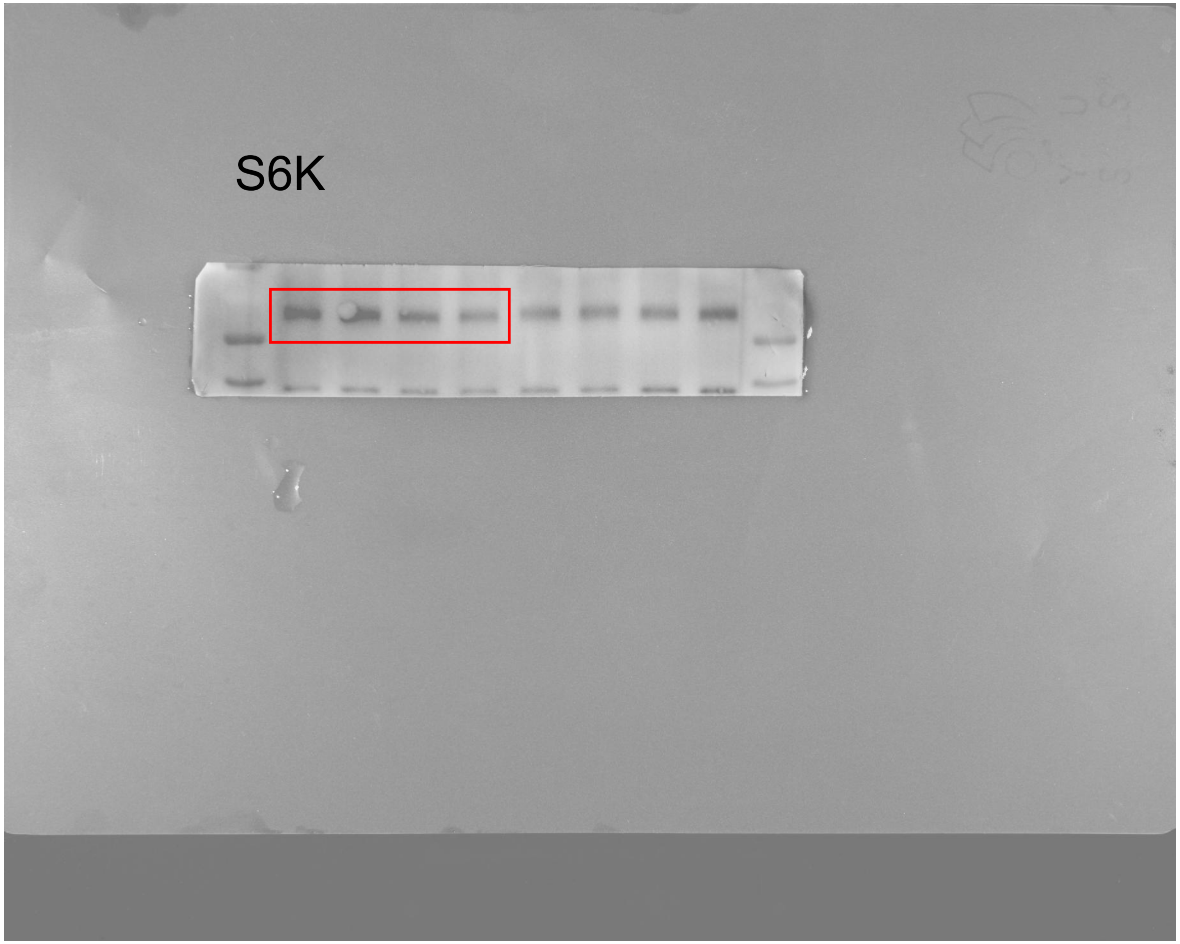

Figure 5f left

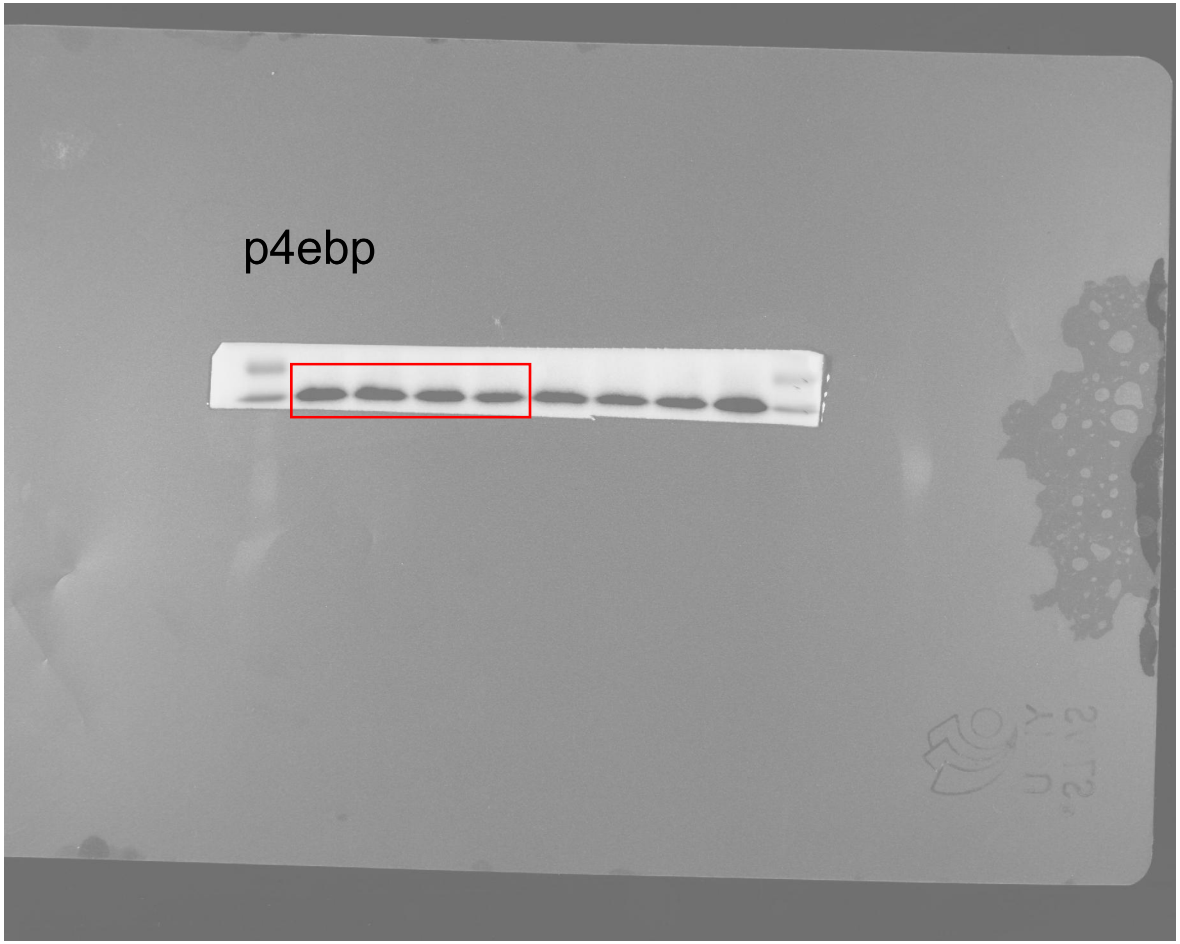

Figure 5f left

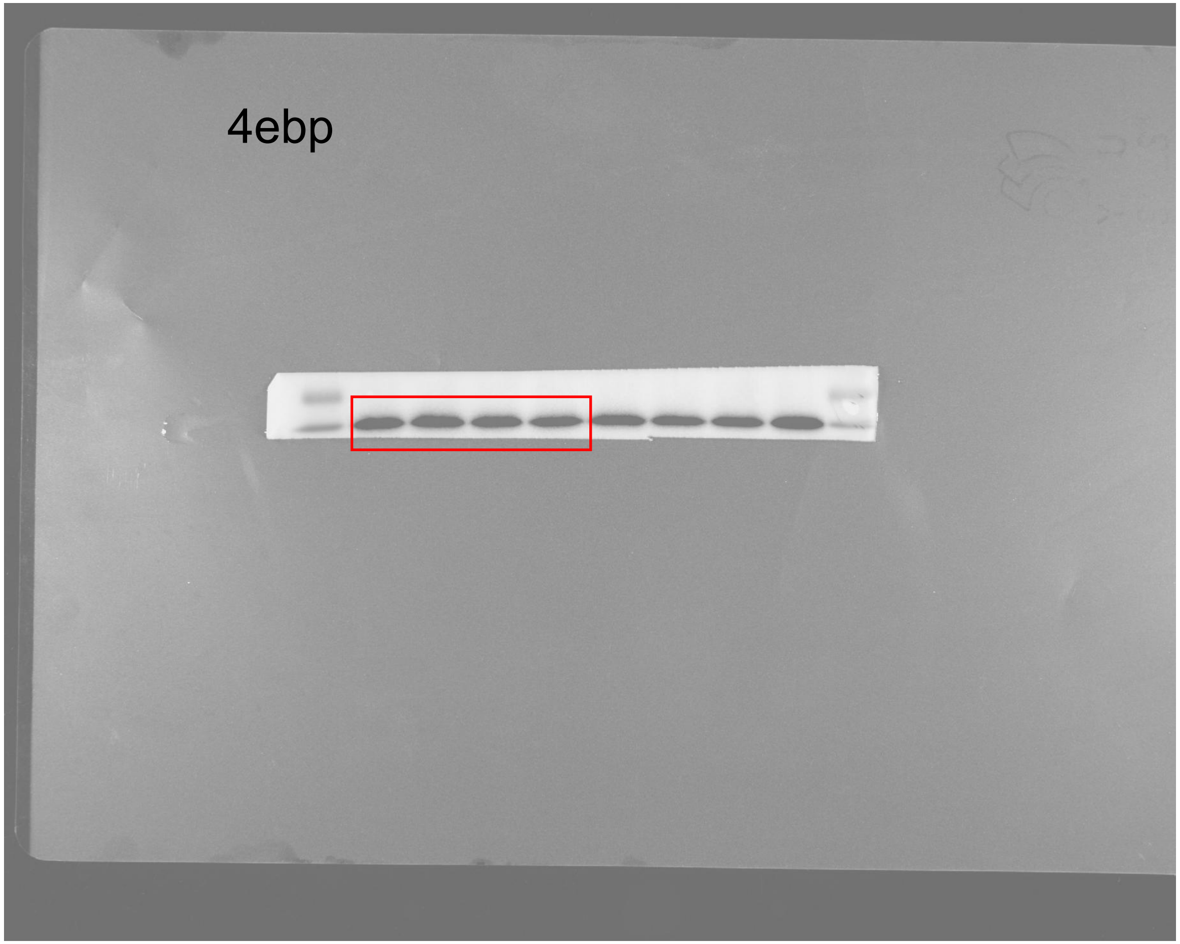

Figure 5f left

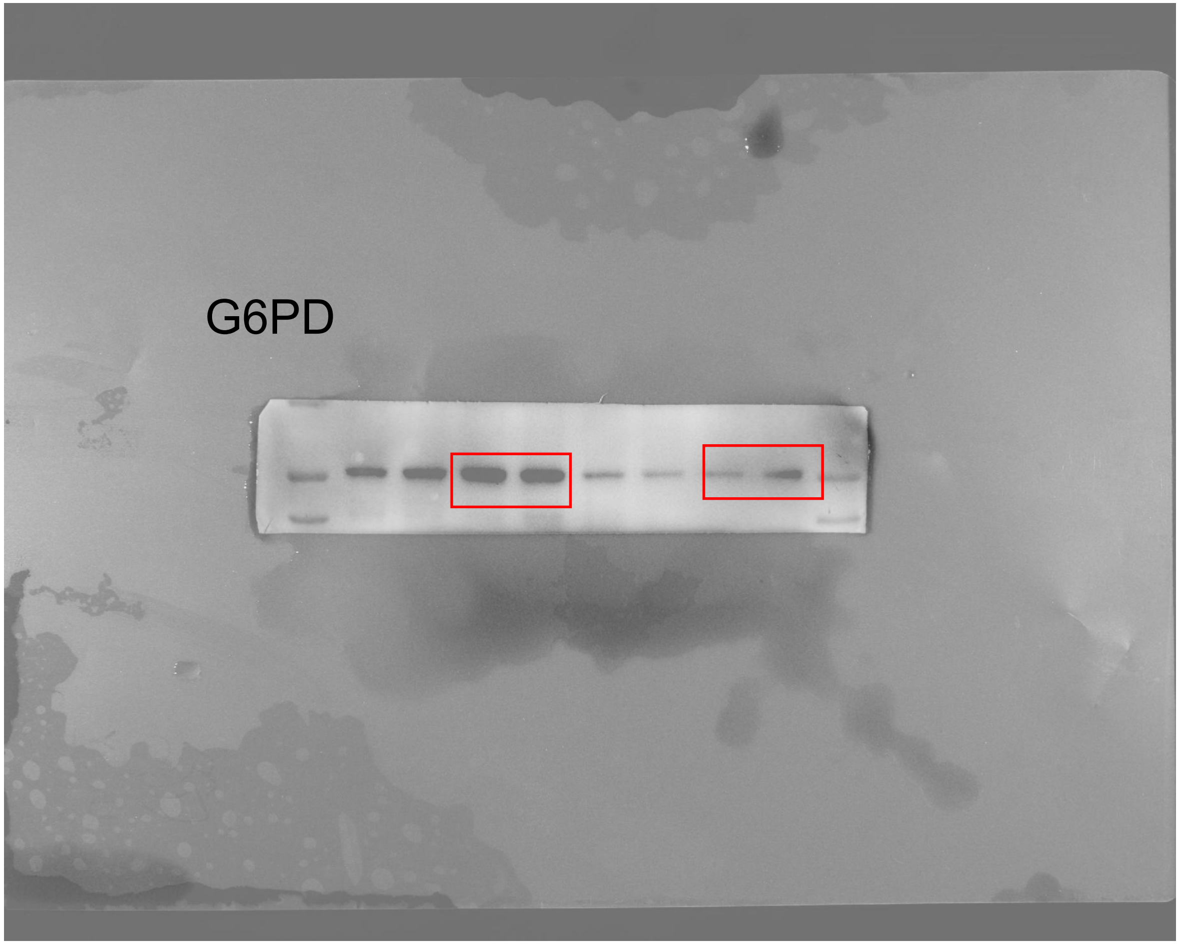

Figure 5f left

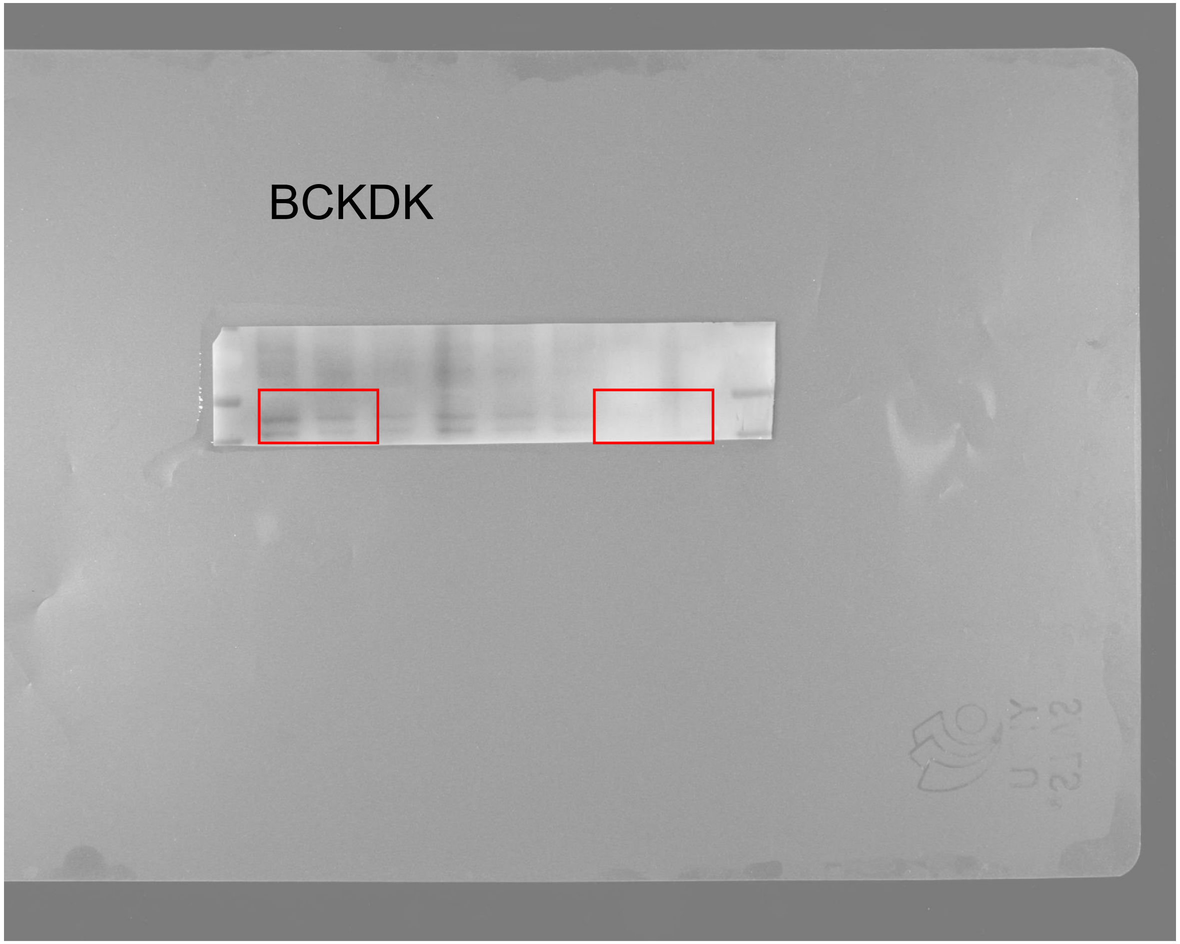

Figure 5f left

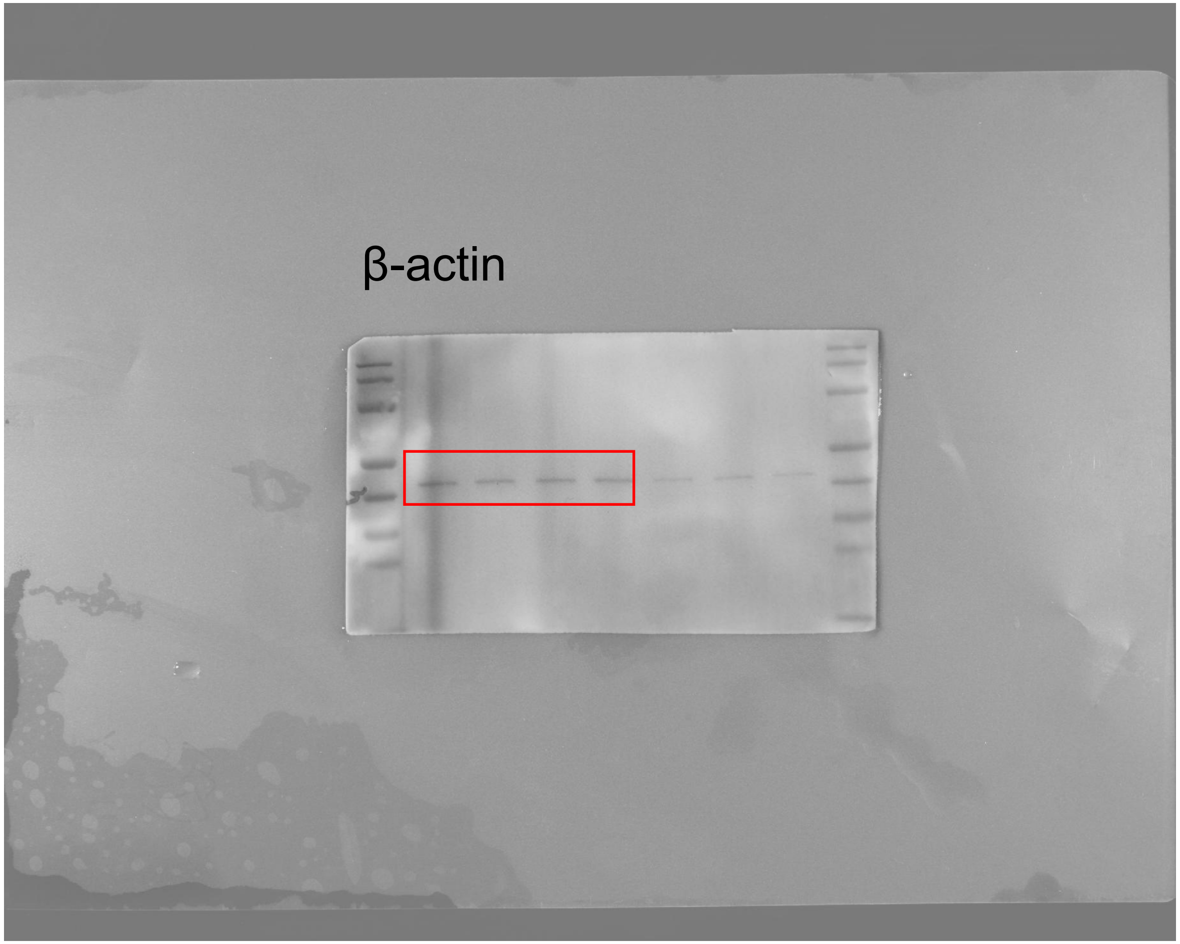

Figure 5f right

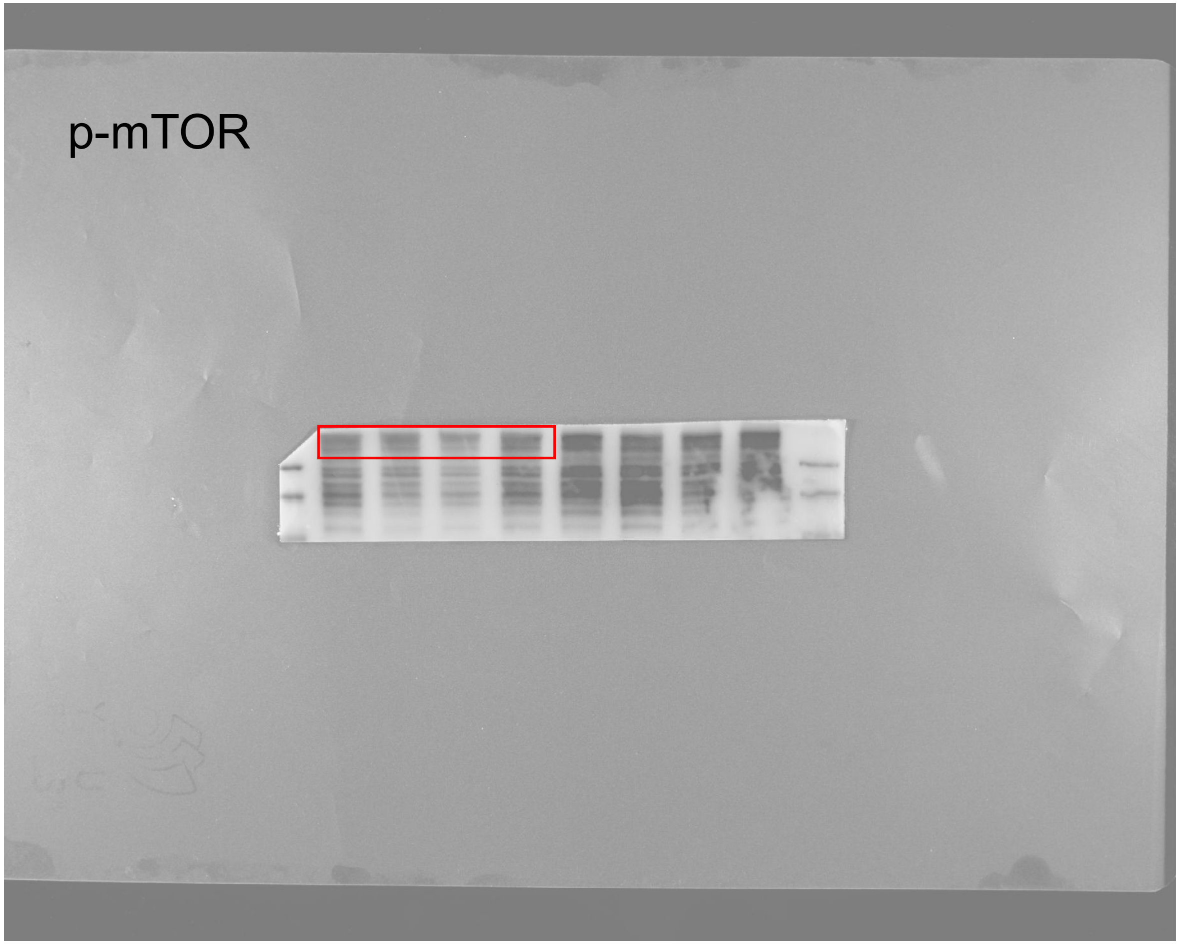

Figure 5f right

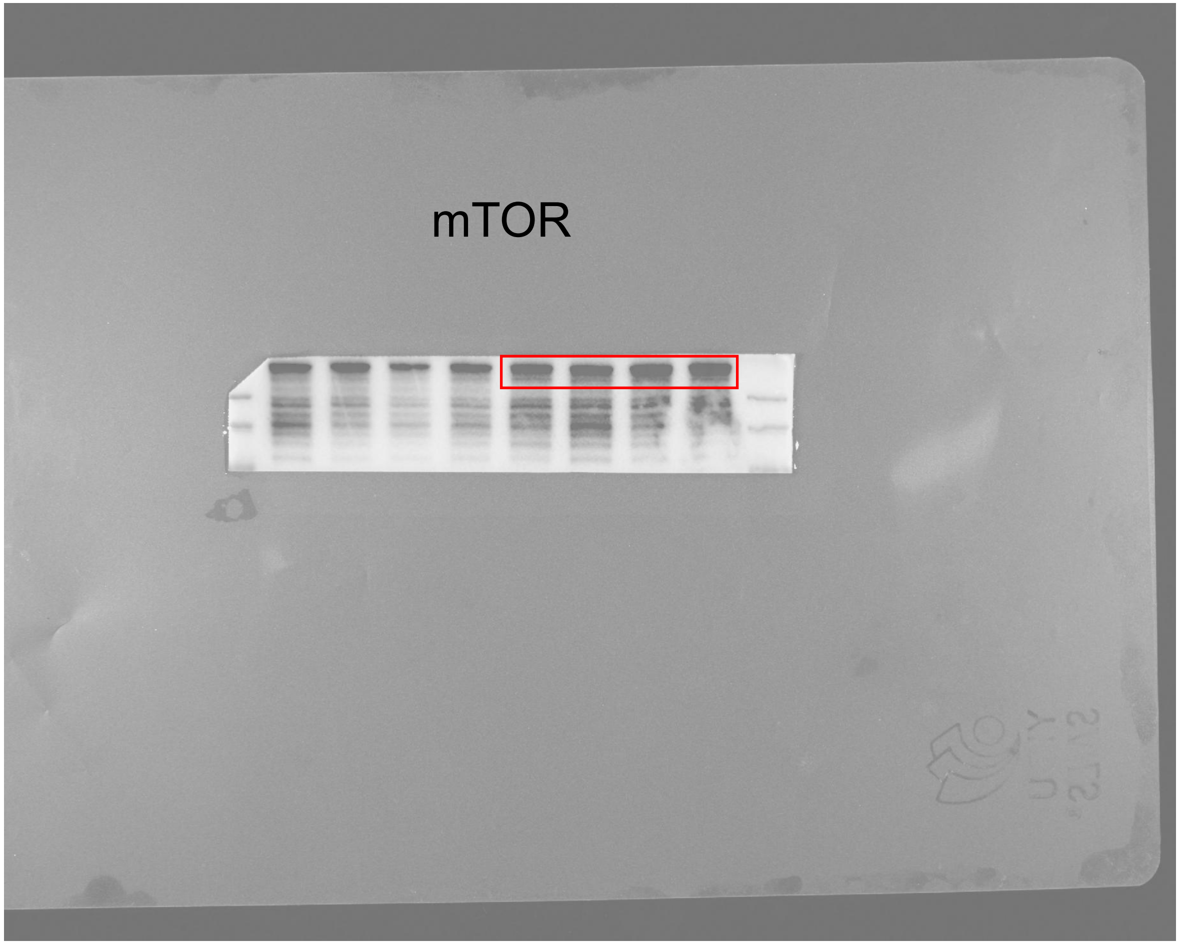

Figure 5f right

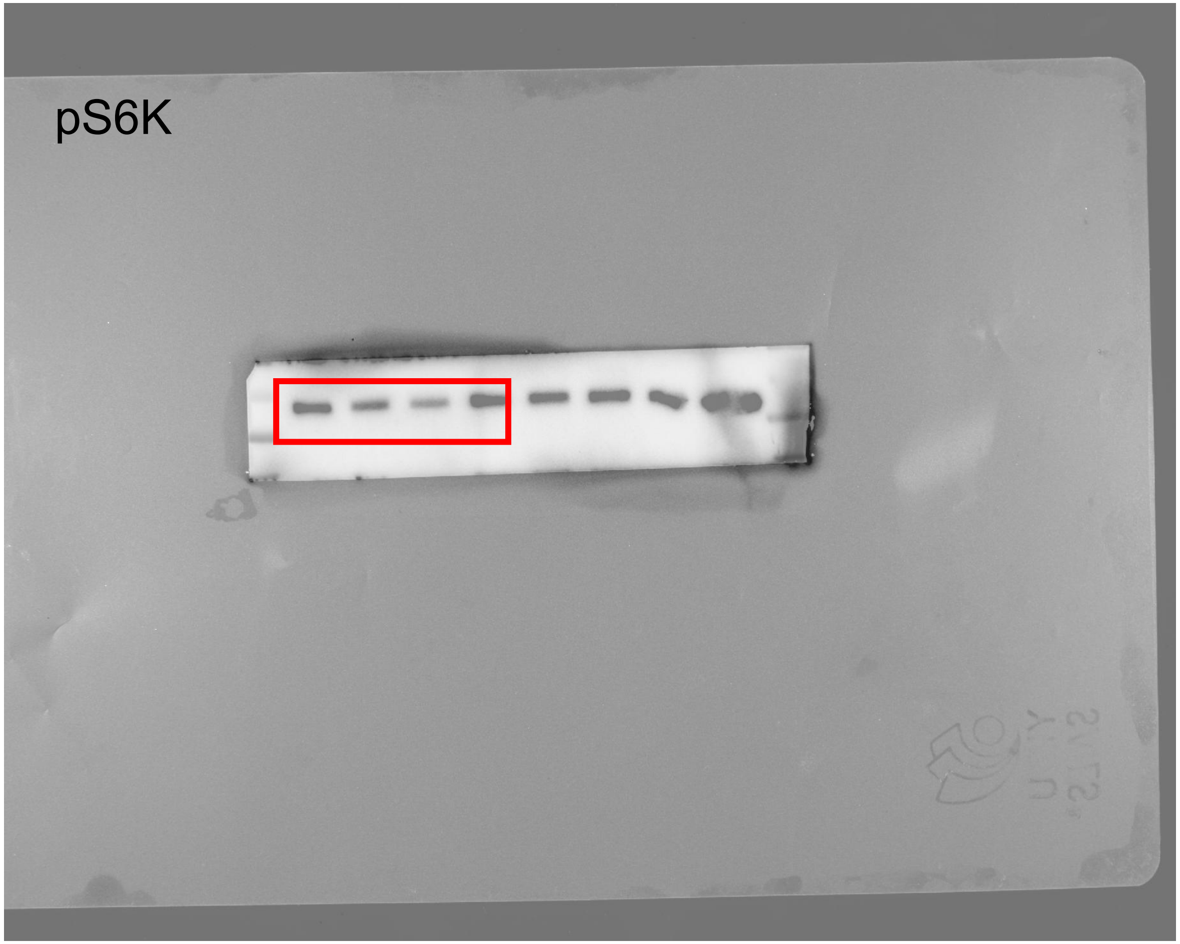

Figure 5f right

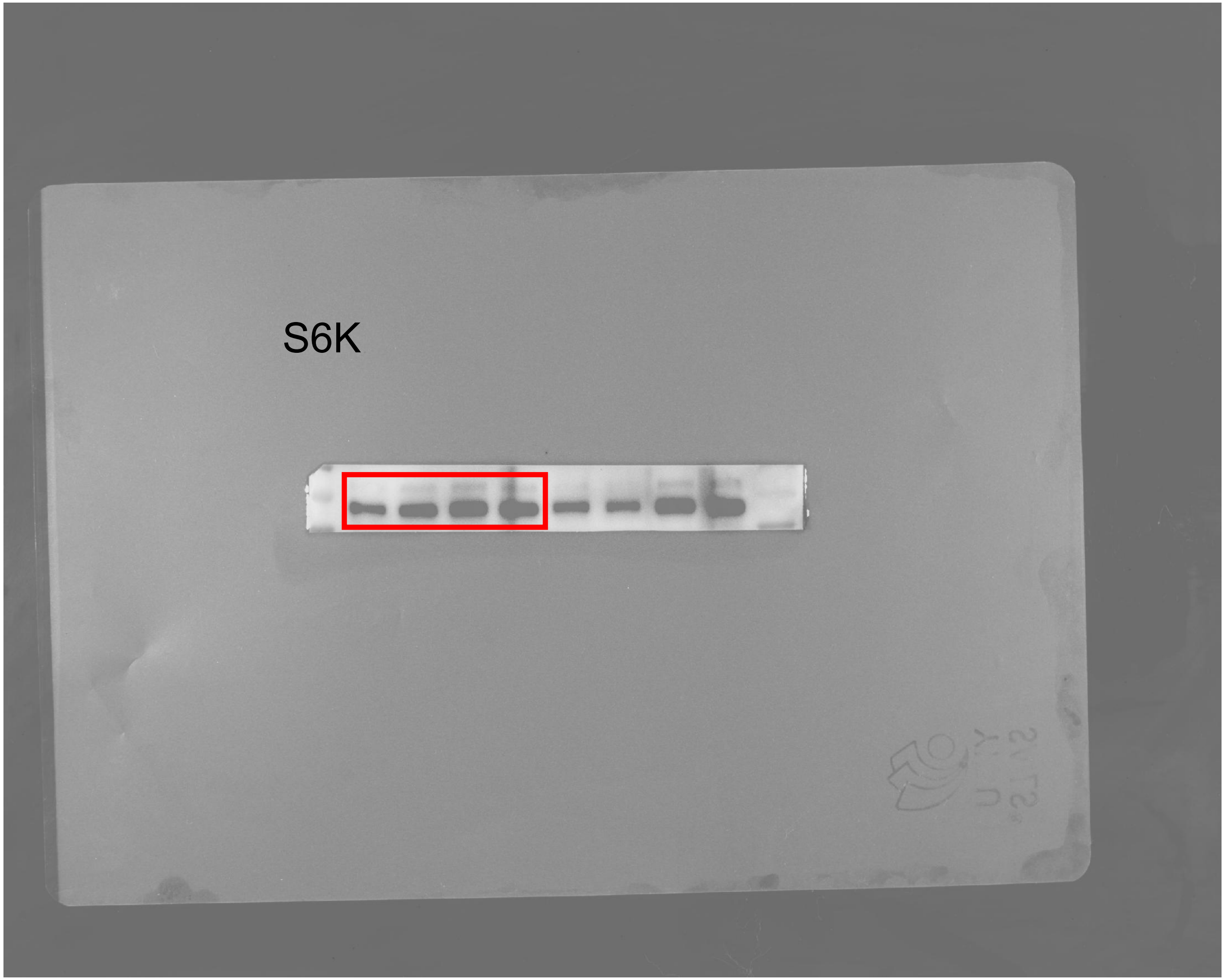

Figure 5f right

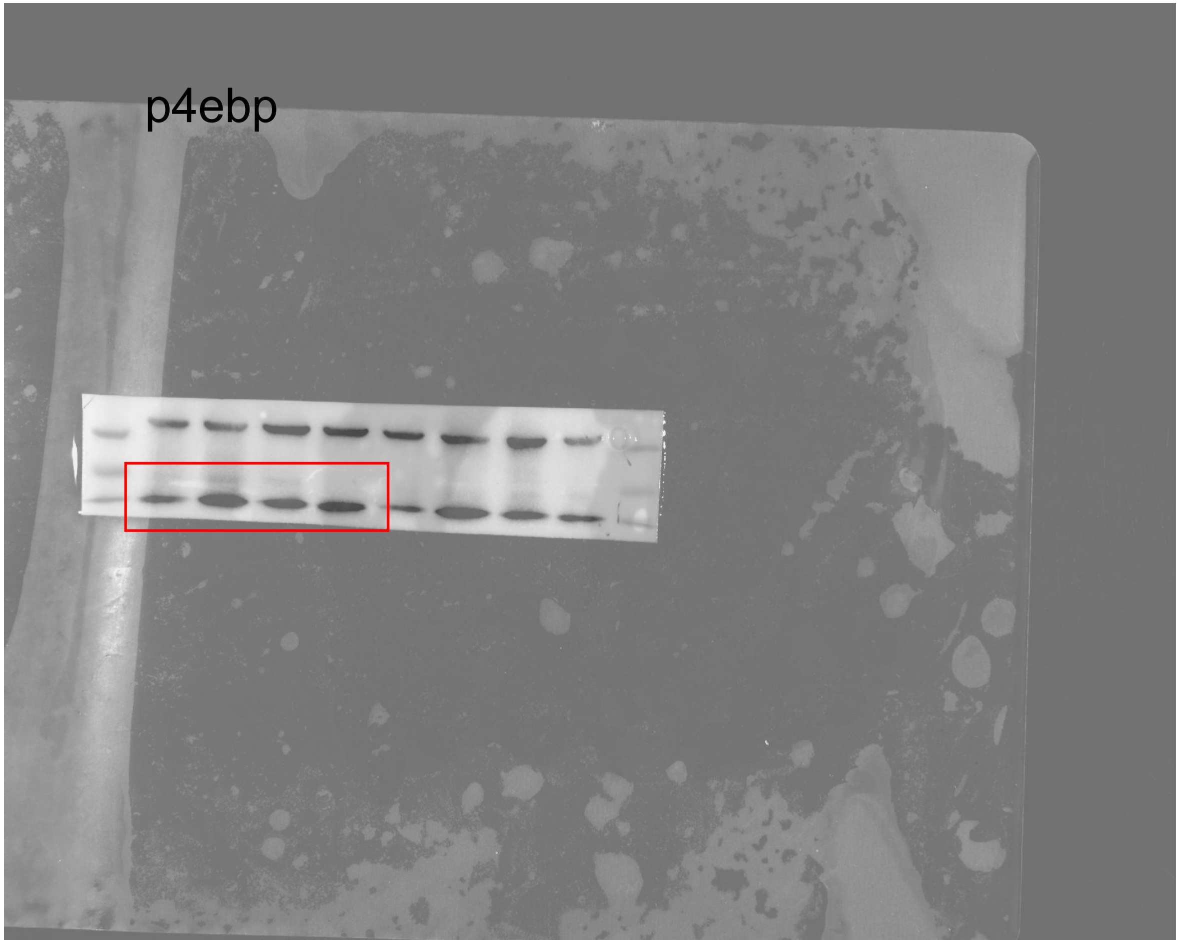

Figure 5f right

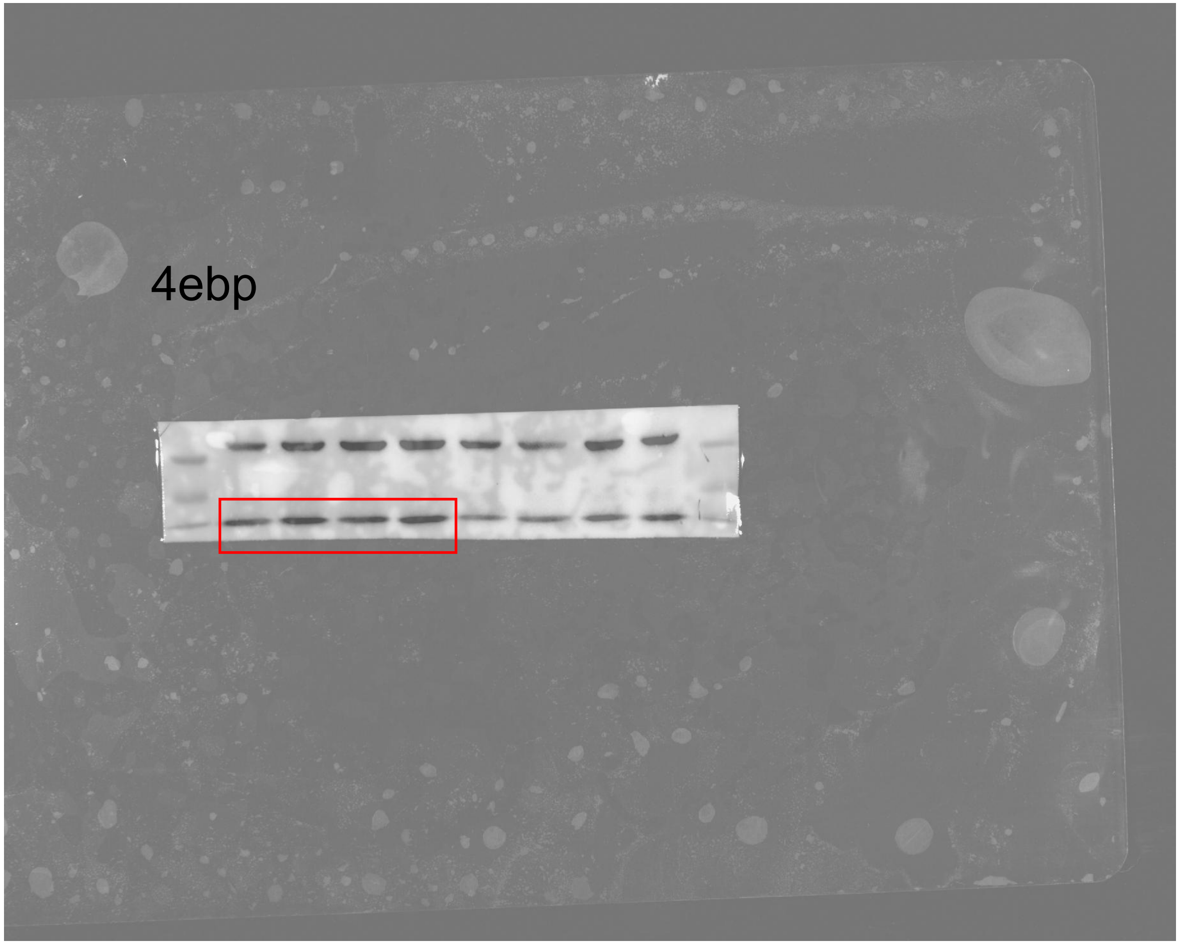

Figure 5f right

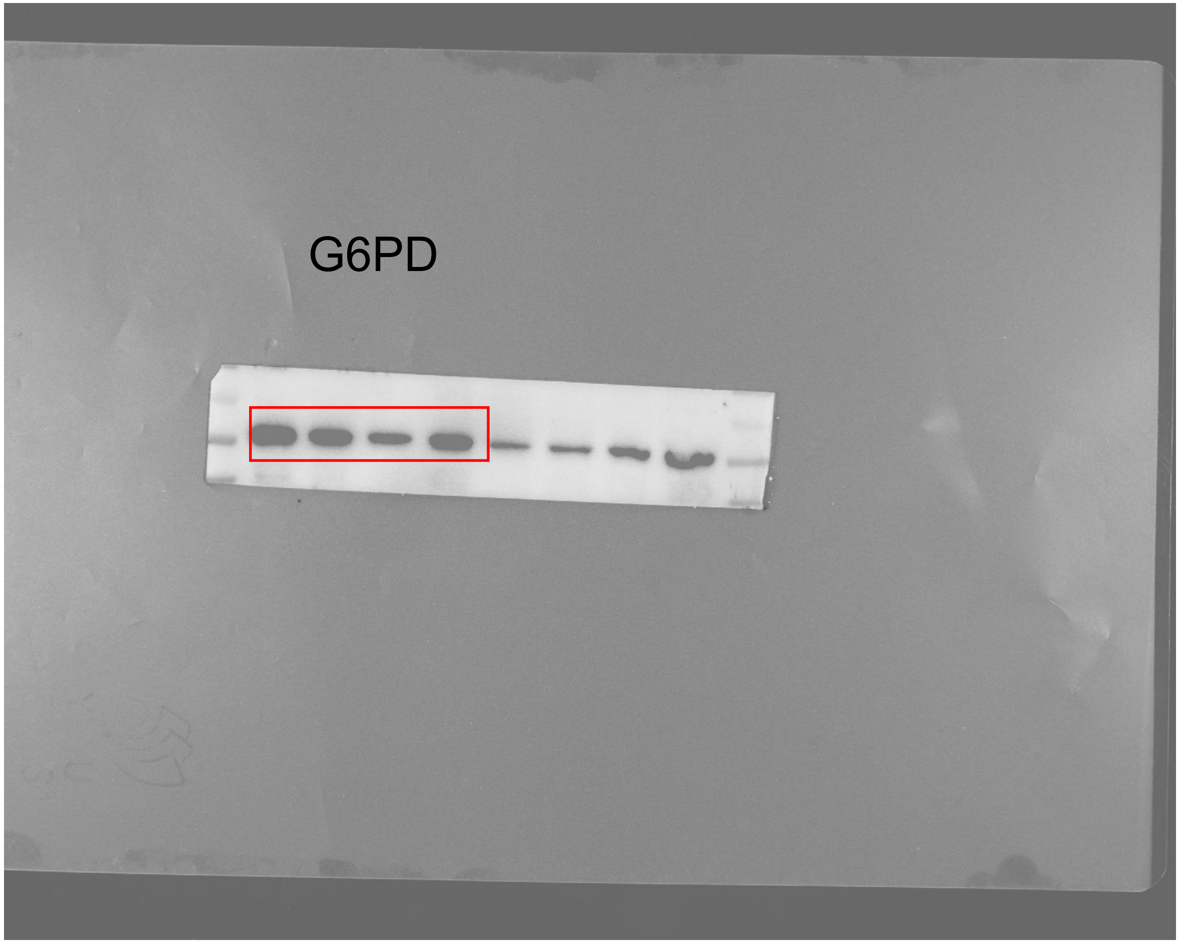

Figure 5f right

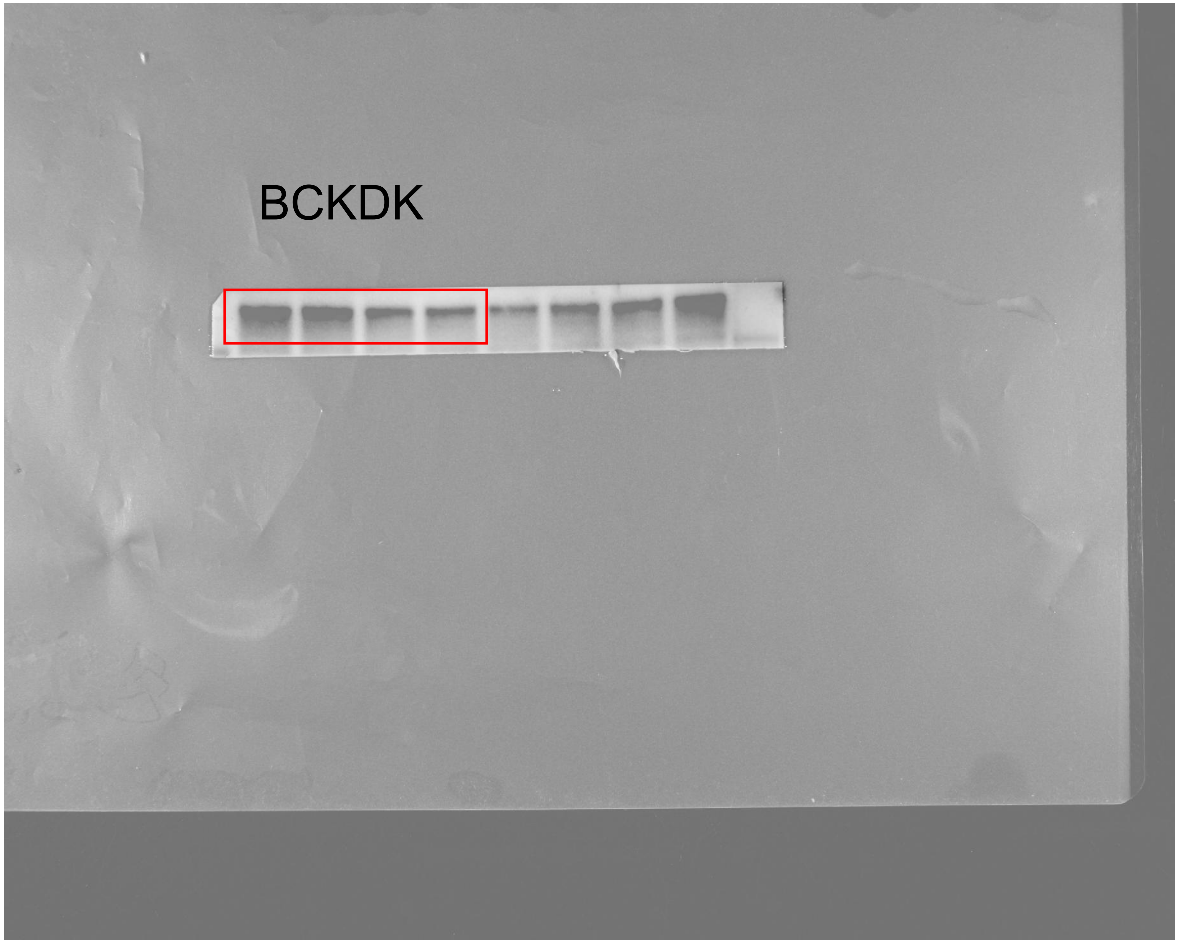

Figure 5f right

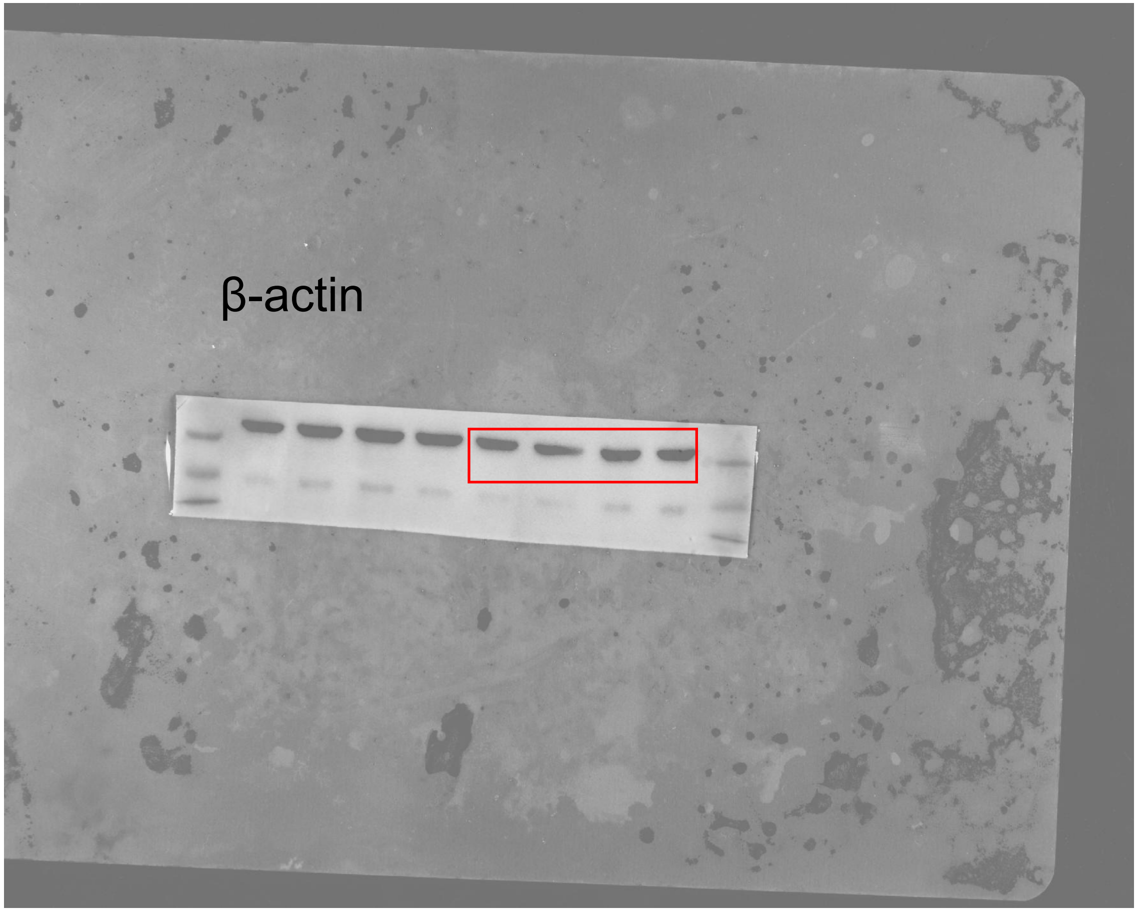

Figure 7a upper

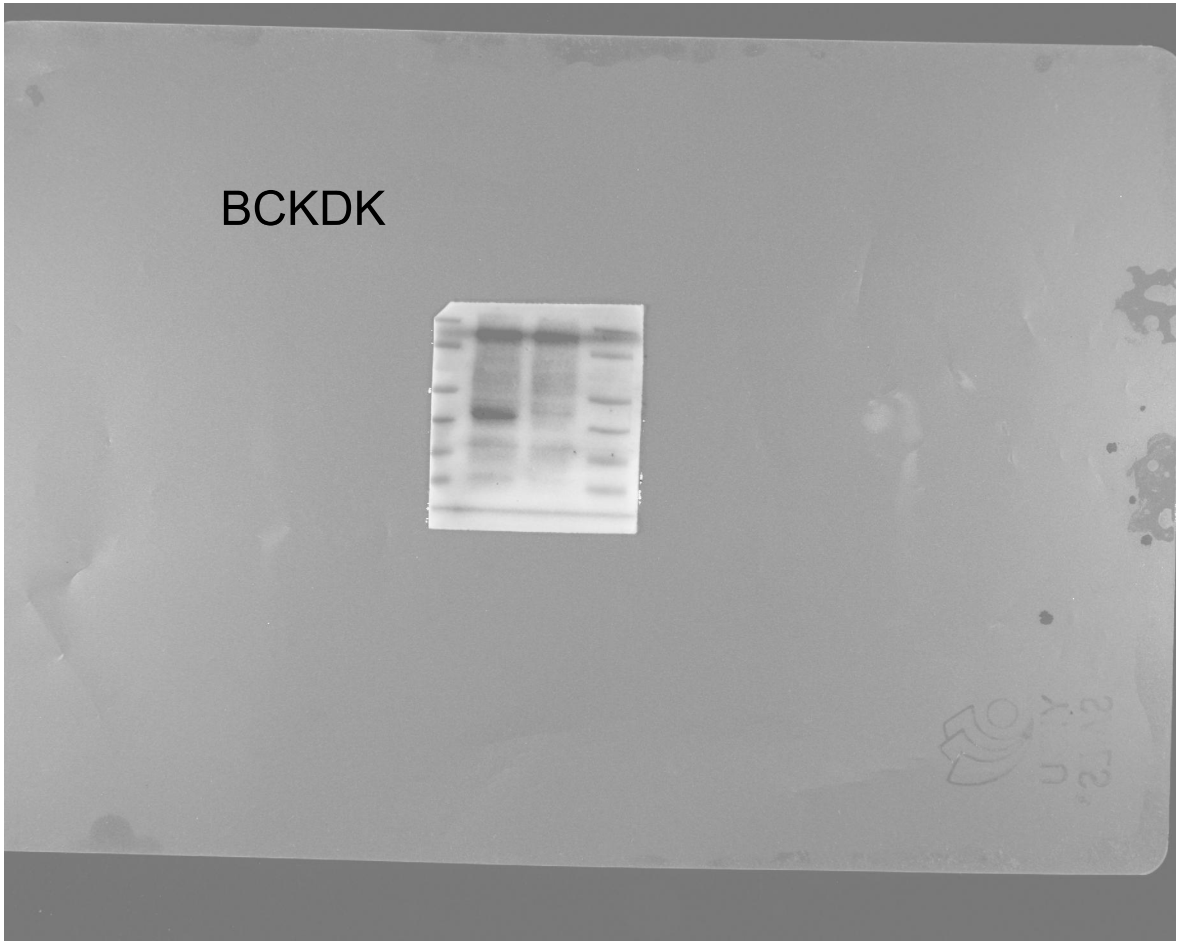

Figure 7a upper

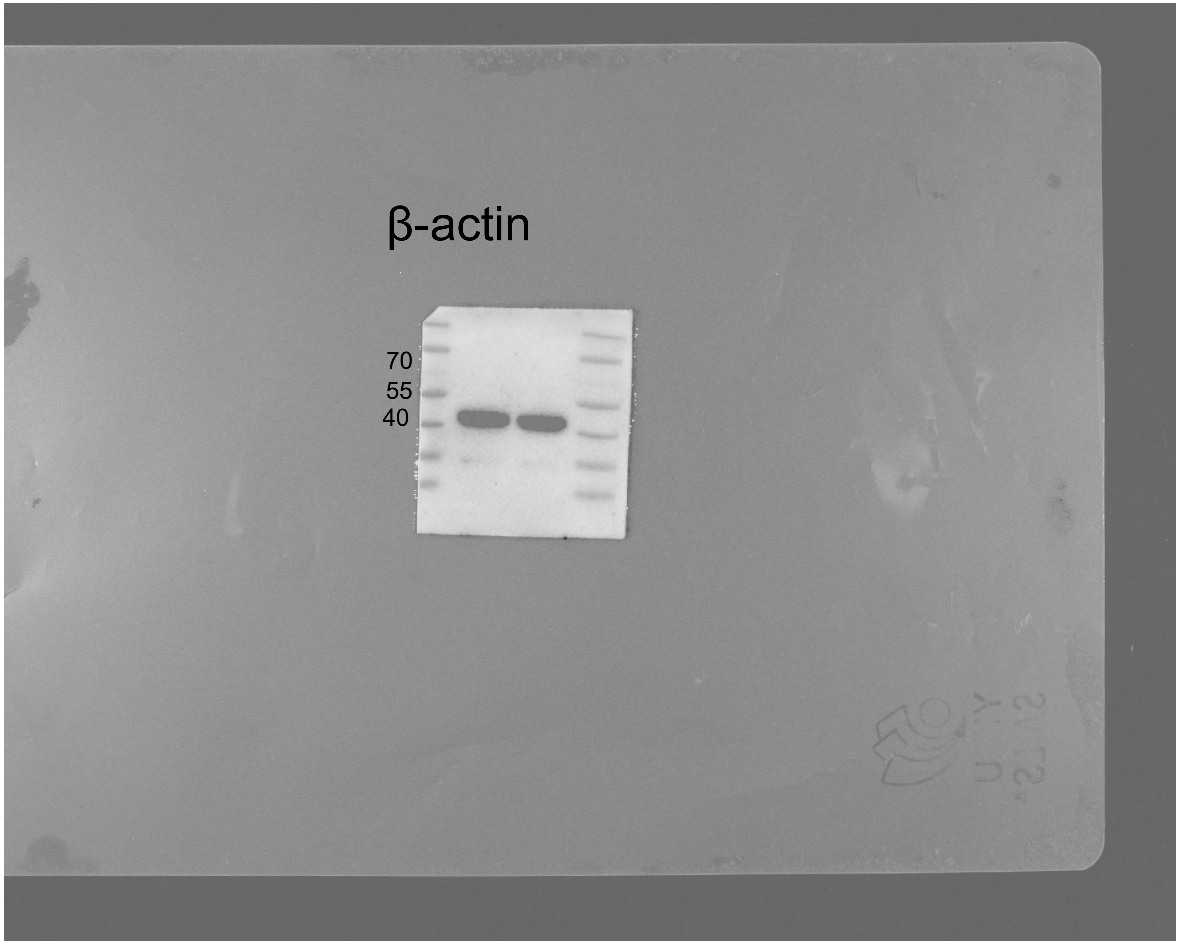

Figure 7a lower

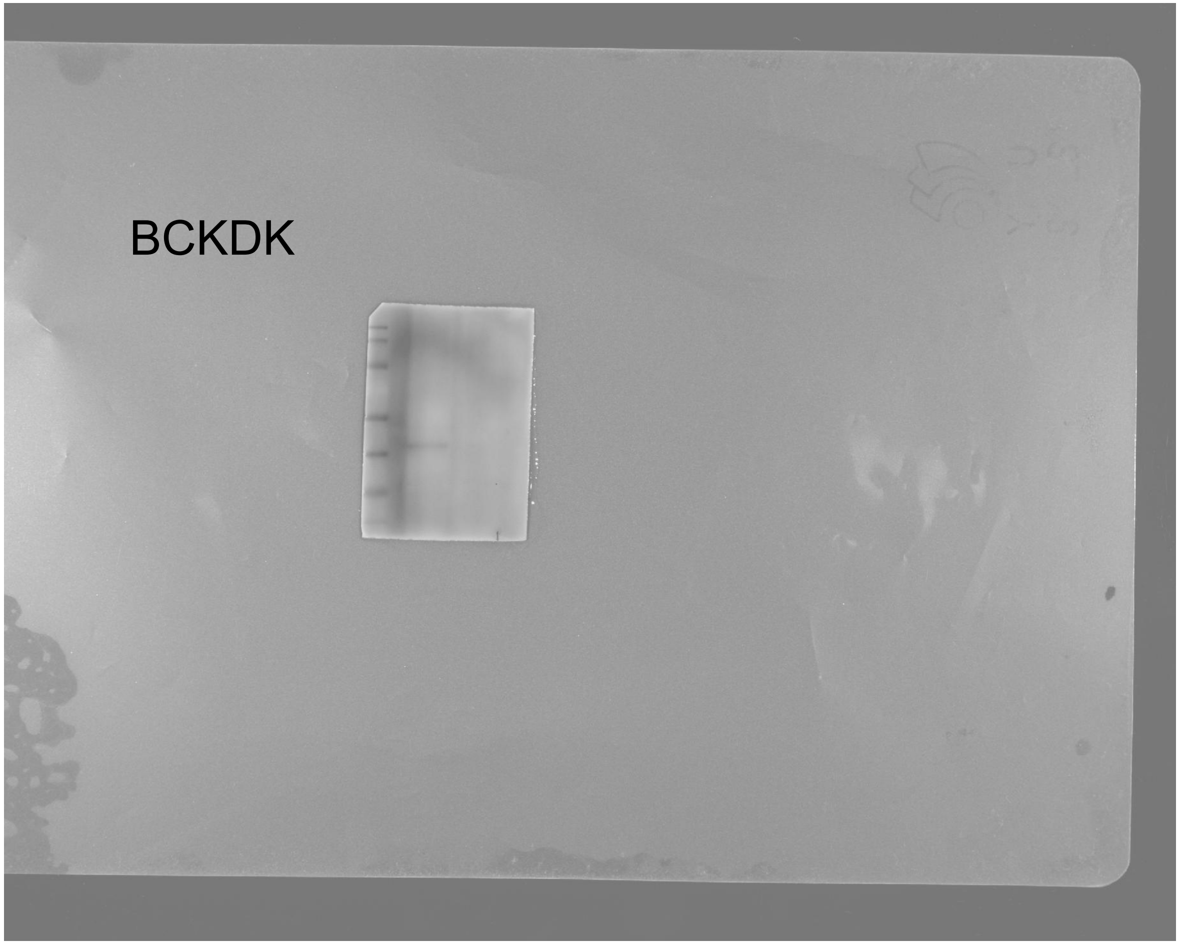

Figure 7a lower

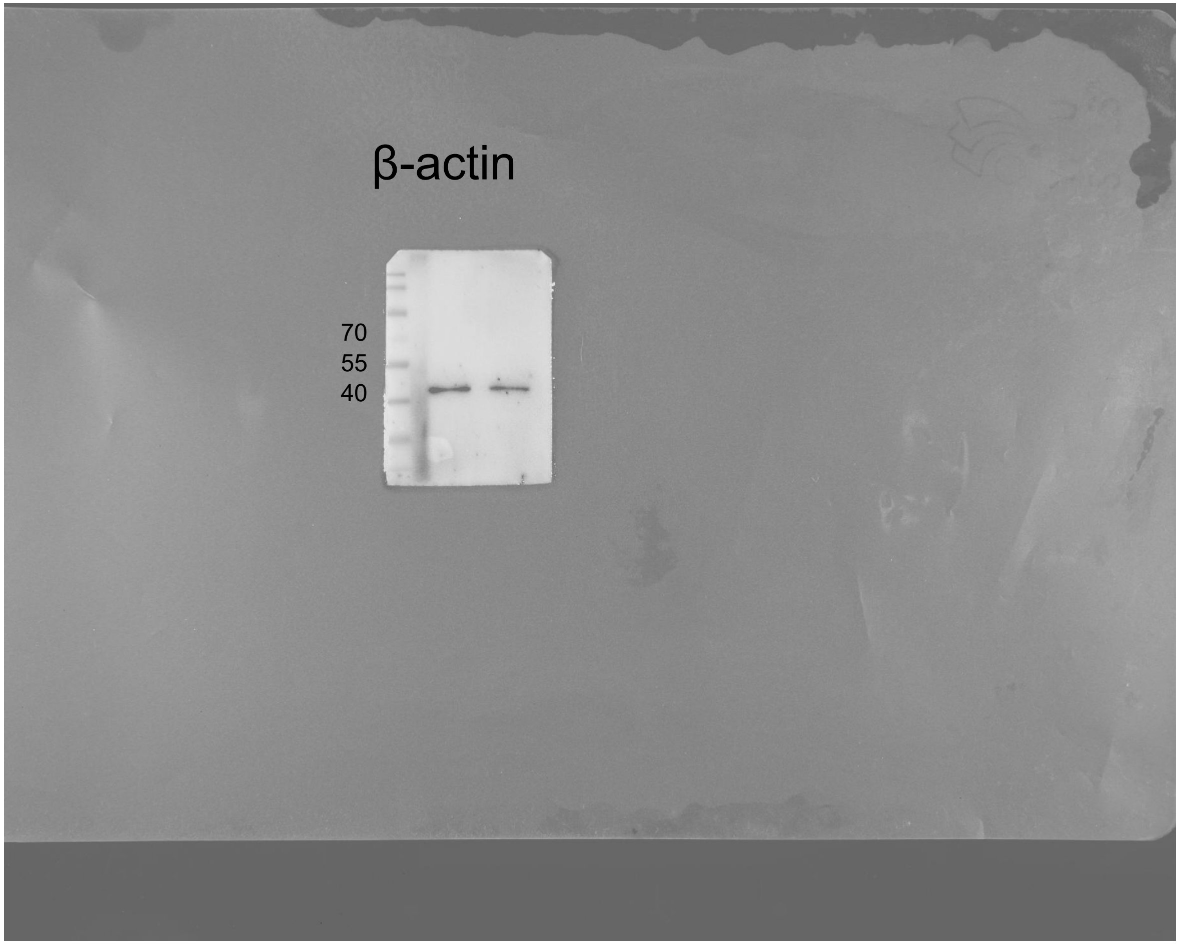

Figure S1a

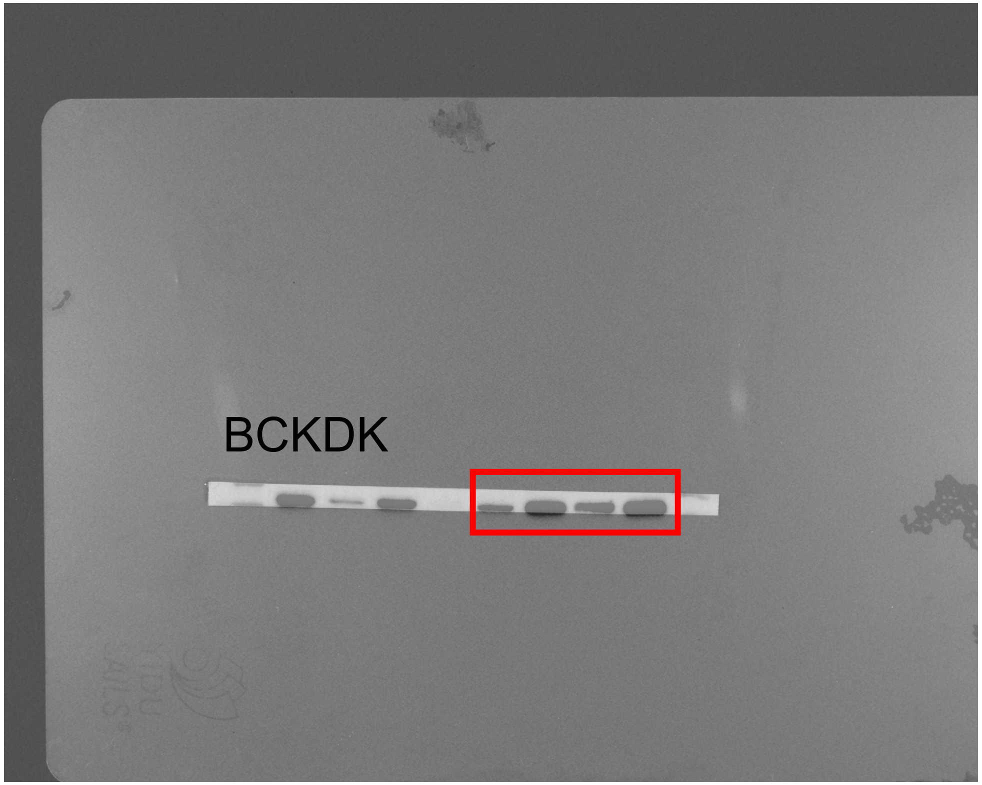

Figure S1a

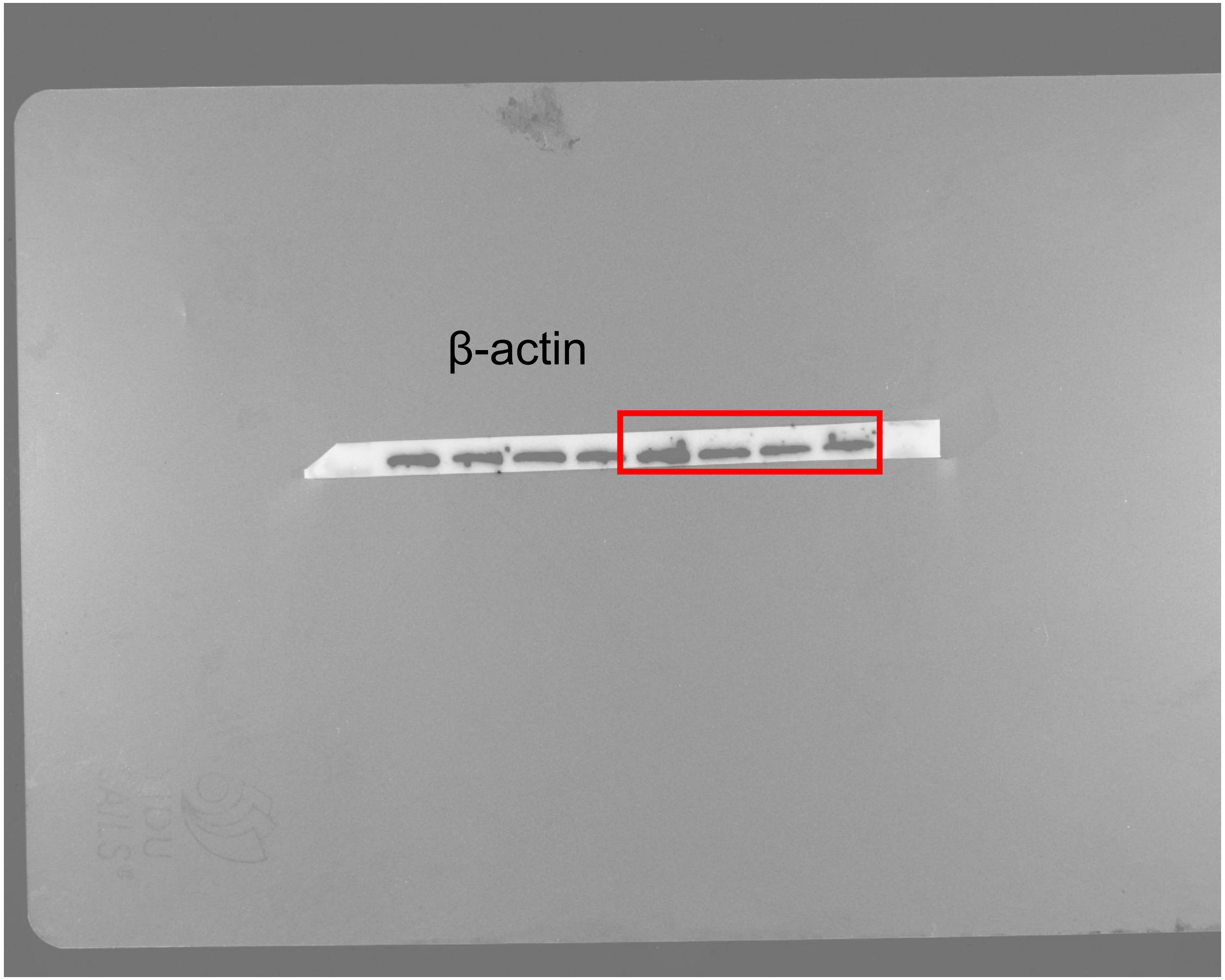

Figure S5

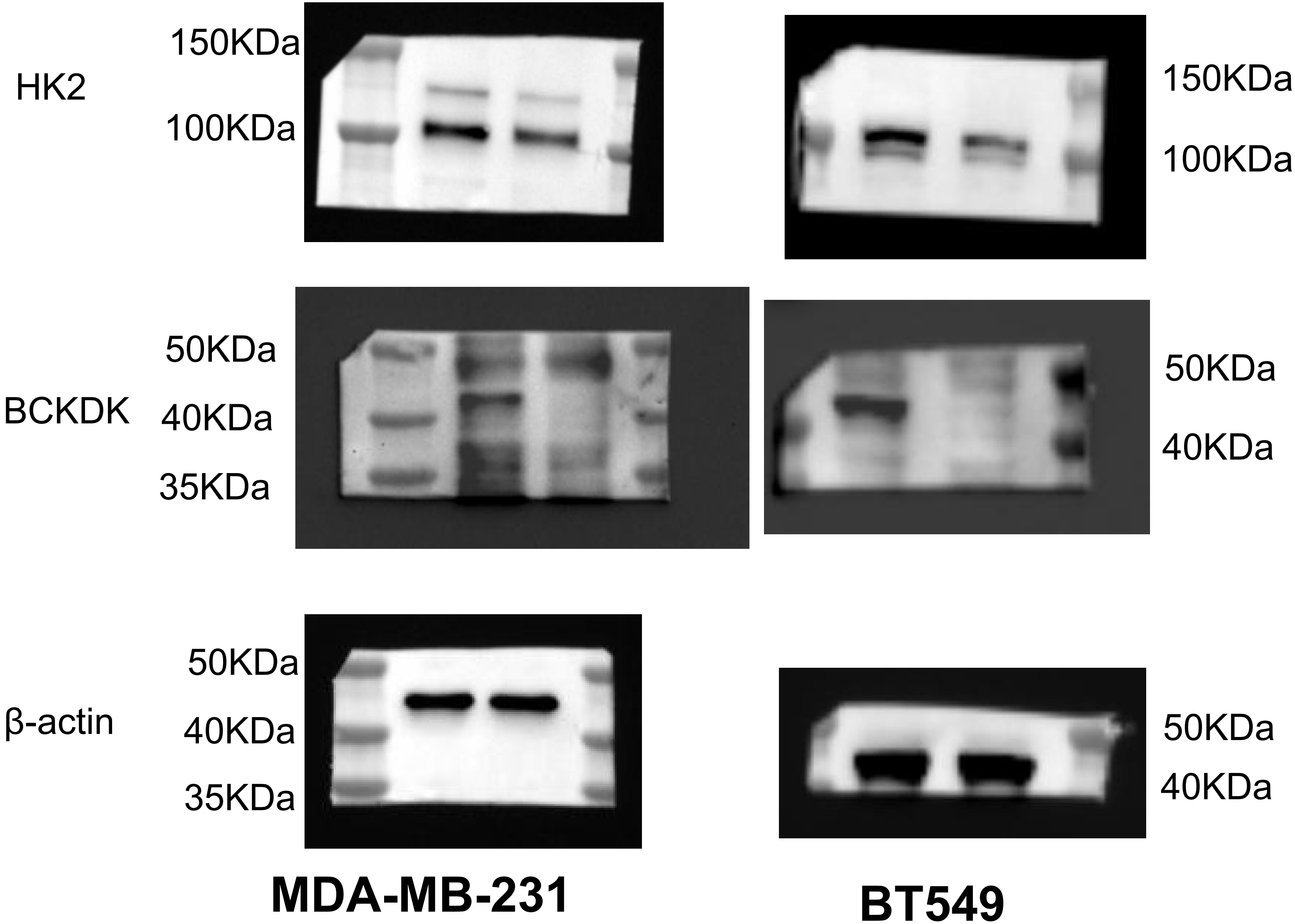
HK2
150KDa
100KDa
150KDa
100KDa
BCKDK
50KDa
40KDa
35KDa
50KDa
40KDa
β-actin
50KDa
40KDa
35KDa
50KDa
40KDa
MDA-MB-231
BT549

Figure S5

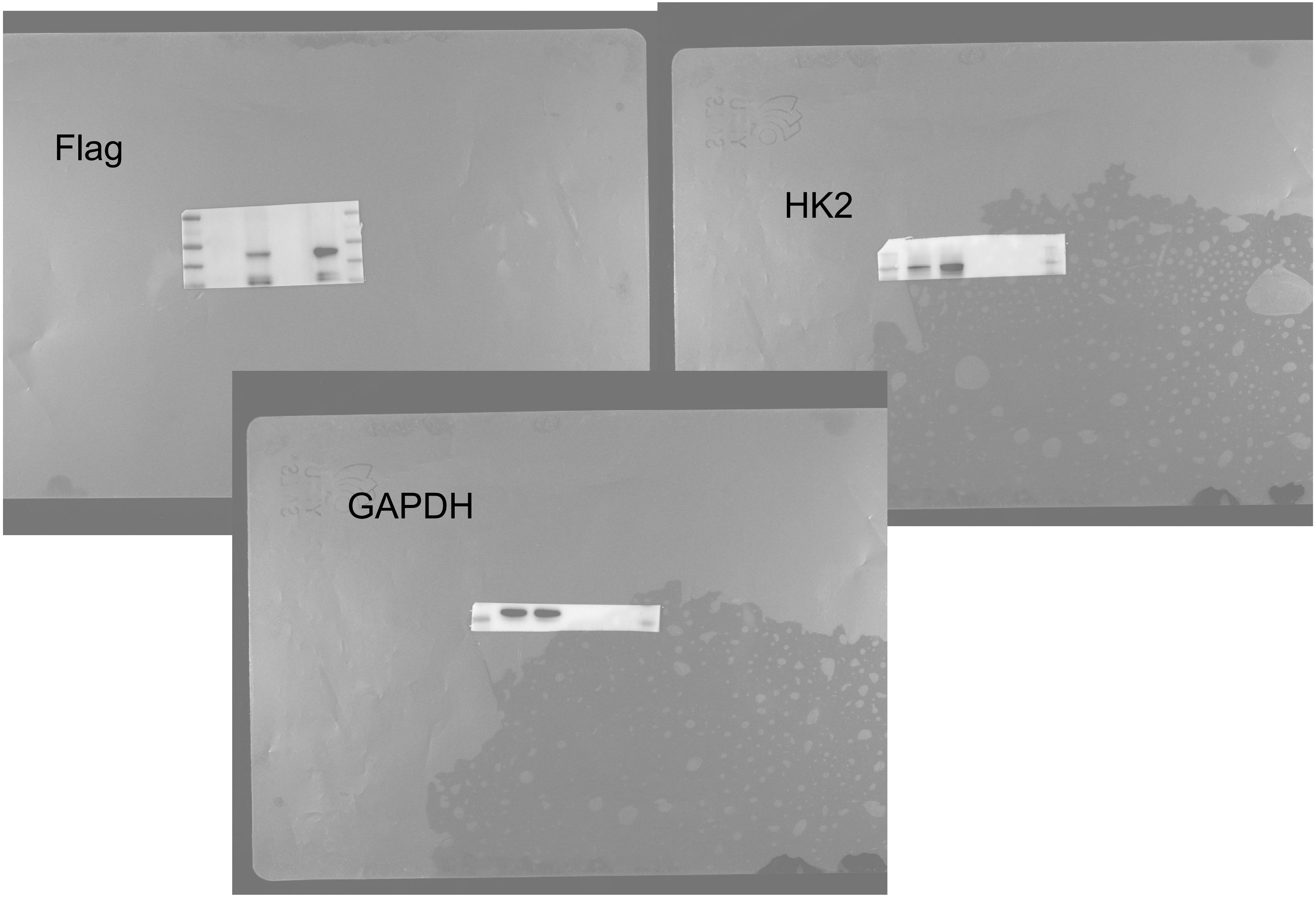

Supplement: Supplementary file 2 — Highlights [file 41419_2024_6835_MOESM2_ESM.pdf]
